# Dynamic Brain Lipid Profiles Modulate Microglial Lipid Droplet Accumulation and Inflammation Under Ischemic Conditions in Mice

**DOI:** 10.1002/advs.202306863

**Published:** 2024-09-09

**Authors:** Wei Wei, Seyed Siyawasch Justus Lattau, Wenqiang Xin, Yongli Pan, Lars Tatenhorst, Lin Zhang, Irina Graf, Yaoyun Kuang, Xuan Zheng, Zhongnan Hao, Aurel Popa‐Wagner, Stefan T. Gerner, Sabine Huber, Manuel Nietert, Christian Klose, Ertugrul Kilic, Dirk M. Hermann, Mathias Bähr, Hagen B. Huttner, Hua Liu, Dirk Fitzner, Thorsten R. Doeppner

**Affiliations:** ^1^ Department of Neurology University Medicine Göttingen (UMG) University of Göttingen 37075 Göttingen Germany; ^2^ Department of Neurology The Affiliated Hospital of Southwest Jiaotong University & The Third People's Hospital of Chengdu Chengdu Sichuan 610031 China; ^3^ Department of Neurology University Hospital Essen University of Duisburg‐Essen 45147 Essen Germany; ^4^ Department of Neurology University of Giessen Medical School 35392 Giessen Germany; ^5^ Department of Medical Bioinformatics UMG University of Göttingen 37075 Göttingen Germany; ^6^ Lipotype GmbH 01307 Dresden Germany; ^7^ Department of Physiology Faculty of Medicine Istanbul Medeniyet University Istanbul 34720 Turkey; ^8^ Department of Anatomy and Cell Biology Medical University of Varna Varna 9002 Bulgaria; ^9^ Center for Mind Brain and Behavior (CMBB) University of Marburg and Justus Liebig University Giessen 35037 Giessen Germany; ^10^ Research Institute for Health Sciences and Technologies (SABITA) Medipol University Istanbul 34810 Turkey

**Keywords:** cholesterol metabolism, ischemic stroke, lipid droplets, lipid metabolism, lipidomics, microglia, neuroinflammation

## Abstract

Microglia are critically involved in post‐stroke inflammation affecting neurological outcomes. Lipid droplet (LD) accumulation in microglia results in a dysfunctional and pro‐inflammatory state in the aged brain and worsens the outcome of neuroinflammatory and neurodegenerative diseases. However, the role of LD‐rich microglia (LDRM) under stroke conditions is unknown. Using in vitro and in vivo stroke models, herein accumulation patterns of microglial LD and their corresponding microglial inflammatory signaling cascades are studied. Interactions between temporal and spatial dynamics of lipid profiles and microglial phenotypes in different post‐stroke brain regions are found. Hence, microglia display enhanced levels of LD accumulation and elevated perilipin 2 (PLIN2) expression patterns when exposed to hypoxia or stroke. Such LDRM exhibit high levels of TNF‐α, IL‐6, and IL‐1β as well as a pro‐inflammatory phenotype and differentially expressed lipid metabolism‐related genes. These post‐ischemic alterations result in distinct lipid profiles with spatial and temporal dynamics, especially with regard to cholesteryl ester and triacylglycerol levels, further exacerbating post‐ischemic inflammation. The present study sheds new light on the dynamic changes of brain lipid profiles and aggregation patterns of LD in microglia exposed to ischemia, demonstrating a mutual mechanism between microglial phenotype and function, which contributes to progression of brain injury.

## Graphical abstract

The mechanisms underlying lipid metabolism and their impact on microglia in the context of stroke need further studies. Using an innovative in vitro and in vivo approach, this study observes dynamic changes in the lipid profile of the ischemic brain associated with microglial function. The study sheds new light on how lipid droplet accumulation and concomitant changes of lipidomic profiles modulate post‐ischemic inflammation, providing insights into potential therapeutic targets for reducing brain damage after stroke.

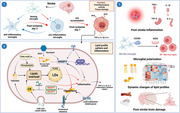

## 1 Introduction

Ischemic damage of the central nervous system (CNS) leads to acute functional impairment as a result of inadequate oxygen and nutrient supply.^[^ ^1^ ^]^ This process results in excitotoxic cell death, increased levels of reactive oxygen species (ROS), and activation of neuroinflammatory signaling cascades.^[^ ^2^ ^]^ Although ischemia‐associated inflammation may contribute to post‐stroke tissue regeneration per se, an overshooting inflammatory response rather contributes to acute tissue damage after ischemia.^[^ ^3^ ^]^ In this regard, microglia as the resident innate immune cells of the CNS play a pivotal role as effectors and modulators of post‐stroke inflammation,^[^ ^4^ ^]^ albeit their precise role still remains elusive. Using a well‐established rodent stroke model, the present work therefore also focused on analyzing spatial and temporal activation states of residing microglia that reflect the progression of the ischemic stroke lesion.^[^ ^4^, ^5^ ^]^ In this model, microglial activation states are assigned with their continuous spectrum from pro‐inflammatory toward anti‐inflammatory phenotypes by using different histologic markers.^[^ ^6^ ^]^

Beyond ischemia‐induced injury, there is increasing evidence that microglial activation plays a key role in post‐ischemic reperfusion injury in the brain.^[^ ^7^ ^]^ In this setting, lipid metabolism is emerging as a crucial modulator of microglial activity under various conditions. Previously, it has been shown that a lysosomal accumulation of cholesterol inside microglia significantly increases inflammatory activity and delays tissue regeneration in models of CNS demyelination, degeneration, and ischemia.^[^ ^8^ ^]^ In studies of aged brains, these microglia are referred to as lipid droplet‐rich microglia (LDRM).^[^ ^9^ ^]^ They are characterized by abnormal lipid accumulation, impaired phagocytosis, abnormal mitochondrial function, and elevated ROS. The underlying precise mechanisms, however, are still poorly understood.^[^ ^7^, ^9^, ^10^ ^]^

Therefore, our study analyzes the impact of lipid metabolism‐related changes and LD formation in microglia under in vitro and in vivo stroke conditions. Focusing on lipid profiles in specific brain areas as analyzed by state‐of‐the‐art lipidomics, the microglial phenotype transition, and inflammatory signaling pathways, we hypothesize that microglia exposed to ischemic‐hypoxic and inflammatory conditions after stroke convert into a pro‐inflammatory state accompanied by enhanced LD accumulation and distinct spatial and temporal brain tissue lipid profiles. Hence, modulation of LD biogenesis in microglia by altering lipid metabolism may improve functional outcomes in ischemic stroke.

## 2 Results

### 2.1 Hypoxia and LPS Induce LD Formation in Primary Microglia

**Figure 1:**
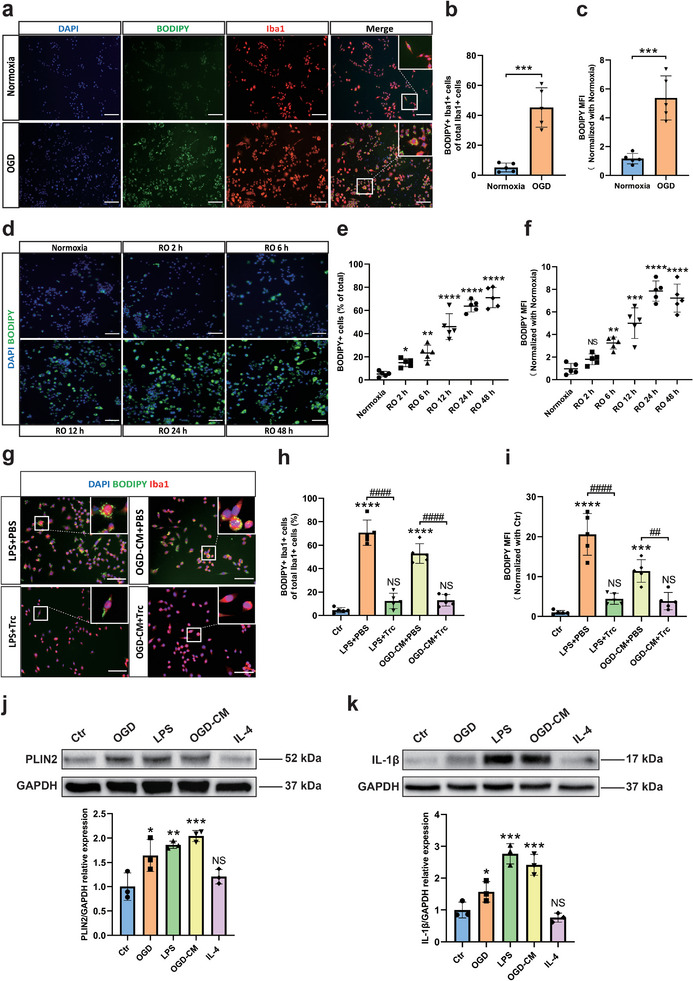
OGD, LPS, and CM induce accumulation of LD in primary microglia in vitro. a) Immunofluorescence staining of LD (BODIPY + green) accumulation in microglia after exposure to OGD. b,c) Quantification of the percentage of BODIPY + Iba1+ cells of total Iba1 + cells and BODIPY mean fluorescence intensity (MFI) (n = 5). d) Immunofluorescence staining of LD (BODIPY + green) accumulation in microglia after OGD according to the RO time (Normoxia, 2, 6, 12, 24, 48 h). e,f) Quantification of the percentage of BODIPY+ cells of total cells and BODIPY MFI (n = 5). g) Accumulation of LD in microglia with treatment of LPS or with CM from primary neurons after OGD (OGD‐CM) can be inhibited by Trc (1 µM, ACAT inhibitor). h,i) Quantification of the percentage of BODIPY+Iba1+ cells and MFI of BODIPY in five different conditions (n = 5). j,k) Quantitative analysis of PLIN2 and IL‐1β expression of microglia in five different conditions: normoxia as control, OGD, OGD‐CM, LPS, and IL‐4 using western blot analysis normalized with the housekeeping protein GAPDH (n = 3). Statistical tests: Two‐tailed t‐tests were used (b, c). One‐way ANOVA followed by Tukey's post‐hoc‐tests were used (e, f, h, i, j, k). Data are expressed as mean ± SD, NS: no significance, ^*^ *p <* 0.05, ^**^ *p* < 0.01, ^***^ *p* < 0.001, ^****^ *p* < 0.0001, ^###^ *p* < 0.001, and ^####^ *p* < 0.0001. Scale bars, 50 µm in (a, d, g). Ctr, Control group; OGD, oxygen‐glucose deprivation; RO, reoxygenation; OGD‐CM, conditioned medium from primary neuron after OGD; LD, lipid droplet; MFI, mean fluorescence intensity; Trc, triacsin C; ACAT, Acyl‐CoA cholesterol acyltransferase; PLIN2, perilipin 2; IL‐1β, interleukin 1 beta; IL‐4, interleukin 4; LPS, lipopolysaccharide.

First, we characterized the purity and features of primary microglial cultures by phase‐contrast microscopy and immunofluorescence stainings of microglial markers (Iba1, CD11b, CX3CR1, and TMEM119) (Figure S2a, Supporting Information). Thereafter, we determined the cell viability of primary neurons and microglia under both normoxic and hypoxic conditions, identifying 4 h of OGD and 24 h of RO as the optimal OGD/RO time (Figure S2b–e, Supporting Information). We then examined LD biogenesis in cultured microglia using a direct OGD model (exposure to hypoxia), an indirect OGD model (incubation of microglia with OGD‐CM; the conditioned medium was harvested from neurons that were exposed to OGD initially), and an LPS inflammation model. Exposure of microglia to hypoxia stimulated LD accumulation in Iba1‐positive microglia (**Figure** **1**a–c), and the extent of LD accumulation depended on the duration of RO peaking after 24 h of RO (Figure 1d–f). In analogy to the exposure of microglia to hypoxia, LD accumulation was also triggered by incubating microglia with LPS or OGD‐CM. Inhibition of the Acyl‐CoA cholesterol acyltransferase (ACAT) by treatment with Trc reduced the amount of LD accumulation (Figure 1g–i). Next, we quantified the expression of the LD‐associated protein PLIN2 (Perilipin‐2) and IL‐1β protein levels in microglia under different conditions by Western blotting. We found that, compared to controls, the PLIN2 expression was significantly elevated under both hypoxic and LPS stimulation, whereas it remained at low levels in the IL‐4 treatment group. Similar results were obtained for IL‐1β, especially in the LPS and OGD‐CM groups (Figure 1j,k).

### 2.2 MCAO Induces the Formation of LD in Microglia In Vivo

**Figure 2:**
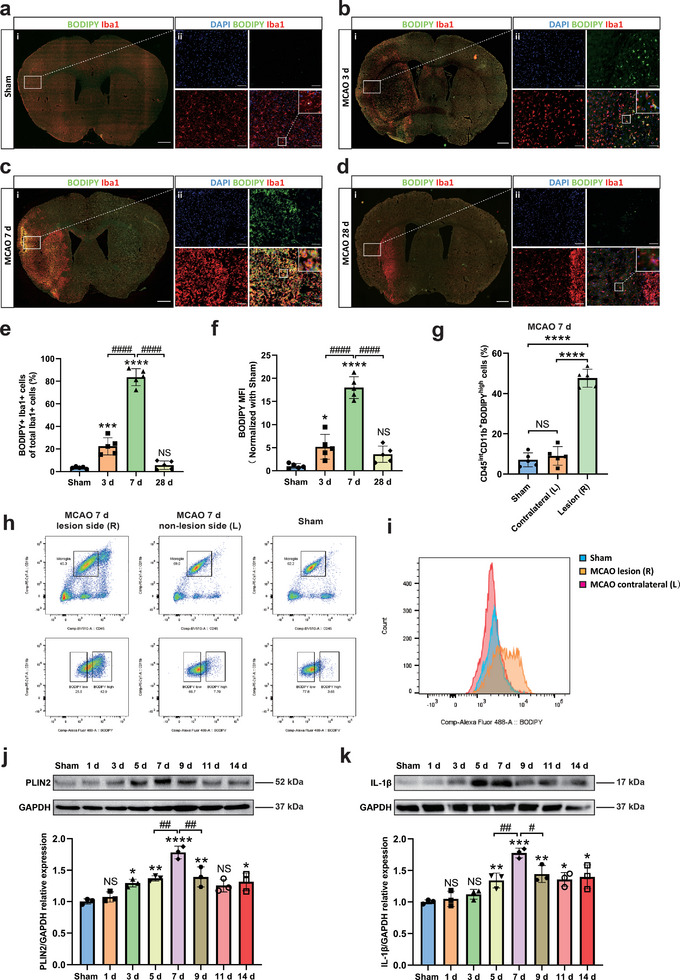
The formation of LD in microglia in the mouse middle cerebral artery occlusion (MCAO) stroke model. a–d) Immunofluorescence of cortical infarct core staining at different reperfusion times (sham, 3, 7, 28 days) after MCAO indicated that LD mainly accumulated in microglia. e,f) Quantification of the percentage of BODIPY+ Iba1+ cells and mean fluorescence intensity (MFI) of BODIPY (n = 5). g) Quantitative analysis of CD45intCD11b+BODIPY+ microglia in three groups (sham, ipsilateral, and contralateral side of post‐ischemia day 7) by flow cytometry (n = 7). h,i) Density plots of FACS showed a significant increase of LD‐rich microglia (CD45intCD11b+BODIPY+) within the MCAO lesion site group (h), which matched the BODIPY fluorescence intensity (i). j,k) Quantitative analysis of PLIN2 and IL‐1β expression in MCAO mice with different reperfusion times (sham, 1, 3, 5, 7, 9, 11, and 14 days) by Western blot analysis of the ischemic hemisphere. Western blot was normalized with the housekeeping protein GAPDH (n = 3). Statistical tests: One‐way ANOVA followed by Tukey's post‐hoc‐tests were used for (e‐g, j, k). Data are expressed as mean ± SD, NS: no significance, ^*^ *p <* 0.05, ^**^ *p* < 0.01, ^***^ *p* < 0.001, ^****^ *p* < 0.0001, ^#^ *p* < 0.05, ^##^ *p* < 0.01, and ^####^ *p* < 0.0001. Scale bars, 1000 µm (i) and 50 µm (ii) in (a, b, c, d). MCAO, middle cerebral artery occlusion; LD, lipid droplet; MFI, mean fluorescence intensity; FACS, Fluorescence‐activated cell sorting assay; PLIN2, perilipin 2; IL‐1β, interleukin 1 beta.

Using the MCAO stroke model, temporally and spatially resolved patterns of microglial LD accumulation were assessed in vivo. Cerebral ischemia due to MCAO resulted in an increased number of Iba1‐positive infiltrating cells accumulating LD within the ipsilateral lesioned hemisphere comprising of cortical and subcortical brain regions from days 3 to 7 post‐stroke (**Figure** **2**a–f). Thereafter, microglial cells were characterized by various histological markers (Table S5, Supporting Information) and by different FACS approaches. By evaluating the distribution of microglial cells in different regions of the lesion as well as the LD localization, the vast majority of BODIPY‐positive particles (Figure S3a–c, Supporting Information) or PLIN2‐positive subcellular structures (Figure S4a–c, Supporting Information) were co‐localized with Iba1‐positive cells and were mainly located within the post‐ischemic core region at day 7. Meanwhile, resting microglia labeled by P2y12 or TMEM119 were mainly located in non‐lesioned regions after stroke, whereas these cells disappeared from the lesion core at day 7 (Figures S5 and S6, Supporting Information). Using a flow cytometry approach, we were able to show that the number of LDRM significantly increased 7 days post‐ischemia in the lesion site (Figure 2g–i). In addition, the total number of cells labeled by CD68 or CD206 (inflammatory/anti‐inflammatory microglia) also gradually increased within 7 days after ischemia in the lesioned hemisphere (Figure S7a–d, Supporting Information). The gating strategy and representative density plots are shown in Figure S8a–c (Supporting Information). Next, we measured the protein expression levels of PLIN2 and IL‐1β at different post‐ischemic time points after MCAO by Western blotting. The expression levels of both PLIN2 and the pro‐inflammatory cytokine IL‐1β gradually and synchronously increased after MCAO within the ischemic lesion site, peaking at post‐ischemia day 7. Interestingly, such an up‐regulation was followed by a gradual decrease toward 14 days post‐ischemia (Figure 2j,k).

### 2.3 Effect of LDRM on Post‐Hypoxia Neuronal Survival in a Co‐Culture Model

**Figure 3:**
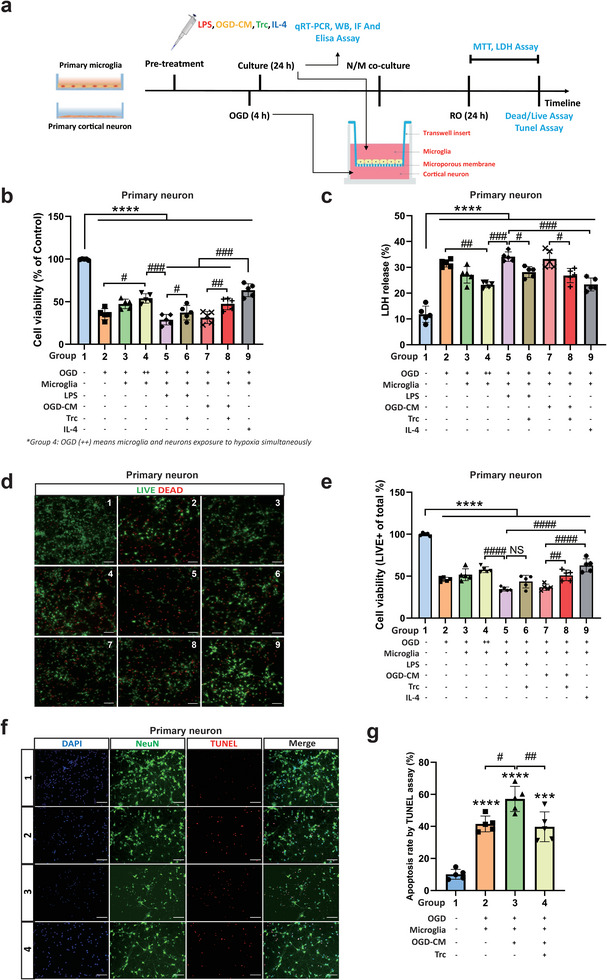
Primary microglia (PM) with different treatments affect the cell viability of primary neurons (PNs) after OGD in a co‐culture system. a) Schematic diagram of the PM/PNs co‐culture system. b) Cell viability was analyzed in PNs exposed to 4 h of OGD followed by 24 h of reoxygenation with co‐cultures of pre‐treated PM by MTT assay (n = 5). Cells incubated under normoxic conditions were defined as 100% cell survival. c) OGD‐induced neuronal cell toxicity was further assessed in the lactate dehydrogenase (LDH) release assay (n = 5). d,e) The LIVE/DEAD assay displays representative immunofluorescence stainings of calcein AM (LIVE cells, green) and ethidium homodimer‐1 (DEAD cells, red), followed by cell viability analysis via the percentage of LIVE cells to total cells (n = 5). The assay used the same conditions for PM and PNs as mentioned for the MTT assay. f,g) Immunofluorescence stainings and quantitative analysis of apoptotic cell (TUNEL, red) rate in PNs (NeuN, green) by TUNEL assay in the four groups: normoxia, OGD, OGD followed by co‐culture with OGD‐CM pre‐treated microglia, and OGD followed by co‐culture with OGD‐CM+Trc pre‐treated microglia (n = 5). Statistical tests: One‐way ANOVA followed by Tukey's post‐hoc‐tests were used for (b, c, e, g). Data are expressed as mean ± SD, NS: no significance, ^*^ *p* < 0.05, ^**^ *p* < 0.01, ^***^ *p* < 0.001, ^****^ *p* < 0.0001, ^#^ *p* < 0.05, ^##^ *p* < 0.01, ^###^ *p* < 0.001, and ^####^ *p* < 0.0001. Scale bars, 20 µm in (d, f). OGD, oxygen‐glucose deprivation; RO, reoxygenation; PM, primary microglia; PN, primary neuron; OGD‐CM, conditioned medium from primary neuron after OGD; LD, lipid droplet; IF, Immunofluorescence staining; WB, Western blot assay; MFI, mean fluorescence intensity; Trc, triacsin C; IL‐4, interleukin 4; LPS, lipopolysaccharide; Trc, triacsin C, LDH, lactate dehydrogenase.

Subsequently, we evaluated the effect of LDRM on neuronal survival under hypoxic conditions. Therefore, we designed a neuron–microglia co‐culture system in which different preconditioned microglia were co‐cultured with neurons after OGD/RO according to the experimental paradigm (**Figure** **3**a). Whereas both untreated microglia and IL‐4 pretreated microglia exerted protective effects on post‐hypoxic neurons, microglia pretreated with LPS or OGD‐CM did not yield any neuroprotective effect (Figure 3b). Interestingly, inhibition of LD formation after LPS or OGD‐CM stimulation due to the ACAT inhibitor Trc resulted in higher neuronal cell viability rates, indicating the detrimental effect of LDRM on neuronal survival under hypoxia. The aforementioned MTT data were confirmed in the LDH cytotoxicity release assay (Figure 3c), the LIVE/DEAD morphological assay (Figure 3d,e), and the TUNEL apoptosis assay (Figure 3f–g). These results demonstrate LDRM formed by either LPS or OGD‐CM increase neuronal damage in the neuron‐microglia co‐culture model, whereas Trc reduces this LDRM‐induced neuronal injury. Thus, our results indicate that microglial LD accumulation may affect neuronal survival under hypoxic conditions.

### 2.4 Altered Lipid Metabolism Related Genes (LMRGs) Correlate with Phenotype Polarization and Inflammatory Characteristics in Primary Microglia

**Figure 4:**
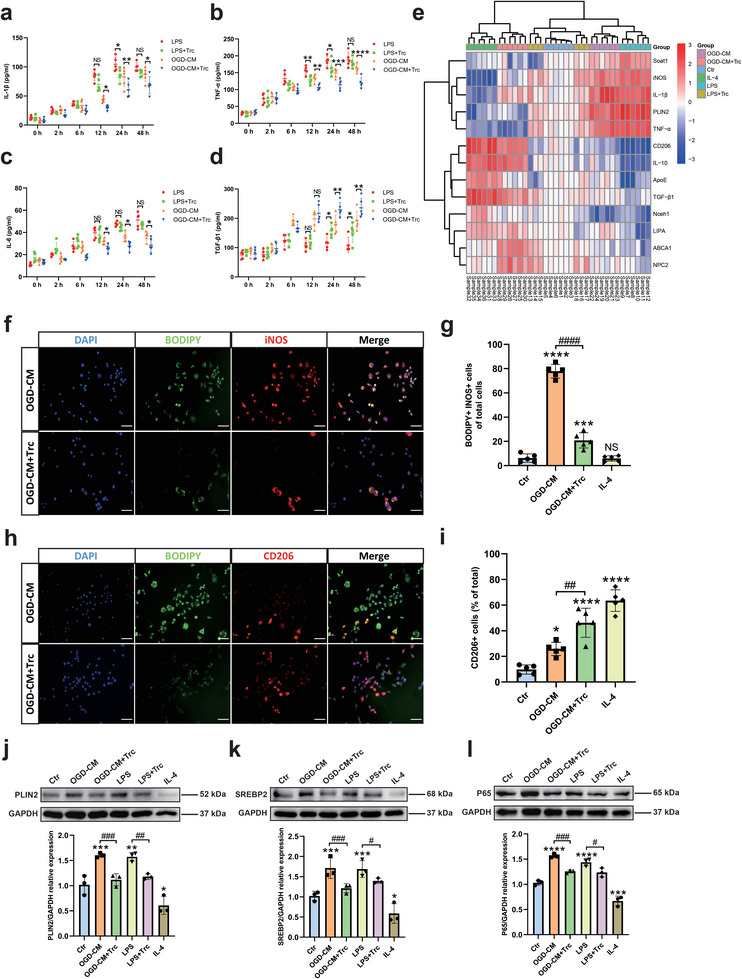
Lipid droplet‐rich microglia (LDRM) exhibit distinct lipid metabolism‐related gene expression as well as phenotype polarization and inflammatory factor levels after stimulation with LPS or OGD‐conditioned medium (OGD‐CM). a–d) Quantitative measurement of pro‐inflammatory factors (IL‐1β, TNF‐α, and IL‐6) and anti‐inflammatory factors (TGF‐β1) in microglia treated with LPS or OGD‐CM (w/ or w/o Trc) followed by different incubation periods (2, 6, 12, 24, and 48 h) using ELISA assay (n = 5). e) Heatmap of gene expression comparisons between six groups: control, LPS, LPS+Trc; OGD‐CM, OGD‐CM+Trc, and IL‐4. The heatmap was produced by pheatmap package in Rstudio. Data were log2 transformed and shown as red to blue: red (up‐regulated), blue (down‐regulated) and white (no change). Rows and columns are clustered using correlation distances and average associations. f Immunofluorescence co‐staining of LD (BODIPY, green) and M1‐like (iNOS, red) polarization of microglia in four different treatments: Control, OGD‐CM, OGD‐CM+Trc, IL‐4. g) Quantitative analysis of BODIPY+iNOS+ microglia of total cells (n = 5). h) Immunofluorescence co‐staining of LD (BODIPY, green) and M2‐like (CD206, red) polarization of microglia in the aforementioned four groups. i) Quantitative analysis of BODIPY+ microglia of total cells (n = 5). j–l) Quantitative analysis of PLIN2 (j), SREBP2 (k), and p65 (l) expression in six groups: normoxia as control group, OGD‐CM, LPS, LPS+Trc, OGD‐CM+Trc and IL‐4 group using Western blot analysis normalized with the housekeeping protein GAPDH (n = 3). Statistical tests: Two‐way ANOVA followed by Tukey's post‐hoc‐tests were used for (a‐d). One‐way ANOVA followed by Tukey's post‐hoc‐tests were used for (g, I, j‐l). Data are expressed as mean ± SD, NS: no significance, ^*^ *p <* 0.05, ^**^ *p* < 0.01, ^***^ *p* < 0.001, ^****^ *p* < 0.0*p < 0.05, **p < 0.01, ***p < 0.001, ****p < 0.0001, ^#^ *p* < 0.05, ^##^ *p* < 0.01, ^###^ *p* < 0.001, and ^####^ *p* < 0.0001. Scale bars, 20 µm (f, h). Ctr, Control group; OGD, oxygen‐glucose deprivation; RO, reoxygenation; OGD‐CM, conditioned medium from primary neuron after OGD; LD, lipid droplet; LDRM, lipid droplet‐rich microglia; Trc, triacsin C; PLIN2, perilipin 2; IL‐1β, interleukin 1 beta; IL‐4, interleukin 4; SREBP2, sterol regulatory element‐binding protein 2.

Our study demonstrates that LDRM exacerbates neuronal damage after hypoxia. We therefore intended to examine whether or not microglial LD formation under hypoxic conditions is associated with an altered inflammatory activation of microglia. Using an ELISA assay to detect the levels of pro‐ and anti‐inflammatory factors in primary microglia, levels of IL‐1β, TNF‐α, and IL‐6 were higher in LDRM under LPS and OGD‐CM stimulation than in control and IL‐4 treated groups. This was not the case for TGF‐β1 secretion, an anti‐inflammatory cytokine that predominantly was released upon IL‐4 stimulation (Figure S9a–d, Supporting Information). We also analyzed the levels of these cytokines in LDRM with and without Trc treatment, respectively. Within 48 h of incubation, IL‐1β and TNF‐α levels were consistently elevated in the LPS and OGD‐CM groups and reached a peak at 24 h, while a decrease in IL‐1β and TNF‐α levels was observed in Trc‐treated groups (**Figure** **4**a,b). Furthermore, although a slight increase in IL‐6 over time was observed in the LDRM groups, Trc did not significantly reduce the levels of IL‐6 in LPS‐stimulated microglia whereas IL‐6 levels dropped in OGD‐CM stimulated microglia after Trc treatment (Figure 4c). On the other hand, we observed that TGF‐β1 levels in the OGD‐CM group first tended to increase and peaked at 12 h, after which they decreased, whereas Trc treatment significantly increased TGF‐β1 levels in both, LPS and OGD‐CM stimulated groups, after 24 h and 48 h (Figure 4d). Subsequently, we analyzed post‐ischemic lipid metabolism‐related and inflammation‐related differentially expressed genes (DEGs), including the LD‐coating protein PLIN2, fatty acid synthesis and cholesteryl esterification (Soat1, SREBP2), fatty acid and cholesterol hydrolysis (Nceh1, LIPA, NPC2), lipid transport (ApoE, ABCA1), oxidative stress and microglial cell phenotype (iNOS, CD206), inflammatory factors (TNF‐α, IL‐1β), and anti‐inflammatory factors (IL‐10, TGF‐β1). We observed that PLIN2, Soat1, iNOS, IL‐1β, and TNF‐α were up‐regulated in LDRM (LPS and OGD‐CM stimulation), whereas CD206, ApoE, ABCA1, NPC2, Nceh1, LIPA, TGF‐β1 and IL‐10 were down‐regulated. In contrast, IL‐4‐stimulated microglia showed a different transcriptional profile than LDRM, with up‐regulated CD206, TGF‐β1, IL‐10, ApoE, Nceh1, and LIPA as well as with down‐regulated iNOS, IL‐1β, TNF‐α, Soat1 and PLIN2. We also found that the inhibition of LD formation in LDRM by Trc attenuated the up‐regulation of IL‐1β, TNF‐α, iNOS, PLIN2, and Soat1 in the OGD‐CM group. However, the alteration of these DEGs by Trc was less significant in the LPS group (Figure 4e).

We further used immunofluorescence staining to evaluate markers of microglial activation states, such as iNOS or CD206 to identify a pro‐ as well as a rather anti‐inflammatory activation state in the context of microglial LD accumulation, respectively. We observed that most OGD‐CM treated microglia were iNOS+ cells, whereas a few microglia were CD206+ microglia. In addition, Trc treatment resulted in decreased numbers of iNOS+ microglia and increased the number of CD206+ microglia. After stimulation with IL‐4, the vast majority of microglial cells exhibited a CD206 positivity (Figure S9e–g, Supporting Information). Next, we labeled LD with the fluorescent dye BODIPY and found that OGD‐CM stimulation induced BODIPY+ iNOS+ microglia. Trc treatment not only reduced the formation of LD but also the number of iNOS+ microglia (Figure 4f,g). Moreover, the number of CD206+ microglia remained low in the OGD‐CM treated group while CD206+ microglial cells were rarely associated with BODIPY+ LD detection. In contrast, a significant increase in CD206+ microglia was observed after inhibition of LD formation by Trc treatment (Figure 4h,i). We observed few iNOS+ and BODIPY+ microglia in the control and IL‐4‐group whereas most CD206+ microglia did not show LD staining by BODIPY (Figure S9h,i, Supporting Information). We further verified the expression of LD‐associated PLIN2, SREBP2, and p65 (a key factor of the NF‐κB pathway) at the protein level in microglia by Western blotting. Interestingly, p65, PLIN2, and SREBP2 were highly expressed in the LDRM of LPS and OGD‐CM stimulated groups as compared to the control group. Trc treatment attenuated this up‐regulation while IL‐4 stimulation resulted in the lowest expression levels of the aforementioned proteins (Figure 4j–l). Furthermore, we found that phagocytosis was impaired and ROS levels were higher in these LDRM, as assessed by microsphere uptake (Figure S10a,b, Supporting Information) and CellROX assays (Figure S10c,d, Supporting Information). Trc treatment of microglial cells resulted in an improved phagocytic capacity as well as lower ROS levels. We used enzymatic assays to assess the concentration of free fatty acids, cholesterol, and cholesteryl esters in LDRM indicating that stimulated cells had higher levels of free fatty acids and cholesteryl esters (Figure S11a–d, Supporting Information). Taken together, our results indicate, that LD formation and alterations of LMRGs in microglial cells under hypoxic conditions may modulate the microglial phenotype and activation state.

### 2.5 MCAO‐Induced Microglial LD Formation Shows Spatial and Temporal Dynamics Accompanied by Differential Microglial Activation States

**Figure 5:**
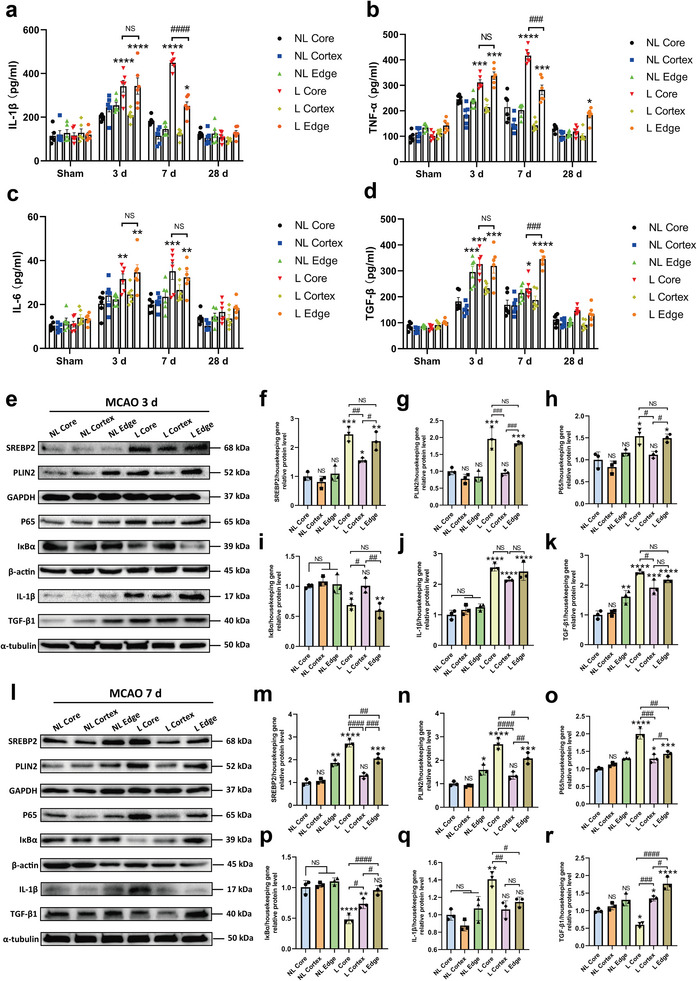
Ischemia‐induced up‐regulation of SREBP2 and PLIN2 leads to the formation of pro‐inflammatory lipid droplet‐rich microglia (LDRM) and the activation of the NF‐κB pathway. a–d) Quantitative measurement of IL‐1β (a), TNF‐α (b), IL‐6 (c) and TGF‐β1 (d) levels in the six regions: lesion core of cortex (L Core), ipsilateral cortex out of lesion (L Cortex), white matter area of lesion edge (L Edge), and corresponding areas on the contralateral non‐lesion side (NL Core, NL Cortex, and NL Edge) in the brain (sham, post‐ischemia 3, 7, and 28 d) using ELISA assay (n = 6). e–k) Quantitative analysis of SREBP2 (f), PLIN2 (g), p65 (h), IκBα (i), IL‐1β (j), and TGF‐β1 (k) expression in the aforementioned six regions with 3 days post‐ischemic mice by Western blot analysis normalized with the housekeeping protein (n = 3). i–r) Quantitative analysis of SREBP2 (m), PLIN2 (n), p65 (o), IκBα (p), IL‐1β (q), and TGF‐β1 (r) expression in the aforementioned six regions with 7 days post‐ischemic mice by Western blot analysis normalized with the housekeeping protein (n = 3). Statistical tests: Two‐way ANOVA followed by Tukey's post‐hoc‐tests were used for (a–d). One‐way ANOVA followed by Tukey's post‐hoc‐test was used for (f–k, m–r). Data are expressed as mean ± SD, NS: no significance, ^*^ *p* < 0.05, ^**^ *p* < 0.01, ^***^ *p* < 0.001, ^****^ *p* < 0.0001, ^#^ *p* < 0.05, ^##^ *p* < 0.01, ^###^ *p* < 0.001, and ^####^ *p* < 0.0001. L Core, lesion core of cortex; L Cortex, ipsilateral cortex out of lesion; L Edge, white matter area of lesion edge; NL Core, contralateral non‐lesion core of cortex; NL Cortex, contralateral non‐lesion cortex out of lesion; NL Edge, white matter area of contralateral non‐lesion edge; LD, lipid droplet; LDRM, lipid droplet‐rich microglia; PLIN2, perilipin 2; IL‐1β, interleukin 1 beta; IL‐6, interleukin 6; SREBP2, sterol regulatory element‐binding protein 2.

To further investigate the connection between the formation of LDRM and microglial activation states in different regions of the brain lesion after ischemia in vivo, we referred to the MCAO model. We defined six regions in the MCAO mouse brain: the ipsilateral cortex within the core of the lesion (L Core), the ipsilateral cortex outside of the lesion (L Cortex), the white matter at the edge of the ischemic lesion (L Edge), and the corresponding regions on the contralateral side (NL Core, NL Cortex, and NL Edge) (Figure S12a,b, Supporting Information). We analyzed the levels of various cytokines over time after ischemia in these brain regions by ELISA. The expression levels of inflammatory factors IL‐1β, TNF‐α, and IL‐6 were simultaneously increased in both the ipsilateral L Core and L Edge at 3 days post‐ischemia, whereas the levels of these factors in the L Cortex region were similar to the contralateral side. At 7 days post‐ischemia, the levels of IL‐1β and TNF‐α were significantly higher in the L Core region than in all other regions, including the L Edge (**Figure** **5**a–c). On the other hand, the expression levels of the anti‐inflammatory factor TGF‐β1 reached peak levels at 3 days post‐ischemia and kept a relatively high level in both L Core and L Edge regions. At 7 days post‐ischemia, compared to 3 days, the level of TGF‐β1 within the L Core region decreased and was significantly lower than in the L Edge (Figure 5d). The levels of all pro‐inflammatory and anti‐inflammatory factors recovered to the levels of the sham group at post‐ischemia 28 days. Hence, the dynamic changes of the microglial activation state as indicated by the profiles of these markers after ischemia occur mainly within one week after ischemia.

Subsequently, we measured the protein expression levels of SREBP2, PLIN2, P65, IκBα, IL‐1β, and TGF‐β1 in MCAO mice by Western blotting (Figure 5e,l). At 3 days post‐ischemia, SREBP2 protein expression levels were significantly up‐regulated in both L Core and L Edge regions (Figure 5f). PLIN2 and P65 protein expressions were also significantly up‐regulated in these two regions (Figure 5g,h). In contrast, IκBα showed lower levels in these two regions. (Figure 5i). In addition, compared with the NL side, both IL‐1β and TGF‐β1 showed higher protein levels on the lesion side (Figure 5j,k). At post‐ischemia day 7, the protein expression levels of SREBP2, PLIN2, P65, and IL‐1β in L Core continued to increase significantly (Figure 5m–o,q). In contrast, IκBα showed a lower expression level in the L Core region (Figure 5p). Finally, although TGF‐β1 expression level decreased in L Core, relatively high levels were maintained in the L Edge (Figure 5r). In accordance with the aforementioned in vivo findings, alterations of lipid metabolism in particular of sterol metabolism and markers of LD formation seem to be timely and spatially correlated to pro‐inflammatory processes in ischemic brain tissue. The NF‐κB signaling pathway might be a potential link between lipid metabolism and neuroinflammation in this regard.

### 2.6 Post‐Ischemia Phenotype Polarization Patterns of Microglia in Different Brain Regions

**Figure 6:**
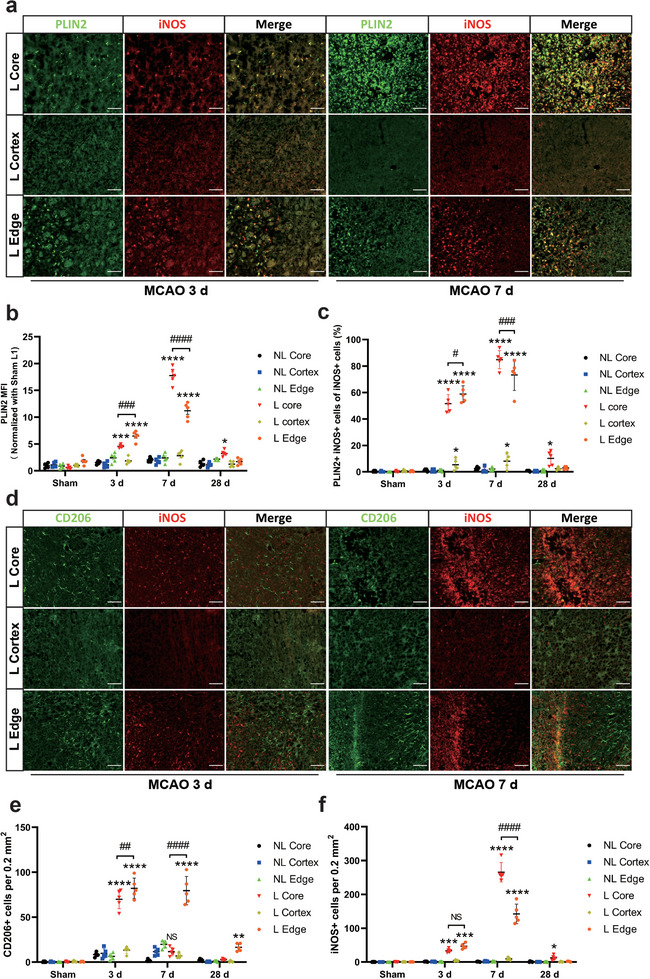
Phenotype patterns of PLIN2+ resident microglia in different lesion areas of the post‐stroke brain. a) Immunofluorescence staining of M1‐like microglia (iNOS, red) and LD surface protein marker (PLIN2, green) at post‐ischemia days 3 and 7 in the aforementioned six regions. b) Quantitative analysis of mean fluorescence intensity (MFI) of PLIN2 (n = 5). c) Quantitative analysis of the percentage of PLIN2+iNOS+ cells of total iNOS+ cells (n = 5). c Immunofluorescence staining of polarization of M1‐like microglia (iNOS, red) and M2‐like microglia (CD206, green) at post‐ischemia days 3 and 7 in the aforementioned six regions. d) Quantitative analysis of the number of iNOS+ and CD206+ cells per 0.2 mm^2^ area (n = 5). Statistical tests: Two‐way ANOVA followed by Tukey's post‐hoc‐tests were used for (b, c, e, f). Data are expressed as mean ± SD, NS: no significance, ^*^ *p* < 0.05, ^**^ *p* < 0.01, ^***^ *p* < 0.001, ^****^ *p* < 0.0001, ^#^ *p* < 0.05, ^##^ *p* < 0.01, ^###^ *p* < 0.001 and ^####^ *p* < 0.0001. Scale bars, 50 µm (a, d). MCAO, middle cerebral artery occlusion; LD, lipid droplet; PLIN2, perilipin 2; L Core, lesion core of cortex; L Cortex, ipsilateral cortex out of lesion; L Edge, white matter area of lesion edge; NL Core, contralateral non‐lesion core of cortex; NL Cortex, contralateral non‐lesion cortex out of lesion; NL Edge, white matter area of contralateral non‐lesion edge; MFI, mean fluorescence intensity.

We used PLIN2 staining to label LD and found that not only predominantly Iba1+ cells but also the majority of iNOS+ cells co‐localize with PLIN2+ cells. In particular, the percentage of iNOS+ cells was highest at 7 days post‐ischemia in L core followed by L edge. The mean fluorescence intensity of the PLIN2 staining increased gradually from 3 to 7 days post‐ischemia in the L Core and L Edge, whereas only rare iNOS+ or PLIN2+ cells were found in the L Cortex and NL regions (**Figure** **6**a–c). Both PLIN2 and iNOS expression gradually decreased to pre‐infarct levels at 28 days post‐ischemia (Figure S13a, Supporting Information). Additionally, our results showed that the polarization of microglia changes dynamically over time and varies according to infarct regions. In the L Core and L Edge regions at 3 days post‐ischemia, activated microglia were observed to be predominantly CD206+ cells, a marker traditionally associated with the so‐called M2‐like phenotype, although it has to be noted that also CD206+ macrophages have been reported within or in the proximity of CNS lesions. The number of iNOS+ microglial cells at 3 days post‐ischemia is significantly increased in L core and L edge, although being lower than the number of CD206+ microglial/macrophagic cells. In contrast, only a few CD206+ microglia/macrophages were observed in the L Cortex region. At 7 days post‐ischemia, the L Core region was infiltrated with a large number of iNOS+ microglia, while CD206+ cells were almost absent. Interestingly, the microglia found in the L Edge region were predominantly iNOS+ microglia close to the infarct lesion and CD206+ microglia/macrophages in the outer margin of L Edge region with iNOS representing a pro‐inflammatory and CD206 an anti‐inflammatory histological marker of microglial activation states (Figure 6d–f). At day 28 post‐ischemia, only limited CD206+ cells were present in the L Core and L Edge regions (Figure S13b, Supporting Information). Interestingly, we also found that both LD‐poor resting microglia and CD206+ microglia/macrophages frequently failed to co‐localize with PLIN2 (Figure S13c,d, Supporting Information). To summarize our findings regarding activation states of microglia and in particular LDRM within the lesion core in correlation with alterations of the lipid metabolism in the lesioned area we propose a temporal dynamic as visualized in Figure S14a–e (Supporting Information).

### 2.7 Definition of Differences in Lipid Classes within Different Brain Regions after Stroke

**Figure 7:**
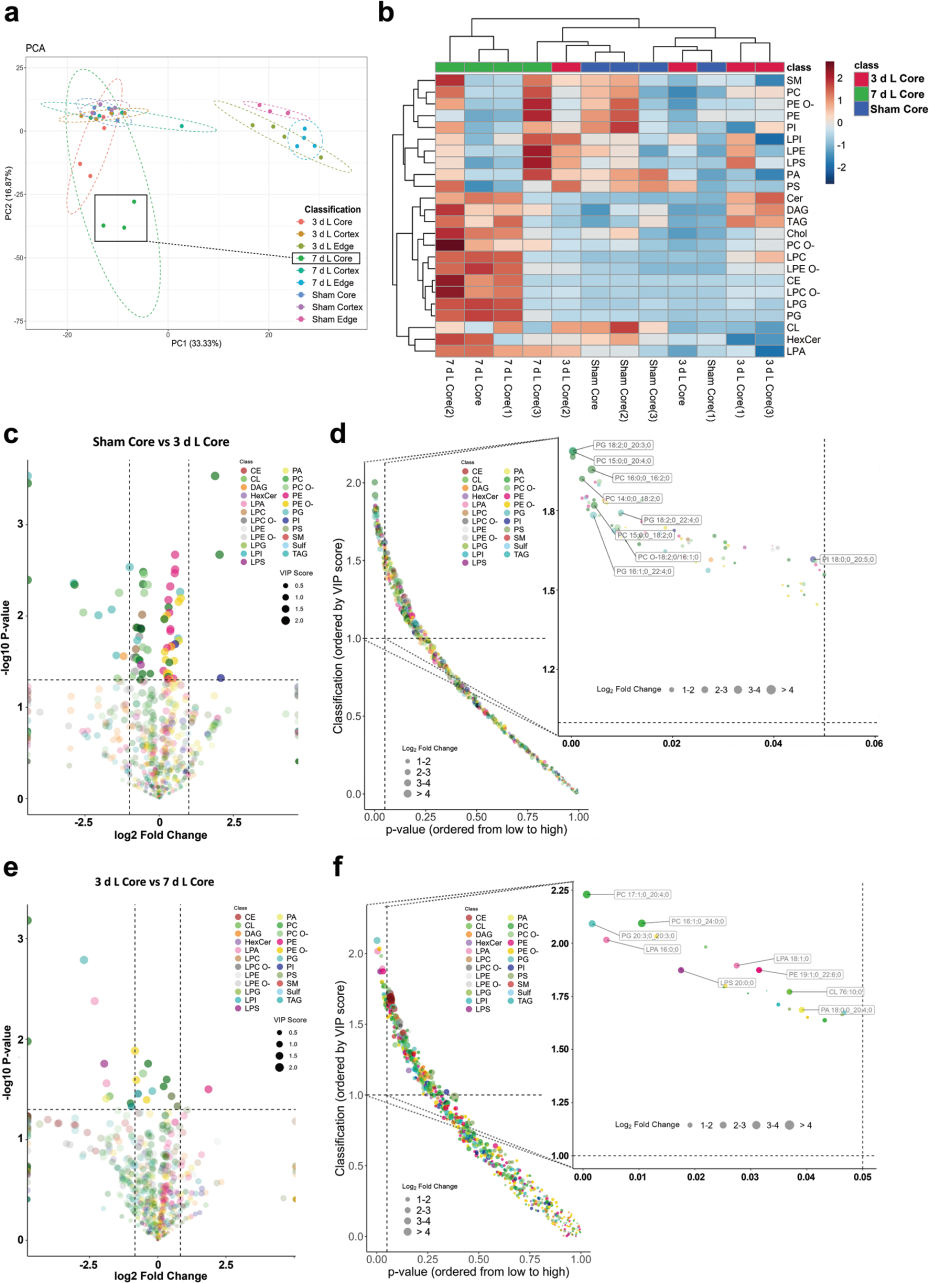
Lipid‐class composition of distinct mouse brain regions after stroke. a) Principal Component Analysis (PCA) of lipid composition patterns in different regions of brains (sham, post‐ischemia 3 days and 7) to identify the most important variables for lipid components. b) Heatmap for Core regions representing color‐coded Z scores of lipid‐class distribution of the 24 most abundant lipid classes using the lipid species concentrations (pmol) with log2 normalization. c) Volcano map of lipid profiles in sham core and 3 days L Core. d) Orthogonal partial least squares discriminant (OPLS‐DA) analysis of lipid profiles with VIP score in Sham Core and 3 days L Core. e) Volcano map of lipid profiles in 3 days L Core and 7 days L Core. f) OPLS‐DA analysis of lipid profiles with VIP score in 3 days L Core and 7 days L Core. Source data are provided as a source data file. PCA, Principal component analysis; OPLS‐DA, orthogonal partial least squares discriminant analysis; PLIN2, perilipin 2; L Core, lesion core of cortex; L Cortex, ipsilateral cortex out of lesion; L Edge, white matter area of lesion edge; CE, cholesteryl ester; Cer, ceramide; CL, cardiolipin; SM, sphingomyelin; DAG, diacylglycerol; TAG, triacylglycerol; PA, phosphatidate; PC, phosphatidylcholine; PC O‐, ether‐linked phosphatidyl‐choline; PE, phosphatidylethanolamine; PE O‐, ether‐linked phosphatidyl‐ethanolamine; PS, phosphatidyl‐serine; PI, phosphatidylinositol; PG, phosphatidylglycerol; LPA, lyso‐phosphatidate; LPC, lyso‐phosphatidyl‐choline; LPC O‐, ether‐linked lyso‐phosphatidyl‐choline; LPE, lyso‐phosphatidyl‐ethanolamine; LPE O‐, ether‐linked lyso‐phosphatidyl‐ethanolamine; LPI, lyso‐phosphatidyl‐inositol; LPG, lyso‐phosphatidyl‐glycerol; LPS, lyso‐phosphatidyl‐serine; Sulf, sulfatide; HexCer, hexosylceramide.

Since little is known about the impact of post‐stroke injury on brain lipid profiles, we used Shot‐Gun Mass Spectrometry Lipidomic analysis to detect changes in regional brain lipid composition after cerebral ischemia at different time points. Initial data exploration by Principal Component Analysis (PCA) of sham, days 3 and 7 post‐ischemia in each of the above‐mentioned brain regions revealed that, in addition to the regional separation, the 3 d L Core and the 7 d L Core appeared to have distinctive lipid profiles (**Figure** **7**a). In order to further specify which lipid classes have an influence on the differentiation of these groups we used a hierarchical clustering analysis of the L Core region. A cluster of increased concentrations of diglycerides (DAG), triglycerides (TAG), cholesterol (Chol), cholesteryl esters (CE), lysophosphatidylcholine (LPC), ether‐linked lysophosphatidylethanolamine (LPE O‐), ether‐linked LPC (LPC O‐), lysophosphatidylglycerol (LPG), and phosphatidylglycerol (PG) classes was observed at day 7 after ischemia when compared to sham animals. Additionally, the clustering of rows indicated, that the dynamics of two lipid clusters: ceramides (Cer), DAG, TAG, and Chol as well as CE, LPC O‐ and LPG showed similar dynamics over time (Figure 7b). To stratify and screen for relevant changes in the amounts of specific lipid species after cerebral infarction, we applied a combination of volcano plotting and machine learning‐based orthogonal partial least squares discriminant analysis (OPLS‐DA) as a supervised multivariate analysis. We found that lipid profile changes at post‐ischemia day 3 compared with sham were dominated by phosphatidylcholine (PC) and its ether variants (PC O‐) as well as phosphatidylethanolamine (PE) and its ether variants (PE O‐). Interestingly, PC and PC O‐ showed predominantly increased, while PE and PE O‐ exhibited decreased amounts on the third day after ischemic injury. Additionally, we observed changes in the composition of PG, with a notable increase by the 3rd day. Furthermore, there were isolated changes in LPC, LPE, and their ester derivatives, as well as LPS, which showed increasing amounts on the third day (Figure 7c,d). In the subsequent time course, comparing the 3rd and 7th‐day post‐ischemia, we also observed distinct patterns in the changes of PC and its ether variant (PC O‐), as well as PE and PE O‐. The concentrations of PC predominantly increased, indicating a progressive accumulation over time. In contrast, the changes in the ether variants of PC were isolated and did not follow the same increasing trend. On the other hand, PE continued to show a decrease in concentration, although only being sporadically measurable. Interestingly, the ether variant of PE O‐ exhibited an increasing trend, contrary to the overall decrease in PE concentration. The lipidome of 7 d L Core showed significant peaks of phosphatidate (PA, 18:0;0_20:4;0), PG (20:3;0_20:3;0), LPA (18:1;0), LPS (20:0;0), and cardiolipins (CL, 76:10;0) (Figure 7e,f). Following the same approach, we analyzed the Edge region using Volcano Plots and oPLS‐DA (Figure S15a–d, Supporting Information). We observed an increase of several subsets of CE, DAG, TAG, LPS, PG, and phosphatidylserine (PS) in the 3 d L edge region. Interestingly and in contrast to the core region species of the classes, DAG, TAG, PS, and PA decreased in the following days toward 7 days after ischemic damage in the edge region. Most impressive over time was the reduction of the initially elevated TAG and PS amount on day 7 after ischemia in the edge region. DAG showed a similar but lagging pattern initially increasing and from 3rd to 7th day partly decreasing amounts. The lipid species resulting from this analysis define the differences of the regions at 3 and 7 days after ischemia and are shown in Figure S16a–d (Supporting Information).

### 2.8 The Connection Between Lipid Profiles and Inflammation in the Post‐Ischemic Brain

**Figure 8:**
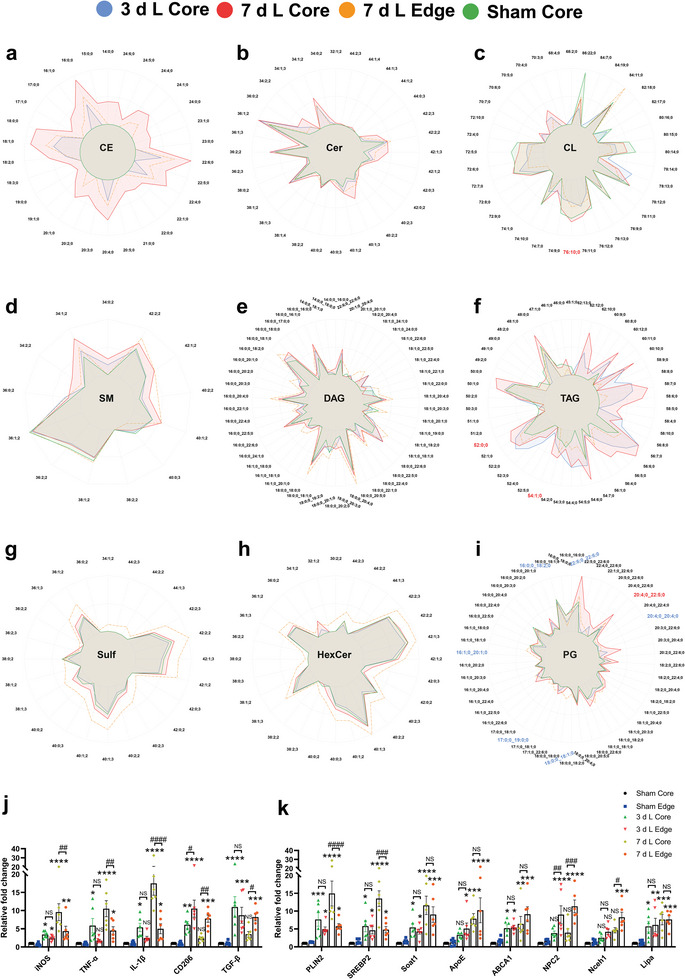
The connection between enrichment of lipid species and inflammation after stroke. a–i) The Radar plots display the logarithmic mean concentrations of lipids sorted by classes: cholesteryl ester (a), ceramide (b), cardiolipin (c), sphingomyelin (d), diacylglycerol (e), triacylglycerol (f), sulfatide (g), hexosylceramide (h), and phosphatidylglycerol (i). Each color represents different groups. The scale of the plot ranges from 0% to the highest concentration observed within each lipid class (100%). For lipid classes with over 50 sublipids, only the highest abundances are shown for clarity. The color scheme applied to the lipid subspecies names indicates significance based on the modified t‐test of the volcano plot (blue = *p* < 0.05 for comparison of Sham core versus 3 days L core; red = *p* < 0.05 for comparison of 3 days L core versus 7 days L core). j Dynamic changes of genes related to inflammation in different areas after MCAO (n = 6). k Dynamic changes of genes related to lipid metabolism in different areas after MCAO (n = 6). Data are expressed as mean ± SD, NS: no significance, ^*^ *p* < 0.05, ^**^ *p* < 0.01, ^***^ *p* < 0.001, ^****^ *p* < 0.0001, ^#^ *p* < 0.05, ^##^ *p* < 0.01, ^###^ *p* < 0.001 and ^####^ *p* < 0.0001. In each bar plot, p‐values < 0.05, VIP‐score > 1. CE, cholesteryl ester; Cer, ceramide; CL, cardiolipin; SM, sphingomyelin; DAG, diacylglycerol; TAG, triacylglycerol; Sulf, sulfatide; HexCer, hexosylceramide; PG, phosphatidylglycerol; PLIN2, perilipin 2; IL‐1β, interleukin 1 beta; L Core, lesion core of cortex; L Cortex, ipsilateral cortex out of lesion; L Edge, white matter area of lesion edge.

We further analyzed the link between dynamic changes in lipid profiles and microglial phenotype as well as post‐ischemic inflammation. Therefore, we intended to isolate spatial and temporal patterns of post‐ischemic lipid profiles and correlate these with transcriptional patterns. Based on previous statistical approaches we selected lipid classes showing dynamic changes and visualized the amount of lipid species in pmol in the relevant regions core and edge at 3 and 7 days post‐ischemia by radar plots. Strikingly, it became obvious that lipids associated with cholesterol metabolism, predominantly CE (16:0;0, 18:0;0, 18:1;0, 20:4;0, 22:4;0, 22:5;0, 22:6;0), which are very low or even undetectable in the sham core, however, are significantly increased in the core region 7 days post‐ischemia. Additionally, dynamic changes of CL species (70:4;0, 76:10;0, 78:13;0) are observable. Furthermore, Cer species (such as 34:1;2, 36:1;2, 36:2;2, 38:1;2, 42:1;3), sphingomyelin (SM) species (34:1;2, 40:1;2, 42:1;2, 42:2;2) and Chol appeared to be present in higher amounts in 7 d L Core than in any other group including the 7 d L Edge group (**Figure** **8**a–d). Interestingly, no significant changes were shown in all groups except for the DAG (18:0;0, 20:4;0), which was elevated in both 7 d L Core and Edge; whereas TAG (48:0;0, 50:0;0, 54:6;0, 56:6;0, 56:7;0) exhibited consistently increasing concentration levels in 3 d L Core and 7 d L Core and the fatty acid composition shifted from predominantly short/medium‐chain subsets to medium/long‐chain subsets (Figure 8e,f). Regarding TAG species a similar pattern was found transiently in 3 d L Edge while TAG amounts in 7 d L Edge returned to Sham levels. In contrast to previous lipid profiles, we observed that Sulf (36:1;2, 40:1;3, 42:2;2), HexCer (38:1;3, 40:1;3, 42:1;3, 42:2;3), and PG (22:6;0_22:6;0, 20:4;0_22:5;0) were relatively higher in the 7 day L Edge region (Figure 8g–i). Among other subsets of lyso‐phosphatidyl‐lipids, we found LPC (16:0;0, 18:0;0), LPC O‐ (16:1;0, 16:2;0, 18:1;0), LPE (18:0;0, 18:1;0), LPE O‐ (16:1;0, 18:1;0), LPA (16:0;0, 18:0;0, 18:1;0), LPS (20:0;0) and LPG (18:1;0, 20:4;0, 22:6;0) were highest in 7 d L Core, while they were not significantly altered in 7 d L Edge (Figure S17a–g, Supporting Information). In addition, most subgroups including PC, PC O‐, PE, PE O‐, PA (18:0;0_20:4;0), PI (18:0;0_20:5;0) and PS (18:0;0_22:4;0) were altered simultaneously at the infarct core and edge along with stroke progress and remained relatively high at 7 days post‐ischemia (Figure S18a–g, Supporting Information). The distribution of all lipid subsets is shown by additional bar plots in Figures S19–S28 (Supporting Information).

Additionally, we measured the transcriptional mRNA expression levels of LMRGs as well as inflammation‐related genes using RT‐qPCR (Figure 8j,k). The expression of iNOS, TNF‐α, IL‐1β, CD206, and TGF‐β1 showed an increase in both 3 d L Core and 3 d L Edge (dominated by CD206 and TGF‐β1), and there was no significant difference between them. In addition, the expression of iNOS, TNF‐α, and IL‐1β peaked in 7 d L Core and was significantly higher than in 7 d L Edge. However, the expression of CD206 and TGF‐β1 decreased in 7 d L Core and was significantly lower than that in 7 d L Edge. Meanwhile, we examined the expression of LMRGs and found that PLIN2, SREBP2, and Soat1, which are associated with LDs formation and CE synthesis, were up‐regulated in both 3 d L Core and 3 d L Edge, and without significant differences amongst them. Their expression peaked in 7 d L Core, and PLIN2 and SREBP2 were significantly higher than in 7 d L Edge. The expression of ApoE and ABCA1 continued to increase after ischemia and did not differ significantly between Core and Edge. NPC2 and Nceh1 expression in edge regions was up‐regulated after ischemia and significantly higher than in core regions. The expression of Lipa was up‐regulated in both, Core and Edge, after ischemia and without significant difference amongst these regions. Therefore, we suggest that temporal and spatial lipid profiles during the dynamic evolution of ischemic brain lesions, in particular with respect to cholesterol esters and storage lipids, can be correlated with regional patterns of lipid metabolism and, subsequently, with inflammatory activity in distinct areas of the lesion, indicating, that regional lipid composition might influence inflammatory processes during ischemic damage.

## 3 Discussion

Post‐ischemic inflammation plays a vital role in the progression of secondary brain injury after ischemic stroke. Despite its importance, the underlying mechanisms remain poorly understood.^[^ ^11^ ^]^ Elucidating these processes is essential to further improve stroke therapy in patients. In this context, LDRM displaying a dysfunctional, pro‐inflammatory phenotype of microglial cells may be key modulators of tissue damage and regeneration in various pathological conditions including ischemic damage.^[^ ^7^, ^12^ ^]^ In our study, we analyzed the role of post‐ischemic LD formation and brain lipid composition on the regulation of the microglial phenotype. Exposure of microglia to hypoxia yielded an accumulation of LD, a pro‐inflammatory microglial activation state, and neuronal cell injury in the co‐culture systems. Interestingly, treating LDRM with an inhibitor of ACAT not only reduced LD formation but also attenuated inflammatory activation and neuronal damage. In vivo we applied the MCAO model to demonstrate, that LDRM is a predominant phenotype of microglial cells displaying a temporal and spatial dynamic post‐ischemia within the lesion. We were able to observe the aligning dynamics of proinflammatory activation of microglial cells and alterations affecting lipid metabolism in the corresponding brain regions. The proinflammatory activation state of microglial cells, as detected by histology and FACS, in particular within the L core region at 7 days post‐ischemia appears to be associated not only with a marked accumulation of lipid droplets but also with a unique lipid profile in the ischemic lesion as determined by the state of the art lipidomic analysis. The latter showed persistently increased amounts of various lipid species such as cholesteryl esters and storage lipids like TAG and DAG. Interestingly, the lipid composition of ischemic lesion sites correlates with temporal and regional changes in microglial phenotypes, inflammation, LD accumulation, and lipid metabolism. As such, the brain tissue lipid composition might be a critical modulator of microglial activation states.

Ischemic brain damage not only results in acute neuronal damage but also leads to demyelinating damage.^[^ ^13^ ^]^ Myelin debris is phagocyted by resident phagocytes of the CNS, predominantly by microglial cells, and the phagocytic cargo is degraded in the lysosomal system, altering the cellular phenotype of phagocyte, e.g., by the formation of LDs or lipofuscin‐like lysosomal inclusions as observed during white matter aging.^[^ ^13a^ ^]^ In this regard, not only the phagocytic capacity but also the efficacy of the cellular lipid export machinery appears to be of relevance.^[^ ^13a^ ^]^ In reperfusion‐induced post‐ischemic injury, large amounts of myelin debris, lipoprotein particles, and lipids from dead cells are phagocyted or endocytosed by activated microglia.^[^ ^14^ ^]^ Elevated lipid synthesis and decreased lipid oxidation contribute to the accumulation of free fatty acids, ultimately propagating the formation of LDs. Interestingly, LD formation in microglial cells can also be triggered by stimulation with LPS,^[^ ^15^ ^]^ acting through toll‐like receptors and the NF‐κB pathway,^[^ ^16^ ^]^ a mechanism that was used in the present study as a control condition. LD accumulation can cause cells to secret various mediators of inflammation and trigger pathological processes that may be mediated by the mTOR/SREBP2 and NF‐κB/COX‐2 signaling pathways.^[^ ^17^ ^]^ Indeed, LD formation under ischemic conditions of the present work is accompanied by TNF‐α and IL‐1β production in microglia in vitro. Inhibition of LD formation in these LDRM significantly reduces microglia‐mediated inflammation and neuronal death in a neuron‐microglia co‐culture model. Hence, intracellular microglial lipid accumulation as indicated by LD formation appears to be not only a mere bystander of neuroinflammatory processes of ischemic lesions but rather a modulator of neuroinflammation mediated by cytokines such as TNF‐α and IL‐1β and indicated by the alteration of microglial phenotypes.

Accumulation of LD in so‐called LDRM, which can occur as so‐called “foamy microglia”, appears to reflect a microglial activation state, which includes an increased production of pro‐inflammatory cytokines and exacerbates post‐ischemic neuronal damage in vitro. Under such conditions, the observed up‐regulation of SREBP2, an activator of genes involved in cholesterol synthesis, can regulate fatty acid metabolism and inflammation. Lipid homeostasis is tightly regulated by an intercellular interplay involving microglia and astrocytes as well as neurons and oligodendrocytes. Interestingly, SREBP2, which is synthesized not only by microglia but also by astrocytes, has been shown to interact with NF‐κB signaling pathways.^[^ ^18^ ^]^ A previous study indicated that the inhibition or silencing of SREBP2 shifts the phenotype of microglia and also macrophages from a pro‐inflammatory to an anti‐inflammatory phenotype that promotes regeneration and healing.^[^ ^19^ ^]^ We hypothesize that LD formation induced by hypoxia might lead to acute inflammatory responses, up‐regulation of SREBP2, and subsequent activation of the NF‐κB signaling pathway. The latter further up‐regulates downstream inflammatory factors, including TNF‐α, IL‐1β, and IL‐6. Up‐regulation of SREBP2 also promotes ACAT‐mediated fatty acid synthesis and cholesteryl esterification, producing large amounts of unsaturated fatty acids and cholesteryl esters.^[^ ^20^ ^]^ This accumulation of lipids in microglia might lead to a “lipid overload”, which results in enhanced microglial LD formation indicating dramatic changes in the intracellular lipid composition and metabolism and ultimately disturbing intracellular lipid homeostasis within the period following ischemic damage. We were able to show that microglial cells accumulating LDs in the core and edge of ischemic lesions showed altered gene expression patterns in particular with regard to lipid metabolism, resulting in impaired lipid transport or efflux as well as subsequently up‐regulated inflammatory cytokines. Interestingly, the temporal and spatial dynamics of these expression patterns were strongly correlated with lesional lipid profiles as well as temporal and spatial inflammatory patterns within the lesion. Disturbances of the intralesional lipid homeostasis potentially exacerbated by above mentioned dysregulated lipid metabolism could subsequently result in facilitated formation of pro‐inflammatory LDRM and thereby further impair lesion recovery. To further strengthen our hypothesis, we applied a microglial cell culture system and found that indeed ACAT inhibition by Trc reduced the formation of LDs and led to a decreased expression of P65 as well as reduced release of inflammatory factors such as TNF‐α and IL‐1β in vitro, potentially induced by a relief of “lipid overload” induced by ACAT inhibition. Excess free cholesterol (FC) can be converted to CE by the ACAT. These esters can either be stored within the LD or can be exported from the cells to some extent.^[^ ^21^ ^]^ Interestingly, ablation of the ACAT1 genes leads to increased levels of 24(S)‐hydroxycholesterol which can be exported from cells and the CNS through the blood‐brain barrier reaching the periphery.^[^ ^22^ ^]^

Although strictly dividing microglia into M0, M1 and M2 microglia is certainly oversimplified and viewed as outdated in the field: M0‐like microglia are classified as either resting or harmonious physiological microglia^[^ ^23^ ^]^; M1‐like microglia are generally considered to bear pro‐inflammatory features^[^ ^24^ ^]^; whereas M2‐like microglia are associated with indicators of anti‐inflammatory or homeostatic functions and might be beneficial in terms of tissue recovery after stroke.^[^ ^2^, ^25^ ^]^ TMEM119^[^ ^26^ ^]^ and P2y12^[^ ^27^ ^]^ are recognized as excellent markers for identifying resting‐state microglia, which are rapidly lost in various pathological conditions. For instance, while P2y12 is present in steady‐state microglia in mice, it is completely lost in active and slowly expanding lesions.^[^ ^27^ ^]^ Although TMEM119 also downregulates upon activation, this process is slower and often incomplete. Therefore, TMEM119 and P2y12 are effective for identifying resting microglia in the initial and early stages of lesions.^[^ ^27a^ ^]^ Our findings support this view, suggesting that TMEM119 and P2y12 should not be used as markers for all microglial populations but specifically for resting microglial populations. Moreover, studies have demonstrated that TMEM119 is expressed on microglia but not on recruited macrophages.^[^ ^28^ ^]^ In our research, we observed that in active lesions caused by inflammatory neurological diseases, microglial and macrophage populations shift to pro‐inflammatory/anti‐inflammatory phenotypes, resulting in a significant reduction of the TMEM119+/P2y12+ phenotype. This reduction aligns with other indicators of pro‐inflammatory activation in the ischemic core, confirming that microglial activation and phenotypic changes are closely linked to the progression of inflammation following a stroke. A similar janus faced aspect can also be attributed to macrophagic cells infiltrating the CNS under various pathological conditions including stroke.^[^ ^29^ ^]^ In this regard, microglial and macrophagic cells in the CNS may show a partial overlap of activation markers such as for example CD206.^[^ ^30^ ^]^ The differentiation of infiltrating and resident monocytic cells can be difficult under certain conditions,^[^ ^31^ ^]^ however it has been shown previously that proliferation of parenchymal microglia is the main source of microgliosis after ischemic stroke.^[^ ^32^ ^]^ The polarization continuum of microglia cell populations may explain the highly versatile function of microglia in various pathological conditions. Many substances and mechanisms are involved in regulating microglia/macrophage polarization, amongst them LD accumulation and cholesterol content as well as the overall intracellular lipid composition have been shown to be of interest for microglia‐targeted therapy.^[^ ^9^, ^10^, ^33^ ^]^ As elaborated above, LDRM may be associated with prolonged and aggravated neuroinflammation, displaying temporal and spatial alignment with lesion evolution. We found an early loss of homeostatic microglial markers within the lesion while these markers were found to be up‐regulated at later time points and in particular in the marginal zone of lesions. Furthermore, microglial cells in the infarct core displayed distinct phenotypes at 3 and 7 days, possibly indicating separate waves of microglial activation and infiltration, with the LDRM cell population dominating the microglial infiltrate at day 7 in the lesion core. These results might indicate that the intracellular lipid composition, which in turn manifests itself in LD formation, may play a key role in regulating microglial phenotypes.^[^ ^22^ ^]^ Indeed, LD biogenesis in macrophages and microglia have a similar mechanism and both play a vital role in inflammatory progress.^[^ ^34^ ^]^ Another recent study likewise reported that ischemia‐induced LD aggregation of microglia in aged mice impairs stroke recovery and that this impairment can be mitigated by the renewal of young microglia lacking LD.^[^ ^7a^ ^]^

Meanwhile, our in vitro data showed that lipid synthesis‐related DEGs were up‐regulated in LDRM, whereas peroxisomal β‐oxidation, lipid hydrolysis, and lipid transport‐related DEGs were down‐regulated. As a consequence of these changes in lipid metabolism with elevated lipid synthesis and impaired degradation under ischemic conditions, a disturbed lipid homeostasis can be expected, potentially resulting in pro‐inflammatory activation of LDRM. As a matter of fact, the temporal resolution of M2‐like microglia under ischemic conditions ranges from 12 h to several days after ischemia.^[^ ^6b^ ^]^ Our in vivo studies indicated that microglia phenotypes change dynamically over time in the post‐ischemic brain. In the early post‐ischemic period, microglia began to migrate to the lesion and transitioned from a resting state to an activated state. With insufficient energy supply in the infarct core area, the energy metabolism mode of microglia changes from ATP to glycolysis, accompanied by up‐regulated de novo fatty acid synthesis,^[^ ^35^ ^]^ as well as down‐regulated β‐oxidation.^[^ ^36^ ^]^ Additionally, a large amount of fatty acids is released as cell debris and disintegrated myelin sheath to the extracellular space, which is finally phagocyted by microglia and potentially macrophages infiltrating the CNS, challenging the intracellular lipid homeostasis of phagocytes.^[^ ^37^ ^]^

Mass spectrometry imaging has been applied to analyze phospholipid composition in mouse CNS tissue after MCAO. This analysis of focal ischemic brain lesions was able to elucidate a remarkable temporal dynamic of multiple subsets of phospholipids as well as Cer, CE, and SM, which correlated with the early phase of acute damage and later phases of lesion resolution.^[^ ^8d^ ^]^ We performed state‐of‐the‐art shotgun mass spectrometry lipidomic analysis to evaluate brain tissue lipid profiles in ischemic lesions with temporal and spatial resolution. The subsets of PC, PC O‐, PE, and PE O‐ were massively altered in the early stages of the ischemic area, both in the core and at the edge of the lesion. Increased amounts of these lipid species might be associated with acute tissue damage and might be able to modulate initial reactive processes including inflammatory activation of microglial cells. Previous studies showed that ischemic damage changes the metabolome of the affected brain tissue, particularly with respect to fatty acid metabolism.^[^ ^38^ ^]^ LD are cell organelles that are hubs for lipid storage due to their high content of CE and TAG, but they also appear to modulate various cellular processes including inflammatory activation.^[^ ^7^, ^39^ ^]^ They are crucially involved in maintaining intracellular lipid homeostasis, especially in the context of cholesterol, since excess free cholesterol is partially converted to cholesterol esters and stored in LD.^[^ ^21^ ^]^ Strikingly, we were able to show that brain regions within the ischemic lesion containing larger numbers of pro‐inflammatory microglia also were enriched with LD. These regions predominantly located within the core showed a remarkable elevation of Chol, CE, DAG, and TAG at three and seven days post‐ischemia. Persistently increased amounts of these lipids were strongly correlated with microglial pro‐inflammatory phenotypes, LD accumulation, and a distinctive pattern of LMRG levels indicating that the tissue lipid composition and potentially the microglial lipid profile substantially influences the resolution of ischemic lesions. Confirming this assumption, we detected less microglial LD accumulation within the edge region and a rather anti‐inflammatory, homeostatic phenotype of microglial cells where levels of many TAG and CE species substantially dropped at seven days post‐ischemia. The relevance of such a lipid metabolism and changes in lipid profiles has also recently been demonstrated in human stroke.^[^ ^40^ ^]^ Hence, a persistent overload of multiple lipid species was strongly correlated with microglial pro‐inflammatory phenotypes, LD accumulation, and a distinctive pattern of LMRG levels indicating that the tissue lipid composition and potentially the microglial lipid profile substantially influences the resolution of ischemic lesions.

In conclusion, the present study demonstrates that ischemia induces dynamic alterations in the lipid profile of the brain. This contributes to the aggregation of LD in phagocytic cells/microglia and, in turn, modulates the microglial phenotype mediating post‐ischemic neuroinflammation. In addition, different infarct regions showed distinct lipid patterns, with dramatically elevated CE and TAG mainly concentrated in the lesion core region at 7 days after ischemia, which exhibited pro‐inflammatory microglial phenotype and elevated levels of inflammatory factors distinct from those in the lesion edge region. Thus, during the pathological progression of ischemic stroke, neuroinflammation is accompanied by changes in the lipid profile, and specific lipid species may be involved in modulating the microglial function in the inflammatory process. This new work emphasizes the importance of lipid metabolism homeostasis for inflammatory regulation under stroke conditions. However, the specific downstream signaling pathways at the intersection of lipid metabolism and inflammation need to be further characterized, and thus more research is needed before clinical translation.

## 4 Experimental Section

### Cell Cultures

Primary microglia cells were isolated from C57BL/6J newborn pups at postnatal 0–2 days, based on the protocol of Hong et al.^[^ ^41^ ^]^ The cortex and hippocampus were carefully separated and moved in a 15‐mL tube containing cold PBS and were then digested with 1 mL of 0.25% Trypsin‐EDTA for 15 min. Digestion was terminated with 5 mL primary microglia medium (DMEM‐F12 supplemented with 10% FBS and 1% penicillin/streptomycin). The cell suspension was centrifuged at 300x g for 5 min, and the pellet was gently suspended in 5 mL warm medium, thereafter. The homogeneous cell suspension was transferred to a T75 flask precoated with poly‐L‐ornithine (PLO). After 5 days, the cell culture medium was changed and treated with the murine macrophage colony‐stimulating factor (M‐CSF, Peprotech, Hamburg, Germany) to stimulate microglial proliferation. After another 4 days, the flask was shaken vigorously, and floating microglia were collected from the conditioned medium. The primary microglia were seeded at 40 000 cells cm^−2^ in PLO‐coated dishes or flasks for upcoming experiments.

Primary cortical neurons were prepared following the protocol from Thomas et al.^[^ ^42^ ^]^ Pregnant C57BL/6J female mice at embryonic day 16.5 were sacrificed by euthanasia. The brain of embryos was carefully dissociated from the skull, and the meninges were then carefully removed. The cerebral cortex and hippocampus were carefully isolated and moved into a 15‐mL tube with PBS on ice. The tissue at the bottom was gently mixed and digested with 1 mL of 0.25% Trypsin‐EDTA for 15 min incubation. Primary neuron cell culture medium (neurobasal medium supplemented with 2% B27, 1% penicillin/streptomycin, L‐glutamine, and additional transferrin) was added to stop the digestion. The homogeneous cell suspension with primary neurons was finally seeded on PLO‐coated plates at a density of 200 000 cells cm^−2^. Primary neuronal cells were cultured for another 5 days before being used for subsequent experiments.

### Oxygen–Glucose‐Deprivation (OGD)

The cells were exposed to OGD when their confluence reached 80–90%.^[^ ^2d^ ^]^ For the OGD procedure, cells were washed twice with PBS and incubated with the same volume of BSS0 solution (116 mM NaCl, 5.4 mM KCl, 0.8 mM MgSO_4_, 1 mM NaH_2_PO4H_2_O, 26.2 mM NaHCO_3_, 10 mM HEPES, 0.01 mM glycine and 1.8 mM CaCl_2_, pH 7.2–7.4) and transferred to the hypoxia chamber (Toepffer Lab Systems, Goeppingen, Germany), containing 0.2% O_2_, 5% CO_2_, and 70% humidity. After OGD, the BSS0 solution was removed. The cells were then washed once with PBS followed by an incubation with the original cell culture medium and a specific treatment with different test substances for 24 h of reoxygenation (RO) in the 5% CO_2_ standard cell culture incubator at 37 °C. Thereafter, the cells would be used for subsequent experiments.

### Middle Cerebral Artery Occlusion (MCAO) and Animal Groups

Legal issues, animal housing, randomization, and blinding are shown in the materials. The MCAO model was done as described previously.^[^ ^43^ ^]^ Briefly, male C57BL/6J mice (aged 10–12 weeks) were anesthetized with isoflurane. A silicon‐coated microfilament is used to block the right middle cerebral artery (MCA) blood flow. A Laser Speckle Imaging System (LSIS, RWD Life Science, Shenzhen, Guangdong, China) was applied to ensure a successful blood flow block, and cerebral blood flow parameters were recorded. After 60 min, the microfilament was removed to initiate reperfusion. The cerebral blood flow parameters were re‐recorded with the LSIS. The experimental design and schematic diagram of the MCAO surgery are shown in Figure S1a–c (Supporting Information), and cerebral blood flow parameters of the LSIS are shown in Figure S1d,e (Supporting Information). The survival rates of mice used in the experiment are given in Table S1 (Supporting Information).

### Treatment with different Test Substances of Primary Microglia

The cell culture medium of primary microglia was replaced the day after seeding. Substances were added to cell culture medium for 24 h. Lipopolysaccharide (LPS, Sigma–Aldrich Chemie, Taufkirchen, Germany), interleukin‐4 (IL‐4, Sigma–Aldrich Chemie, Taufkirchen, Germany), and Triacsin C (Trc, Cayman Chemical, Michigan, USA) were used as test substances. The control group was given an equal volume of PBS. Therefore, cells were divided into the following seven groups: group 1 (microglia treated with substance solvent under normoxic conditions); group 2 (OGD/RO treatment with substance solvent); group 3 (1000 ng mL^−1^ LPS treatment); group 4 (2 µM Trc treatment in group 3); group 5 (incubation with conditioned medium from neurons after OGD/RO), group 6 (2 µM Trc treatment in group 5) and group 7 (10 ng mL^−1^ recombinant IL‐4 treatment).

### Primary Microglia‐Neuron Co‐Culture System

The co‐culture model used primary microglia and primary neurons to study the effect of microglia on neuron survival under hypoxic conditions. The experiment was based on the protocol of Skaper et al.^[^ ^44^ ^]^ Primary microglia were seeded into 6‐well (4 × 10^5^ cells/insert) or 24‐well (2 × 10^4^ cells/insert) transwells (3 µm pore size; Costar, MA, USA). To certificate the protective effect of different pre‐treated microglia on post‐hypoxic neuronal survival, five microglial conditions were tested: LPS, OGD, conditioned medium from post‐OGD neurons (OGD‐CM), Trc, and IL‐4 as a positive control. After 24 h of treatment, these primary microglia were added to plates pre‐seeded with primary neurons for another 24 h RO. Microglia were co‐cultured with primary neurons at the start of RO.

### Cell Viability and Cytotoxicity Assay

Cell viability was measured via a colorimetric assay by using the MTT (Thiazolyl Blue Tetrazolium Bromide, Sigma‐Aldrich, St. Louis, MO) viability assay according to the protocol from Riss et al.^[^ ^45^ ^]^ After OGD/RO, cell viability was presented as relative changes in percent compared to untreated controls. Absorbance was measured with a Tecan Sunrise colorimetric microplate reader (Tecan Group AG, Männedorf, Switzerland) at a wavelength of 570 nm. The cell death rate was also determined morphologically by using a LIVE/DEAD Viability kit (Lonza, Basel, Switzerland) as directed by the instructions of the manufacturer. Living cells were identified with calcein AM (4 µM, green fluorescence), and dead cells were identified with ethidium homodimer 1 (2 µM, red fluorescence). Cytotoxicity was determined by the release of lactate dehydrogenase (LDH) from cells to detect levels of cytotoxicity. A 50 µL aliquot of medium was transferred to another new 96‐well plate for each group. An equivalent dose of the test reagent provided by the manufacturer was added to each well to measure the release of LDH from the cells. The optical absorbance was measured at a wavelength of 490 nm.

### Immunofluorescence and BODIPY Staining

Primary microglia were seeded on poly‐l‐lysine‐coated pre‐coated chambers at a density of 4 × 10^4^ cm^−2^. Cells were washed once with cold PBS and then fixed in 4% paraformaldehyde (PFA) for 20 min. The cells were washed three times with PBS, permeabilized with 0.25% Triton X‐100 for 15 min, and then washed three times. PBS with 10% DS (donkey serum) or 1% BSA (bovine serum albumin) was used as a blocking buffer for 1 h. Thereafter, an overnight incubation with primary antibodies (Iba1, iNOS, CD68, CD11b, p2y12, TMEM119, PLIN2, CD206 and CX3CR1) followed. After three times washing with PBS, the slides were incubated with the corresponding secondary antibody for 2 h or with BODIPY 493/503 for 30 min.

For in vivo tissue staining, post‐perfusion brain samples from C57BL/6J mice were fixed in 4% PFA for 24 h, dehydrated with 30% sucrose. Cryosections were blocked for 1 h, followed by overnight incubation with the following primary antibodies: NeuN, Iba1, iNOS, CD68, p2y12, TMEM119, PLIN2, CD206, Arginase I, and GFAP. After primary antibody incubation, sections were incubated with secondary antibodies for 2 h. For BODIPY staining, the method was referred to Marschallinger et al.^[^ ^9^ ^]^ Nuclei staining was then performed with 4′,6‐Diamidin‐2‐phenylindol (DAPI, 1:10000; AppliChem, Darmstadt, Germany). Finally, Vectashield (Vector Laboratories, H‐1000) was used for mounting. Specific antibody working concentrations are shown in Table S2 (Supporting Information).

### Western Blotting Analysis

The brain tissue samples and the cell samples were lysed in a solution buffer containing RIPA Lysis and Extraction Buffer (Thermo Scientific, Waltham, USA) with a homogenisator or sonicator for 10 min and subsequently centrifuged at 4 °C. Protein concentrations were quantified with the Pierce BCA protein assay kit (Thermo Fisher Scientific, USA). Equal amounts of protein were loaded on 8% to 12% SDS‐PAGE gel and electrophoretically separated in sample buffer (dithiothreitol, final 0.1 M concentration, 0.1% SDS, 0.1 M Tris HCl; pH 7.0). Then, the electrophoresis gel was transferred to a polyvinylidene fluoride membrane (Merck Group, Darmstadt, Germany) by the tank transfer protocol. After transfer, membranes were incubated in blocking buffer for 1 h followed by overnight incubation for optimal results with primary antibodies: PLIN2, Sterol regulatory element‐binding protein 2 (SREBP2), NF‐κB p65, IκBα, IL‐1β, TGF‐β1, β‐actin, α‐tubulin and GAPDH. Then, the blots were mixed with secondary antibodies (1:10000) for 1 h. Specific antibody working dilutions are given in Table S2 (Supporting Information). Image J version 1.60 was used to measure the grey value of each blot. Western blot measurements were performed in triplicates at least.

### Quantitative Real‐Time Polymerase Chain Reaction (qRT‐PCR)

To extract total RNA, TRIzol (Invitrogen, Darmstadt, Germany) was used according to the manufacturer's instructions. Total RNA concentration was determined with a NanoDrop ND1000 Spectrophotometer (Wilmington, DE, USA). mRNA was reversely transcribed to cDNA with the RevertAid H Minus First Strand cDNA Synthesis Kit, followed by qRT‐PCR with the SYBR Green I Master Kit for LightCycler 480 (Merck Group) according to the manufacturer's instructions. All PCR primers were purchased from Eurofins Genomics (Luxembourg, Germany). The sequences of all primers are shown in Table S3 (Supporting Information). The relative expression levels were calculated and quantified using the ^2−ΔΔ^CT method after normalization with the reference β‐actin. The reported results are based on at least three independent experiments performed on different batches of cells or mice.

### Enzyme‐Linked Immunosorbent Assay (ELISA)

Concentrations of TNF‐α, IL‐1β, IL‐6, and TGF‐β1 were determined with commercial ELISA kits (Thermo Fisher Scientific, USA) according to the manufacturer's instructions. 96‐well plates were coated with capture antibody at 100 µL/well. The plates were sealed and incubated overnight at 4 °C followed by blocking with 200 µL ELISA/elispot diluent (1X) for 1 h incubation. Standard samples were prepared as instruction for a standard curve, and 100 µL/well of diluted detection antibody was added to each well. The plate was sealed and incubated for 1 h. After aspirating and three to five times washing, 100 µL/well of diluted horseradish (Avidin‐HRP) was added to each well and incubated for 30 min. The absorbance of the samples was detected at 450 nm by the colorimetric reader.

### Apoptosis TUNEL Staining Assay

Terminal deoxynucleotidyl transferase dUTP nick end labeling (TUNEL, in situ cell death detection kit, Sigma–Aldrich) staining was used to detect cell death according to the manufacturer's instructions. After specific treatment, cells were fixed and permeabilized. Subsequently, a working reaction mixture was prepared with 50 µL total volume of enzyme solution into 450 µL of label solution to obtain a 500 µL TUNEL reaction mixture. Then, the cells were incubated with the TUNEL reaction mixture for 1 h at 37 °C in the dark. Afterward, DAPI staining was used to stain cell nuclei.

### Flow Cytometry Analysis

LDRM in the cerebral hemisphere after MCAO were determined by flow cytometry analysis. Ischemic cerebral hemispheres were mechanically homogenized in lysis buffer (0.5% BSA, 5% glucose, 10 mg mL^−1^ DNase in PBS) and centrifuged at 1600 rpm for 10 min. Thereafter, the pellets were dissolved in 30% Percoll solution (GE Healthcare, USA) and loaded onto a gradient containing 45% and 70% Percoll. After centrifugation, the cells were aspirated between stages and dissolved in working solution (3% fetal bovine serum in PBS). Before antibody labeling, the cell suspension was incubated with anti‐mouse Fc‐Block (final concentration of 2.5 µg mL^−1^) for 10 min at 4 °C to prevent non‐specific binding. After washing, cells were incubated with anti‐CD45, anti‐CD11b, and BODIPY (BioLegend, San Diego, USA) overnight. Flow cytometry quantification was obtained using FlowJo v. 10.8.1 (BD FACSDiva) software.

### Shotgun Mass Spectrometry Lipidomic Analysis

Mass spectrometry‐based lipid analysis was performed by Lipotype GmbH (Dresden, Germany) as described.^[^ ^46^ ^]^ The identification of the lipids is based on LipidXplorer.^[^ ^47^ ^]^ Only lipid identifications with a signal‐to‐noise ratio >5, and a signal intensity 5‐fold higher than in corresponding blank samples were considered for further data analysis.

### Quantitative Analysis of Immunofluorescence Data

The cortex and striatum were detected as regions of interest (ROI), five randomly selected fields of view per overlay were photographed, and three images were taken for each region. The average neuron or microglia density was determined for all ROIs. For cell slides, photographs were taken in three fields of view (20x magnification in vivo and 40x in vitro), and each overlay was randomly selected. For BODIPY+Iba1+ and PLIN2+iNOS+ microglia, images were analyzed blindly based on the number of co‐localized cells, and a quantitative analysis of the mean fluorescence intensity and the percentage of co‐localized cells as total cells was done. Immunofluorescence slides were photographed with a Zeiss Axioplan 2 fluorescence microscope (Zeiss, Oberkochen, Germany) or a confocal scanning laser microscope (Zeiss LSM 700, Zeiss, Germany). Images were processed with ZEN software version 3.20. Cell colocalization analysis and fluorescence intensity quantification were performed with ImageJ software version 1.60.

### Statistical Analysis

For comparison of two groups, the two‐tailed independent Student's t‐test was used. For comparison of three or more groups, a one‐way analysis of variance (ANOVA) followed by Tukey's post‐hoc‐test and, if appropriate, a two‐way ANOVA was used. Unless otherwise stated, data are presented as means with SD values. A p‐value of <0.05 was considered statistically significant. The statistical software was GraphPad Prism version 8.0. Statistical analysis strategy of mass spectrometry lipidomic analysis can be found in Supporting Information.

### Ethics Statement

This article does not contain any studies with human participants. All animal experiments were performed with governmental approval according to the NIH guidelines for the care and use of laboratory animals. Both the STAIR criteria and the ARRIVE guidelines have been followed.

## Conflict of Interest

The authors declare no conflict of interest.

## Author Contributions

D.F. and T.R.D. contributed equally to this work. W.W., W.Q.X., S.S.J.L., D.F., L.T., I.G., Y.P., L.Z., Y.K., X.Z., and Z.H. performed the experiments. W.W., D.F., and T.R.D. designed the study. D.F., S.S.J.L., M.N., and C.K. performed the statistical analysis of the lipidomics. T.R.D., D.F., H.B.H., H.L., and M.B. provided financial support. W.W., D.F., S.S.J.L., W.Q.X., Y.P., E.K., L.T., D.M.H., H.B.H., S.H., A.P.W., S.T.G., H.L., M.B., and T.R.D. wrote the manuscript.

## Supporting information

Supporting Information

## Data Availability

The data that support the findings of this study are available in the supplementary material of this article.

## References

[1] a) D. Bano , P. Nicotera , Stroke 2007, 38, 674; 17261713 10.1161/01.STR.0000256294.46009.29

[2] a) J. Ye , Z. Jiang , X. Chen , M. Liu , J. Li , N. Liu , J. Neurochem. 2017, 142, 215; 28407242 10.1111/jnc.14042

[3] a) C. Iadecola , J. Anrather , Nat. Med. 2011, 17, 796; 21738161 10.1038/nm.2399 PMC3137275

[4] a) S. Heindl , B. Gesierich , C. Benakis , G. Llovera , M. Duering , A. Liesz , Front. Cell. Neurosci. 2018, 12, 106; 29725290 10.3389/fncel.2018.00106 PMC5917008

[5] A. Fernandes , L. Miller‐Fleming , T. F. Pais , Cell. Mol. Life Sci. 2014, 71, 3969. 25008043 10.1007/s00018-014-1670-8 PMC11113719

[6] a) C. Qin , L.‐Q. Zhou , X.‐T. Ma , Z.‐W. Hu , S. Yang , M. Chen , D. B. Bosco , L.‐J. Wu , D.‐S. Tian , Neurosci. Bull. 2019, 35, 921; 31062335 10.1007/s12264-019-00388-3 PMC6754485

[7] a) M. Arbaizar‐Rovirosa , M. Gallizioli , J. Pedragosa , J. J. Lozano , C. Casal , A. Pol , A. M. Planas , bioRxiv 2022; 10.15252/emmm.202217175 PMC9906381 36541061

[8] a) F. M. Feringa , R. Van der Kant , Front. Aging Neurosci. 2021, 13, 333; 10.3389/fnagi.2021.690372 PMC8264368 34248607

[9] J. Marschallinger , T. Iram , M. Zardeneta , S. E. Lee , B. Lehallier , M. S. Haney , J. V. Pluvinage , V. Mathur , O. Hahn , D. W. Morgens , Nat. Neurosci. 2020, 23, 194. 31959936 10.1038/s41593-019-0566-1 PMC7595134

[10] a) L. Liu , K. Zhang , H. Sandoval , S. Yamamoto , M. Jaiswal , E. Sanz , Z. Li , J. Hui , B. H. Graham , A. Quintana , Cell 2015, 160, 177; 25594180 10.1016/j.cell.2014.12.019 PMC4377295

[11] J. Anrather , C. Iadecola , Neurotherapeutics 2016, 13, 661. 27730544 10.1007/s13311-016-0483-x PMC5081118

[12] M. Arbaizar‐Rovirosa , J. Pedragosa , J. J. Lozano , C. Casal , A. Pol , M. Gallizioli , A. M. Planas , EMBO Mol. Med. 2022, 17175. 10.15252/emmm.202217175 PMC9906381 36541061

[13] a) S. Safaiyan , S. Besson‐Girard , T. Kaya , L. Cantuti‐Castelvetri , L. Liu , H. Ji , M. Schifferer , G. Gouna , F. Usifo , N. Kannaiyan , D. Fitzner , X. Xiang , M. J. Rossner , M. Brendel , O. Gokce , M. Simons , Neuron 2021, 109, 1100; 33606969 10.1016/j.neuron.2021.01.027

[14] M. Gallizioli , M. Arbaizar‐Rovirosa , D. Brea , A. M. Planas , Semin. Immunopathol. 2023, 45, 367. 37045990 10.1007/s00281-023-00990-8 PMC10279582

[15] a) N. Kayagaki , M. T. Wong , I. B. Stowe , S. R. Ramani , L. C. Gonzalez , S. Akashi‐Takamura , K. Miyake , J. Zhang , W. P. Lee , A. Muszyński , L. S. Forsberg , R. W. Carlson , V. M. Dixit , Science 2013, 341, 1246; 23887873 10.1126/science.1240248

[16] M. Zusso , V. Lunardi , D. Franceschini , A. Pagetta , R. Lo , S. Stifani , A. C. Frigo , P. Giusti , S. Moro , J. Neuroinflammation 2019, 16, 148. 31319868 10.1186/s12974-019-1538-9 PMC6637517

[17] a) C. Cheng , F. Geng , X. Cheng , D. Guo , Cancer Commun. 2018, 38, 1; 10.1186/s40880-018-0301-4 PMC5993136 29784041

[18] B. B. Madison , J. Lipid Res. 2016, 57, 333,. 26798145 10.1194/jlr.C066712 PMC4766982

[19] a) A. Kusnadi , S. H. Park , R. Yuan , T. Pannellini , E. Giannopoulou , D. Oliver , T. Lu , K. H. Park‐Min , L. B. Ivashkiv , Immunity 2019, 51, 241; 31303399 10.1016/j.immuni.2019.06.005 PMC6709581

[20] X. Chen , C. Chen , S. Fan , S. Wu , F. Yang , Z. Fang , H. Fu , Y. Li , J. Neuroinflammation 2018, 15, 1. 29678169 10.1186/s12974-018-1151-3 PMC5909267

[21] T. Y. Chang , C. C. Chang , N. Ohgami , Y. Yamauchi , Annu. Rev. Cell Dev. Biol. 2006, 22, 129. 16753029 10.1146/annurev.cellbio.22.010305.104656

[22] E. Y. Bryleva , M. A. Rogers , C. C. Chang , F. Buen , B. T. Harris , E. Rousselet , N. G. Seidah , S. Oddo , F. M. LaFerla , T. A. Spencer , W. F. Hickey , T. Y. Chang , Proc. Natl. Acad. Sci. USA 2010, 107, 3081. 20133765 10.1073/pnas.0913828107 PMC2840286

[23] J. Wang , W. He , J. Zhang , Heliyon 2023, 9, e14713. 37025898 10.1016/j.heliyon.2023.e14713 PMC10070543

[24] M. Huang , Y. Wan , L. Mao , Q. W. He , Y. P. Xia , M. Li , Y. N. Li , H. J. Jin , B. Hu , CNS Neurosci. Ther. 2017, 23, 222. 27991729 10.1111/cns.12665 PMC6492671

[25] E. R. Chernykh , E. Y. Shevela , N. M. Starostina , S. A. Morozov , M. N. Davydova , E. V. Menyaeva , A. A. Ostanin , Cell Transplant. 2016, 25, 1461. 26671426 10.3727/096368915X690279

[26] E. Vankriekelsvenne , U. Chrzanowski , K. Manzhula , T. Greiner , A. Wree , A. Hawlitschka , G. Llovera , J. Zhan , S. Joost , C. Schmitz , P. Ponsaerts , S. Amor , E. Nutma , M. Kipp , H. Kaddatz , Glia 2022, 70, 1170. 35246882 10.1002/glia.24164

[27] a) T. Zrzavy , S. Hametner , I. Wimmer , O. Butovsky , H. L. Weiner , H. Lassmann , Brain 2017, 140, 1900; 28541408 10.1093/brain/awx113 PMC6057548

[28] M. Schwabenland , W. Brück , J. Priller , C. Stadelmann , H. Lassmann , M. Prinz , Acta Neuropathol. 2021, 142, 923. 34623511 10.1007/s00401-021-02370-8 PMC8498770

[29] F. Yu , Y. Wang , A. R. Stetler , R. K. Leak , X. Hu , J. Chen , CNS Neurosci. Ther. 2022, 28, 1279. 35751629 10.1111/cns.13899 PMC9344092

[30] C. Perego , S. Fumagalli , M. G. De Simoni , J. Neuroinflammation 2011, 8, 174. 22152337 10.1186/1742-2094-8-174 PMC3251548

[31] S. J. Karlen , E. B. Miller , X. Wang , E. S. Levine , R. J. Zawadzki , M. E. Burns , J. Neuroinflammation 2018, 15, 344. 30553275 10.1186/s12974-018-1365-4 PMC7659426

[32] a) T. Li , S. Pang , Y. Yu , X. Wu , J. Guo , S. Zhang , Brain 2013, 136, 3578; 24154617 10.1093/brain/awt287

[33] C. Claes , E. P. Danhash , J. Hasselmann , J. P. Chadarevian , S. K. Shabestari , W. E. England , T. E. Lim , J. L. S. Hidalgo , R. C. Spitale , H. Davtyan , Mol. Neurodegener. 2021, 16, 50. 34301296 10.1186/s13024-021-00473-0 PMC8305935

[34] a) D. M. Boucher , V. Vijithakumar , M. Ouimet , Immunometabolism 2021, 3, e210021;

[35] a) Y. Hu , W. Mai , L. Chen , K. Cao , B. Zhang , Z. Zhang , Y. Liu , H. Lou , S. Duan , Z. Gao , Glia 2020, 68, 1031; 31793691 10.1002/glia.23760

[36] Q. Raas , F. E. Saih , C. Gondcaille , D. Trompier , Y. Hamon , V. Leoni , C. Caccia , B. Nasser , M. Jadot , F. Ménétrier , G. Lizard , M. Cherkaoui‐Malki , P. Andreoletti , S. Savary , Biochim. Biophys. Acta Mol. Cell Biol. Lipids 2019, 1864, 567. 30312667 10.1016/j.bbalip.2018.10.005

[37] D. A. Becktel , J. C. Zbesko , J. B. Frye , A. G. Chung , M. Hayes , K. Calderon , J. W. Grover , A. Li , F. G. Garcia , M. Tavera‐Garcia , J. Neurosci. 2022, 42, 325. 34819339 10.1523/JNEUROSCI.0933-21.2021 PMC8802936

[38] S. H. Loppi , M. A. Tavera‐Garcia , D. A. Becktel , B. K. Maiyo , K. E. Johnson , T. V. Nguyen , R. G. Schnellmann , K. P. Doyle , J. Cereb. Blood Flow Metab. 2023, 43, 1099. 36772984 10.1177/0271678X231157298 PMC10291449

[39] a) E. Jarc , T. Petan , Biochimie. 2020, 169, 69; 31786231 10.1016/j.biochi.2019.11.016

[40] F. Purroy , A. Ois , M. Jove , G. Arque , J. Sol , G. Mauri‐Capdevila , A. R. Campello , R. Pamplona , M. Portero , J. Roquer , Sci. Rep. 2023. 10.1038/s41598-023-40838-7 PMC10444771 37607967

[41] H. Lian , E. Roy , H. Zheng , Bio. Protoc. 2016, 6, e1989. 10.21769/BioProtoc.1989 PMC5669279 29104890

[42] T. Fath , Y. D. Ke , P. Gunning , J. Götz , L. M. Ittner , Nat. Protoc. 2009, 4, 78. 19131959 10.1038/nprot.2008.199

[43] T. R. Doeppner , J. Herz , A. Görgens , J. Schlechter , A.‐K. Ludwig , S. Radtke , K. de Miroschedji , P. A. Horn , B. Giebel , D. M. Hermann , Stem. Cells Transl. Med. 2015, 4, 1131. 26339036 10.5966/sctm.2015-0078 PMC4572905

[44] S. D. Skaper , L. Facci , Methods Mol. Bio. 2018, 1727, 49. 29222772 10.1007/978-1-4939-7571-6_4

[45] T. L. Riss , R. A. Moravec , A. L. Niles , S. Duellman , H. A. Benink , T. J. Worzella , L. Minor , Assay Guidance Manual 2016.

[46] M. A. Surma , M. J. Gerl , R. Herzog , J. Helppi , K. Simons , C. Klose , Sci. Rep. 2021, 11, 19364. 34588529 10.1038/s41598-021-98702-5 PMC8481471

[47] R. Herzog , K. Schuhmann , D. Schwudke , J. L. Sampaio , S. R. Bornstein , M. Schroeder , A. Shevchenko , PLoS One 2012, 7, e29851. 22272252 10.1371/journal.pone.0029851 PMC3260173

