# Supplementary material for: Dynamic Brain Lipid Profiles Modulate Microglial Lipid Droplet Accumulation and Inflammation Under Ischemic Conditions in Mice
Source: Adv Sci (Weinh). 2024 Sep 9;11(41):2306863. doi: 10.1002/advs.202306863 (PMC11538718; doi:10.1002/advs.202306863)
Supplement: Supplementary file 1 — Supporting Information [file ADVS-11-2306863-s001.docx]

**SUPPLEMENTARY MATERIALS**

**Dynamic brain lipid profiles modulate microglial lipid droplet accumulation and inflammation under ischemic conditions in mice**

Wei Wei^1, 2^, Seyed Siyawasch Justus Lattau^1^, Wenqiang Xin^1^, Yongli Pan^1^, Lars Tatenhorst^1^, Lin Zhang^1^, Irina Graf^1^, Yaoyun Kuang^1^, Xuan Zheng^1^, Zhongnan Hao^1^, Aurel Popa-Wagner^3^, Stefan T Gerner^4^, Sabine Huber^4^, Manuel Nietert^5^, Christian Klose^6^, Ertugrul Kilic^7^, Dirk M Hermann^3^, Mathias Bähr^1^, Hagen B Huttner^4^, Hua Liu^2^, Dirk Fitzner^1*#^, and Thorsten R Doeppner^1,4,8-10*#^

^1^Department of Neurology, University Medicine Göttingen (UMG), University of Göttingen, Göttingen, Germany

^2^Department of Neurology, the Affiliated Hospital of Southwest Jiaotong University & The Third People's Hospital of Chengdu, Chengdu, Sichuan, China

^3^Department of Neurology, University Hospital Essen, University of Duisburg-Essen, Essen, Germany

^4^Department of Neurology, University of Giessen Medical School, Giessen, Germany.

^5^Department of Medical Bioinformatics, UMG, University of Göttingen, Göttingen, Germany

^6^Lipotype GmbH, Dresden, Germany

^7^Department of Physiology, Faculty of Medicine, Istanbul Medeniyet University, Istanbul, Turkey

^8^Department of Anatomy and Cell Biology, Medical University of Varna, Varna, Bulgaria.

^9^Center for Mind, Brain and Behavior (CMBB), University of Marburg and Justus Liebig University Giessen, Germany

^10^Research Institute for Health Sciences and Technologies (SABITA), Medipol University, Istanbul, Turkey

# Theses authors contributed equally to this work

* Corresponding author

**Correspondence:**

**Thorsten R. Doeppner**, MD, MSc, MHBA

Department of Neurology

University of Giessen Medical School, Giessen, Germany

Phone: +49-641-98545393

Email: [thorsten.doeppner@neuro.med.uni-giessen.de](mailto:thorsten.doeppner@neuro.med.uni-giessen.de)

**Dirk Fitzner**, MD

Department of Neurology

University of Göttingen Medical School, Göttingen, Germany

Phone: +49-176-31222859

Email: [dirk.fitzner@med.uni-goettingen.de](mailto:dirk.fitzner@med.uni-goettingen.de)

**Supplementary Materials and Methods**

**Legal issues, animal housing, randomization and blinding**

All animal studies were conducted with local governmental approval according to the EU guidelines and regulations, following both ARRIVE and STAIR guidelines for the care and use of laboratory animals. Male C57BL/6J mice aged 10-12 weeks, (Charles River, Sulzfeld, Germany) were maintained in groups of 5 animals per cage on a regular 12 h light/12 h dark cycle. Animal surgery and sample collection were always performed in the morning throughout the study. At all stages of the studies, the allocation of animals to the experiments was randomized. The researchers with local animal experimental licenses performed animal surgery and sample collection and remained blinded during all phases of the study, whereas another investigator prepared the experimental solution and data collection. These solutions and groups were received and disclosed only after the study.

**Tissue sample harvest**

We used immunofluorescent analysis to investigate brain tissue samples at different time points after MCAO surgery. We found that LD-enriched microglia were mainly located in the ipsilateral hemisphere cortex region, i.e., in the lesion center. We defined the region as depicted in Supplementary Fig. S6, and then collected tissue samples of these regions from MCAO mice (sham, 3 d, 7 d and 28 d post-ischemia).

C57BL/6J mice with MCAO followed by different reperfusion time were anesthetized with ketamine/xylazine, then mouse brains were perfused with cold PBS with a micropump. The mouse brains were sliced with a slicer matrix at a thickness of 2 mm, and brain specimens were collected at designated locations using a sample puncture needle (1 mm diameter, Kai medical, 0197, Germany). The collected brain tissue samples were immediately snap-frozen in liquid nitrogen. Brain samples were stored at -80°C for later analysis.

**Lipid sample extraction**

The method of lipid sample extraction was based on the protocol of Bligh and Dyer method [1]. Small amounts of chloroform and methanol were used in the primary extraction step. After the tissue or cells were washed twice with cold PBS, samples were homogenized in ultrapure water with a homogenizer or sonicator (10 mg tissue or 1 x 10^6^ cells). The homogenized samples were mixed with 1 volume of chloroform and 2 volumes of methanol. After vortexing thoroughly, the samples were incubated at room temperature for 20 min. Thereafter, another 1 volume of chloroform and 1 volume of ultrapure water was added. The suspension was then mixed and vortexed thoroughly and incubated for an additional 20 min. Then the samples were followed by ultracentrifugation at 15,000 x g for 15 min and were completely separated into three layers. The upper aqueous layer was removed and the lower organic phase was transferred to a new tube. After air-drying at 50°C to remove chloroform, the samples were vacuumed for 30 min to remove trace organic solvent and finally stored at -20°C.

**Total cholesterol/cholesteryl ester and free fatty acid quantification**

Total cholesterol/cholesteryl ester and total free fatty acids (FFA) in tissue or cell samples were analyzed by commercially available cholesterol quantification kits (Abcam, ab65359, Germany) and free fatty acid assay kit (Abcam, ab65341, Germany). According to the manufacturer’s protocol, to get a standard curve, diluting 25 μL of standard solution in different proportions to prepare six sets of standard samples. Testing samples were divided into two reaction mixtures: with or without cholesterol esterase. Total cholesterol and free cholesterol were measured separately, and added to appropriate wells of a 96-well plate. For FFA quantification, fatty acid probe, Acyl-CoA synthetase (ACS), reagent and enzyme mix were mixed thoroughly as reaction buffer according to the commercial instruction. Samples were mixed with reaction buffer and incubated at 37°C for 60 min in the dark, then measured at Ex/Em = 535/587 nm with a fluorometric microplate reader, and normalizing with the standard curve.

**Analysis of phagocytosis**

The analysis of microglial phagocytosis refer to the report of Hu et al [2]. To acquire images of phagocytosis, microglia were seeded in 4-well chambers (4 ×10^4^ cells/well, Sarstedt, Germany) and incubated with various treatments for 24 h. Nile red fluorescent microspheres (Invitrogen, Germany) were dissolved in cell culture medium according to the instruction of supplier. Cells were incubated with or without microspheres for another 4 h. The cells were then washed two times with PBS before fixation and permeabilization, then incubated with specific primary and secondary antibodies. Nuclei were stained with DAPI. Immunofluorescence slides were photographed with Zeiss Axioplan 2 fluorescence microscope (Zeiss, Oberkochen, Germany) or confocal scanning laser microscope (Zeiss LSM 700).

**ROS stress analysis**

To measure reactive oxygen species (ROS) in microglia, cells were seeded on 4-well chambers at a density of 4 × 10^4^ cells/well and treated with LPS, triacsin C, IL-4, conditioned medium or vehicle solution for 24 h. Then CellROX Orange (1:500; Invitrogen, Germany) was added to the cell culture medium and incubated at 37°C for 1 hour. Cells were washed twice with PBS, and nuclei were stained with Hoechst 33342 (1:5000; Thermo Fisher, Germany). CellROX Orange Intensity was detected with Zeiss Axioplan 2 fluorescence microscope (Zeiss, Oberkochen, Germany).

**Statistical analysis of mass spectrometry lipidomic**

Statistical analysis were performed using R (v.4.2.2) and Prism 9 (v.9.5.0, GraphPad) . Before generating the clustered heatmap, the data were normalized. Hierarchical clustering was calculated by applying Euclidean distance and complete linkage by using the R package MetaboAnalystR. Prior to performing unsupervised PCA and supervised oPLS-DA, log transformation, centering (by subtracting the means), and scaling (by dividing by standard deviations) were applied to reduce the impact of extreme values and address the compositional nature of the data. Additionally, we applied a threshold of > 90% prevalence calculated for every lipid subspecies to only include lipids present in a large fraction of the study. The PCA was generated by stats::prcomp(). The main reason for using oPLS-DA instead of PLS-DA is its ability to separate predictive from non-predictive (orthogonal) variation. The oPLS-DA was generated by using ropls::opls(). In this model one predictive component and one or two orthogonal components were calculated. The model fit was verified by evaluation of R2, which describes the variation of lipid species explained by the model and the cumulative R2X value (R2X(cum)) representing the explained variation in the matrix of predictor variables. R2Y(Cum), which represents the explained variation in the response matrix modeled by the predictor matrix was also calculated. The prediction ability of the model is described by Q2 and is calculated using 7-fold cross-validation (Q2(cum)). After permutation of the prediction the expected drop of R2Y (pR2Y) and Q2(pQ2) is evaluated. The values of R2, R2X(cum), R2Y(Cum) and Q2(cum) for each used oPLS-DA are provided below. Two-group comparisons for calculating the Volcano plots were conducted using Welch's unequal variances t-test. (OPLS-DA analysis are shown in the **Supplementary Table S4**).

**References**

[1] C. Breil, M. Abert Vian, T. Zemb, W. Kunz, F. J. I. j. o. m. s. Chemat, **2017**, *18* (4), 708.

[2] X. Hu, P. Li, Y. Guo, H. Wang, R. K. Leak, S. Chen, Y. Gao, J. J. S. Chen, **2012**, *43* (11), 3063.

**Supplementary Figure S1**

**
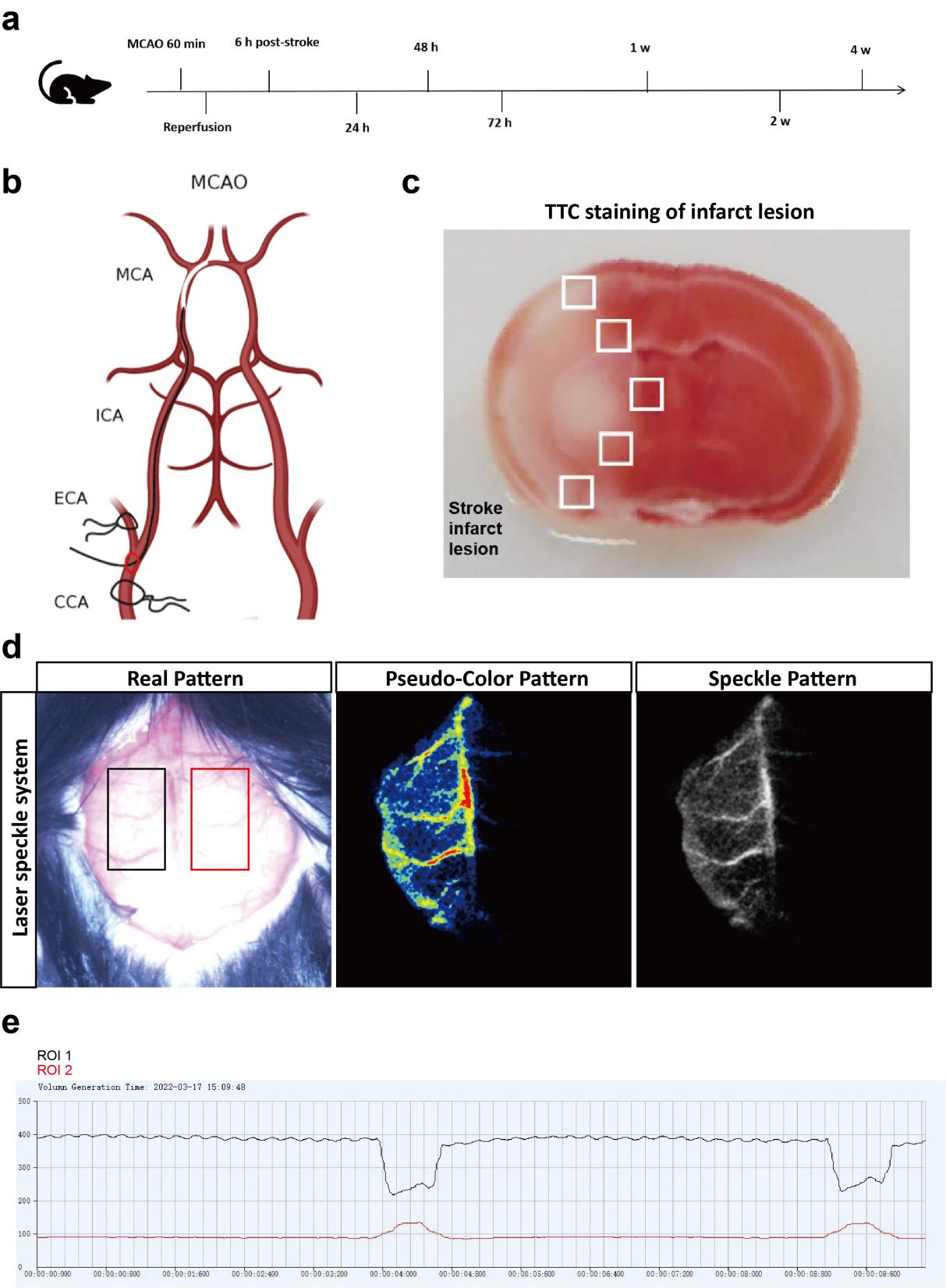
**

**S-Figure 1.** **Experimental paradigm, middle cerebral artery occlusion (MCAO), 2,3,5-Triphenyltetrazolium chloride (TTC) staining, and the laser speckle imaging system (LSIS).**

**a** Mice were sacrificed 1 day, 2 days, 3 days, 1 week, 2 weeks and 4 weeks after middle cerebral artery occlusion to extract brain tissue samples for subsequent experiments, respectively. **b** The surgical experimental paradigm of MCAO. **c** The infarcted region was measured with TTC staining after MCAO surgery (24 h). **d** The Real Pattern, Pseudo-Color Pattern, and Speckle Pattern images of the mouse skull indicated that the blood flow of the brain was blocked successfully. **e** The blood flow parameter map showed that the blood flow in the ROI 1 region (contralateral non-infarct side, black) was significantly higher than that in the ROI 2 region (ipsilateral infarct side, red). Abbreviation: TTC, 2,3,5-Triphenyltetrazolium chloride; MCAO, middle cerebral artery occlusion; ICA, internal carotid artery; ECA, external carotid artery; CCA, common carotid artery; MCA, middle cerebral artery; LSIS, laser speckle imaging system.

**Supplementary Figure S2**


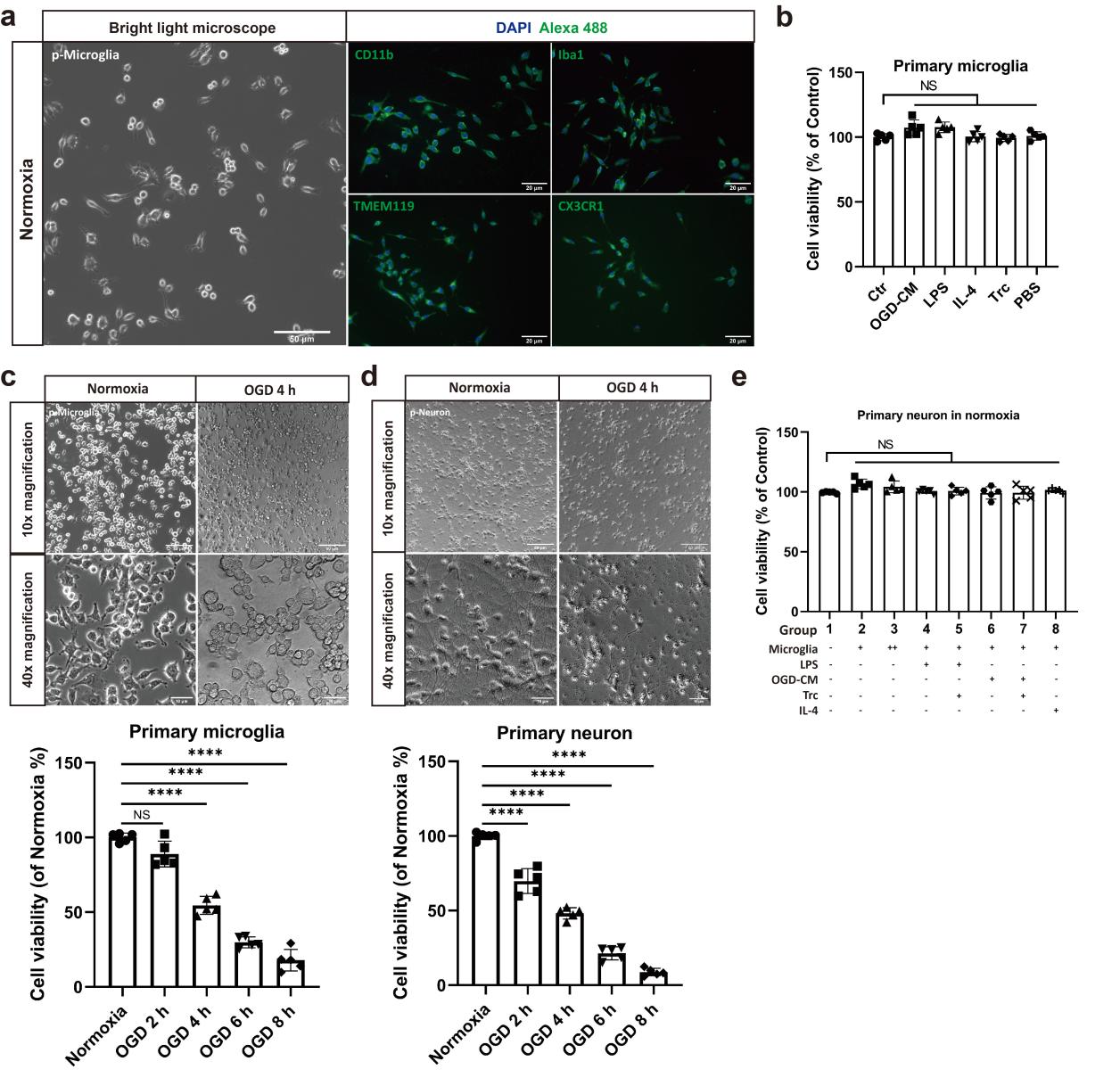


**S-Figure 2. Extraction and characterization of primary microglia/neurons and cell survival rate after OGD/RO in vitro.**

**a** Phase-contrast images of primary microglia under bright-field microscopy, as well as immunofluorescence stainings against the markers CD11b, Iba1, TMEM119, and CX3CR1, are shown. **b** Quantitative measurement of primary microglia survival rate in different treatment groups under normoxia condition by MTT assay (n = 5). **c** Primary microglia morphology under normoxia and hypoxia condition. Quantitative measurement of cell survival rate by MTT assay, microglia cultivated under standard cell culture conditions (Normoxia) served as control (n = 5). **d** Primary neuron morphology under normoxia and hypoxia condition. Quantitative measurement of cell survival rate by MTT assay, neurons cultivated under standard cell culture conditions (Normoxia) served as control (n = 5). **e** Quantitative measurement of survival rate of primary neuron co-cultured with different microglia under normoxia by MTT assay (n = 5). Statistical tests: One-way ANOVA followed by Tukey’s post-hoc-tests were used (**b-d, e**). Data are expressed as mean ± SD, ****p < 0.0001. Scale bars, 50 μm and 20 μm in (**a**), 50 μm and 10 μm in (**c, e**). Abbreviation: OGD, oxygen glucose deprivation; RO, reoxygenation; OGD-CM, conditioned medium from primary neuron after OGD; DAPI, 4′,6-diamidino-2-phenylindole; TMEM119, transmembrane protein 119; CX3CR1, C-X3-C motif chemokine receptor 1; MTT, 3-(4,5-dimethylthiazol-2-yl)-2,5-diphenyl tetrazolium bromide assay; Trc, triacsin C; LPS, lipopolysaccharide; IL-4, interleukin 4.

**Supplementary Figure S3**

**
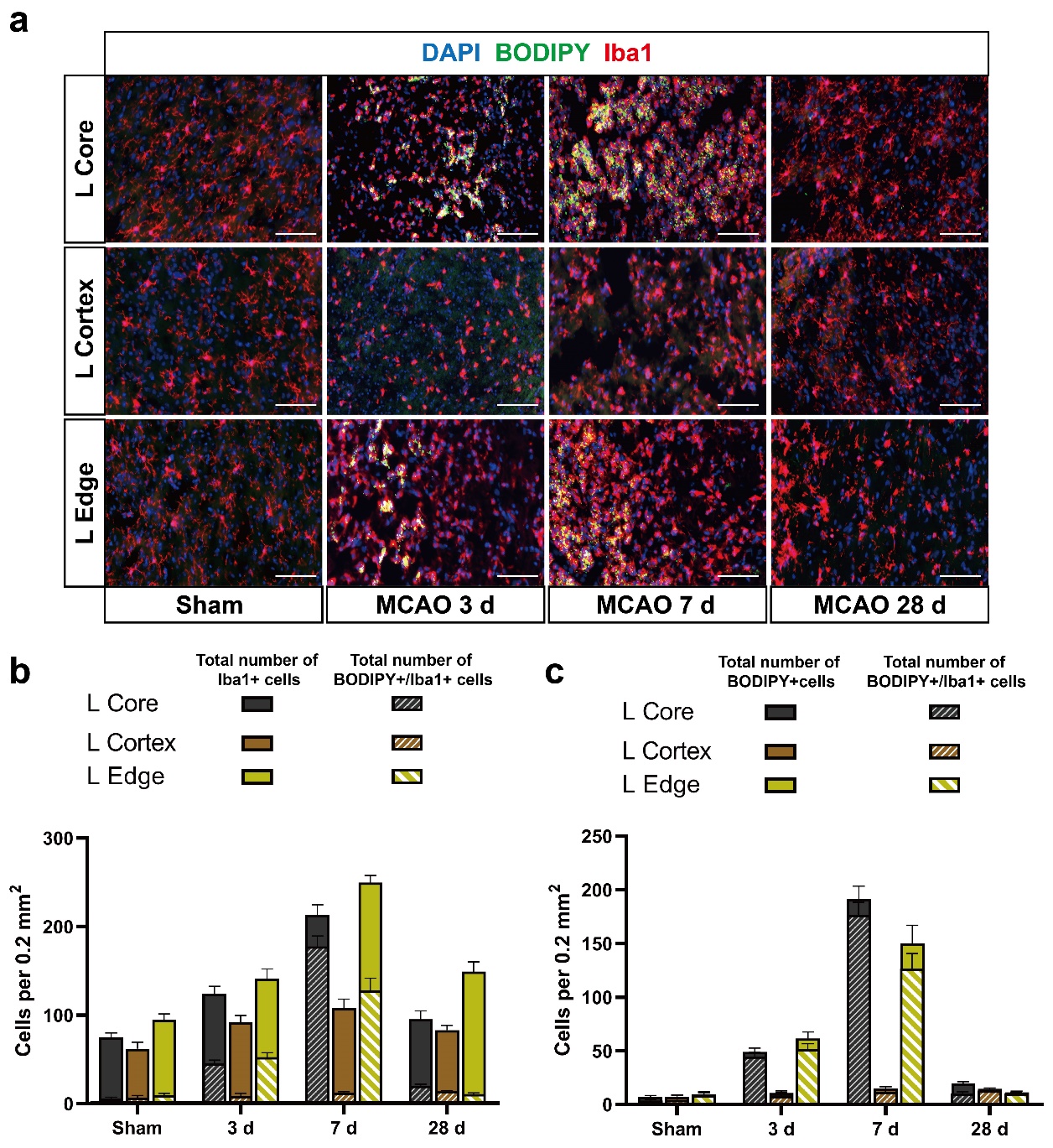
**

**S-Figure 3. Lipid droplet-rich microglia in different regions after MCAO.**

**a** Immunofluorescence staining of microglia (Iba1, red) and lipid droplets marker (BODIPY, green) for sham, post-ischemia day 3, day 7, and day 28 in the aforementioned regions. **b** Quantification of the cell numbers of Iba1+ and Iba1+/BODIPY+ in aforementioned regions. **c** Quantification of the cell numbers of BODIPY+ and Iba1+/BODIPY+ in aforementioned regions. Scale bars, 50 μm in (**a**). Abbreviation: MCAO, middle cerebral artery occlusion; LDs, lipid droplets.

**Supplementary Figure S4**

**
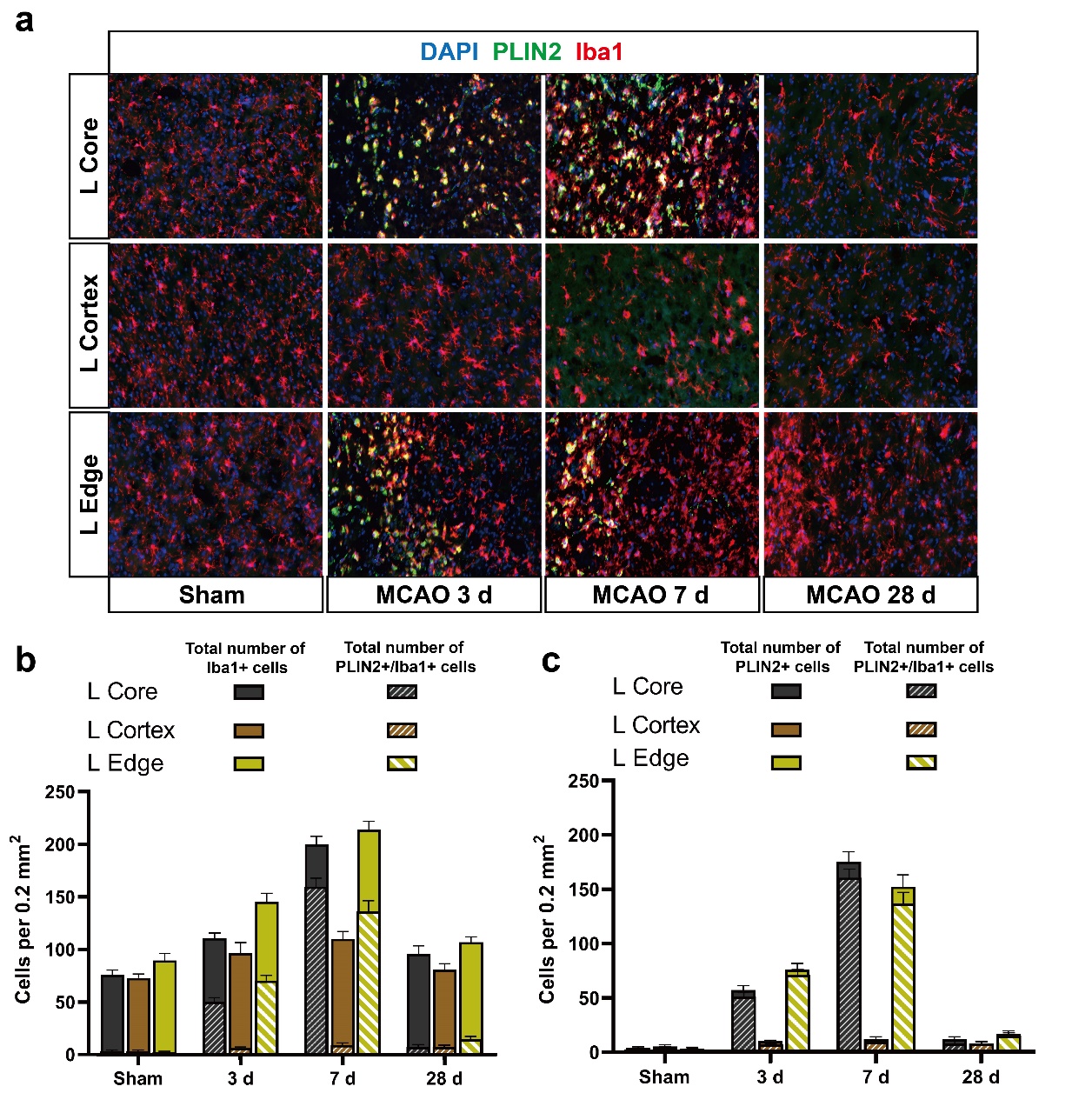
**

**S-Figure 4. PLIN2 expression in different regions after MCAO.**

**a** Immunofluorescence staining of microglia (Iba1, red) and PLIN2 for LDs marker (PLIN2, green) for sham, post-ischemia day 3, day 7, and day 28 in the aforementioned regions. **b** Quantification of the cell numbers of Iba1+ and Iba1+/PLIN2+ in aforementioned regions. **c** Quantification of the cell numbers of PLIN2+ and Iba1+/PLIN2+ in aforementioned regions. Scale bars, 50 μm in (**a**). Abbreviation: MCAO, middle cerebral artery occlusion; LDs, lipid droplets.

**Supplementary Figure S5**

**
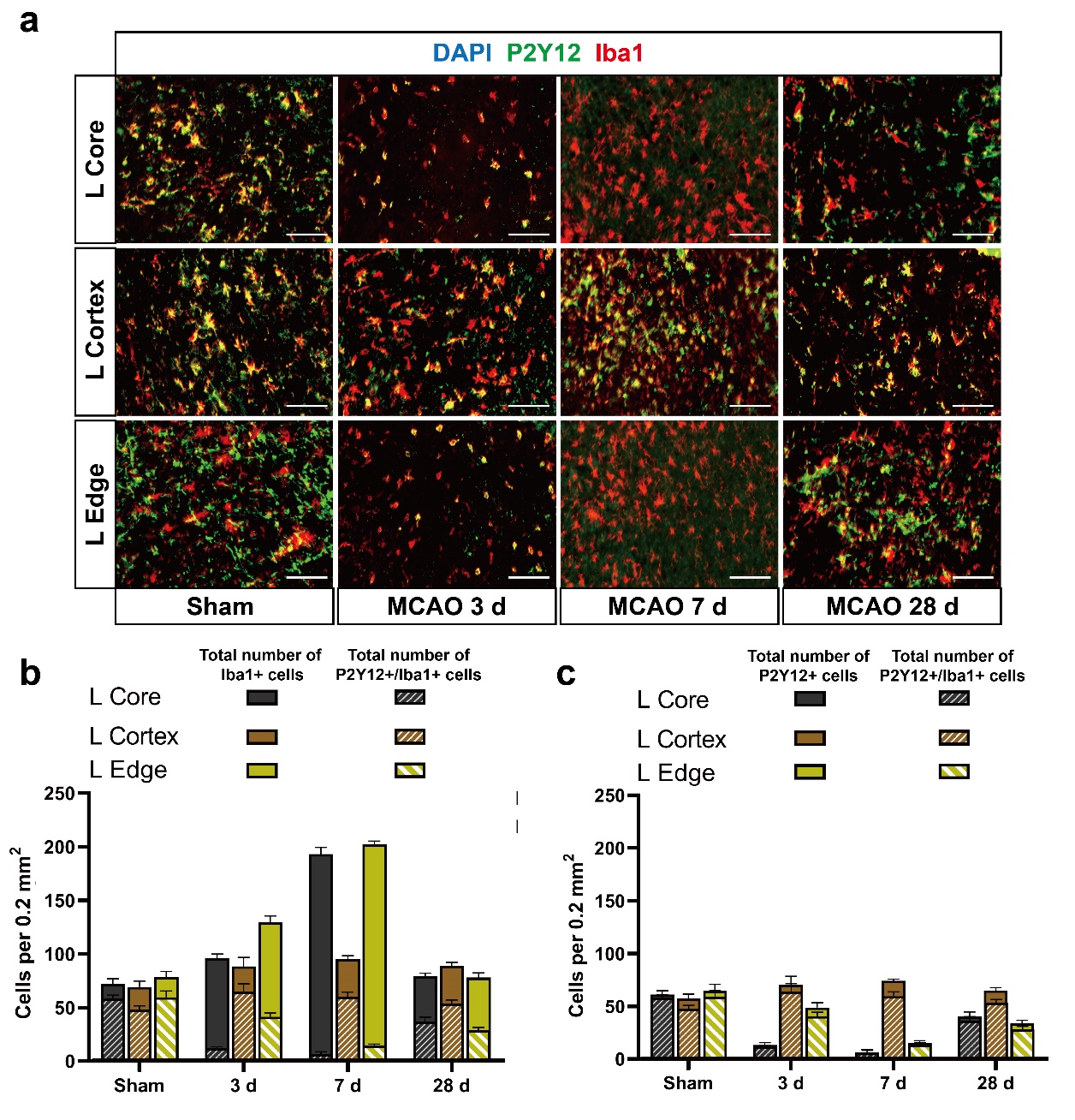
**

**S-Figure 5. Resting microglia marked by P2Y12 in different regions after MCAO.**

**a** Immunofluorescence staining of total microglia (Iba1, red) and resting microglia (P2Y12, green) for sham, post-ischemia day 3, day 7, and day 28 in the aforementioned regions. **b** Quantification of the cell numbers of Iba1+ and Iba1+/P2Y12+ in aforementioned regions. **c** Quantification of the cell numbers of P2Y12+ and Iba1+/ P2Y12+ in aforementioned regions. Scale bars, 50 μm in (**a**). Abbreviation: MCAO, middle cerebral artery occlusion; LDs, lipid droplets.

**Supplementary Figure S6**

**
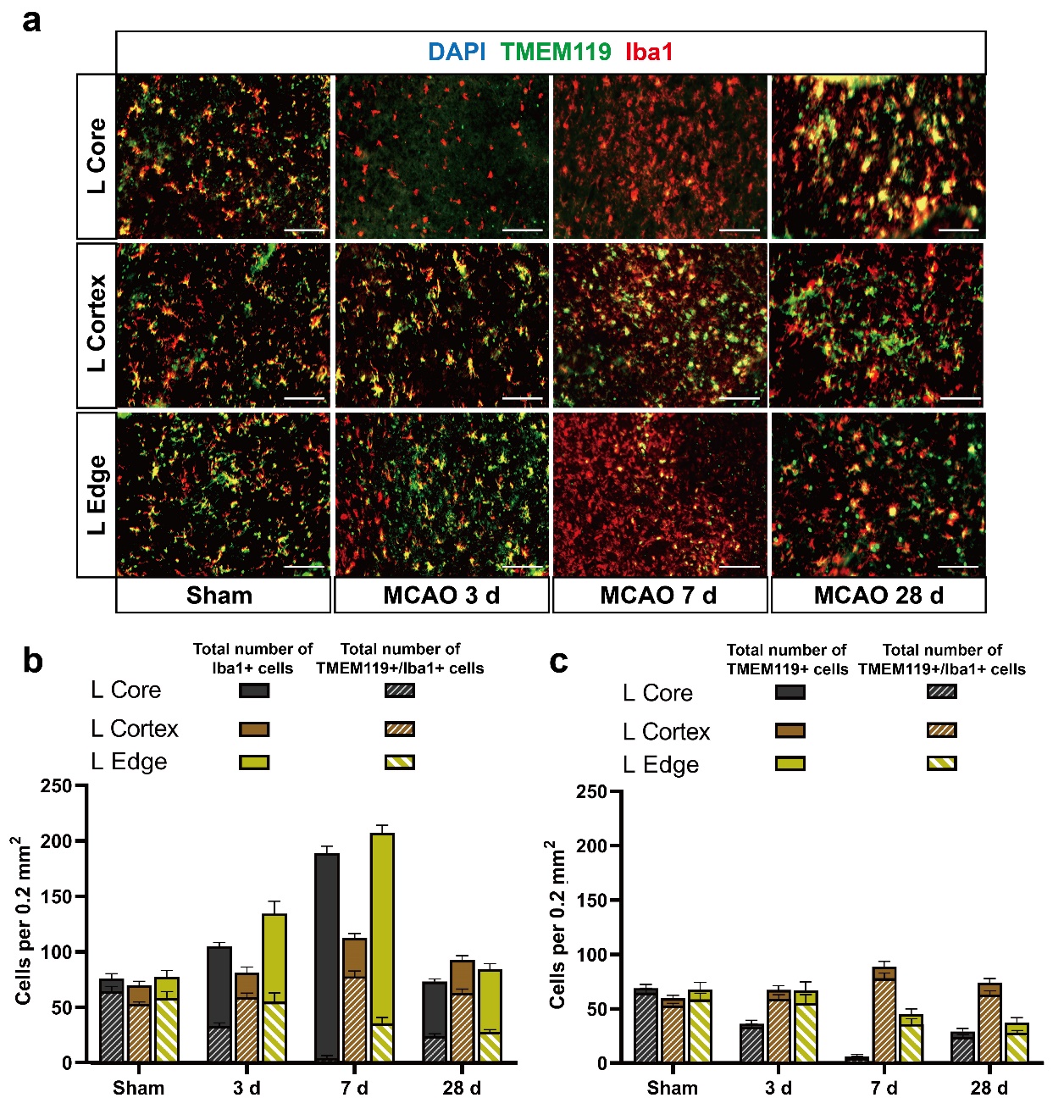
**

**S-Figure 6. Resting microglia marked by TMEM119 in different regions after MCAO.**

**a** Immunofluorescence staining of total microglia (Iba1, red) and resting microglia (TMEM119, green) for sham, post-ischemia day 3, day 7, and day 28 in the aforementioned regions. **b** Quantification of the cell numbers of Iba1+ and Iba1+/ TMEM119+ in aforementioned regions. **c** Quantification of the cell numbers of TMEM119+ and Iba1+/ TMEM119+ in aforementioned regions. Scale bars, 50 μm in (**a**). Abbreviation: MCAO, middle cerebral artery occlusion; LDs, lipid droplets.

**Supplementary Figure S7**


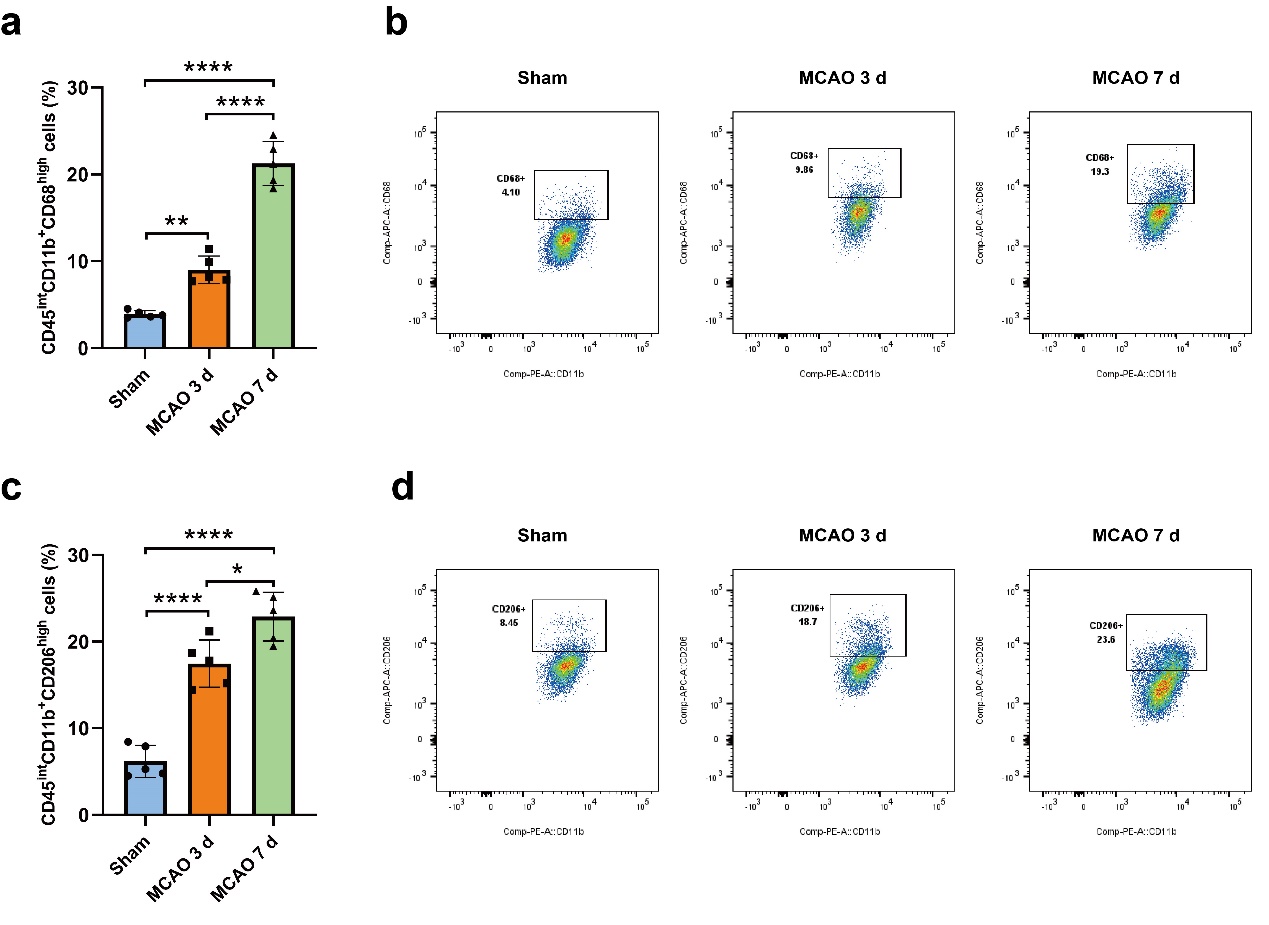


**S-Figure 7. Density plots of flow cytometry analysis for CD68+/CD206+ microglia.**

**a** Quantitative analysis of CD45intCD11b+CD68+ microglia in 3 groups (sham, 3 days after MCAO, 7 days after MCAO) by flow cytometry (n = 5). **b** Density plots of FACS showed a significant increase of inflammatory microglia (CD45intCD11b+CD68+) within post-ischemic 7 days. **c** Quantitative analysis of CD45intCD11b+CD206+ microglia in 3 groups (sham, 3 days after MCAO, 7 days after MCAO) by flow cytometry (n = 5). **d** Density plots of FACS showed a significant increase of inflammatory microglia (CD45intCD11b+CD68+) within post-ischemic 7 days. Abbreviation: MCAO, middle cerebral artery occlusion; LD, lipid droplet; FACS, fluorescence-activated cell sorting assay.

**Supplementary Figure S8**


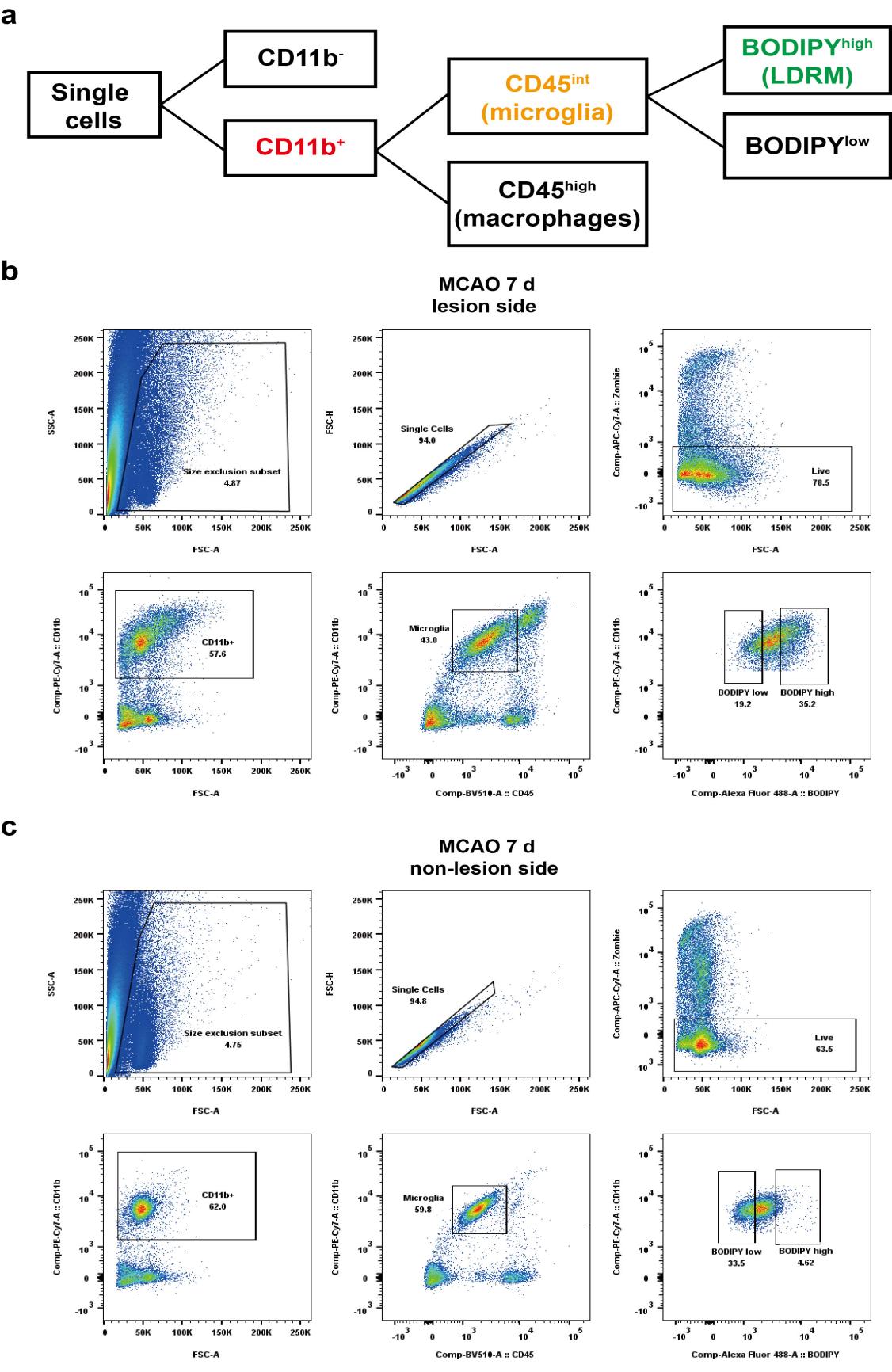


**S-Figure 8. Gating strategy and density plots of flow cytometry analysis.**

**a** Gating strategy for flow cytometry analysis. The subset of lipid droplets-rich microglia (CD11b+CD45^int^BODIPY+) was analyzed. **b, c** Representative flow cytometry measurement of the ischemic hemisphere (**b**) and non-ischemic hemisphere (**c**) in post-ischemic day 7 mice. All groups were exposed to 60 min of MCAO followed by 7 days of survival. For analysis, the cells were stained with antibodies against CD45, CD11b and BODIPY. Abbreviation: MCAO, middle cerebral artery occlusion; LD, lipid droplet; FACS, fluorescence-activated cell sorting assay; LDRM, lipid droplet-rich microglia.

**Supplementary Figure S9**


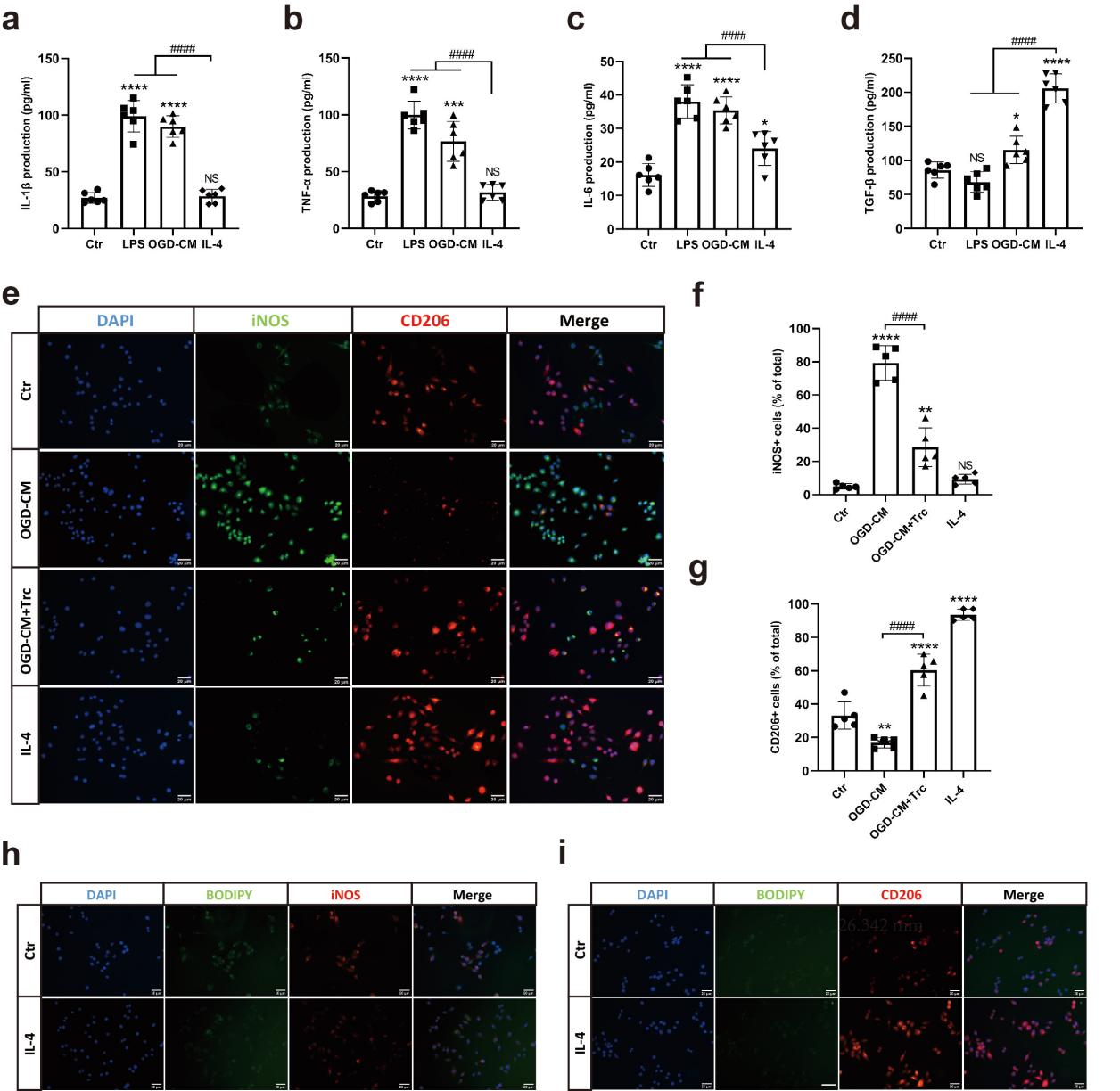


**S-Figure 9. Inflammation and phenotypic polarization of primary microglia.**

**a-d** ELISA analysis of cytokine levels of IL-1β (**a**), IL-6 (**b**), TNF-α (**c**), and TGF-β (**d**) in microglia under different conditions. (n = 6). **e** Immunofluorescence staining of M1 (iNOS, green) and M2 (CD206, red) polarization of primary microglia cells in the four groups. **f** Quantitative analysis of M1 polarization of microglia cells by proportion of iNOS+ cells to total cells in the aforementioned four groups (n = 5). **g** Quantitative analysis of M2 polarization of microglia cells by proportion of CD206+ cells to total cells in the aforementioned four groups (n = 5). **h** Immunofluorescence staining of LDs (BODIPY, green) and M1 (iNOS, red) polarization of primary microglia cells in control and IL-4 group. **i** Immunofluorescence staining of LDs (BODIPY, green) and M2 (CD206, red) polarization of primary microglia cells in control and IL-4 group. Statistical tests: One-way ANOVA followed by Tukey’s post-hoc-tests were used (**a-d, f, g**). Data are expressed as mean ± SD, NS: no significance, *p < 0.05, **p < 0.01, ***p < 0.001, ****p < 0.0001, and ^####^p<0.0001. Scale bars, 20 μm in (**e, h, i**). Abbreviation: LDs, lipid droplets; OGD, oxygen-glucose deprivation; LDRM, lipid droplet-rich microglia; OGD-CM, conditioned medium from primary neuron after OGD; Trc, triacsin C; DAPI, 4',6-Diamidin-2-phenylindol; LPS, lipopolysaccharide; IL-4, interleukin 4.

**Supplementary Figure S10**

**
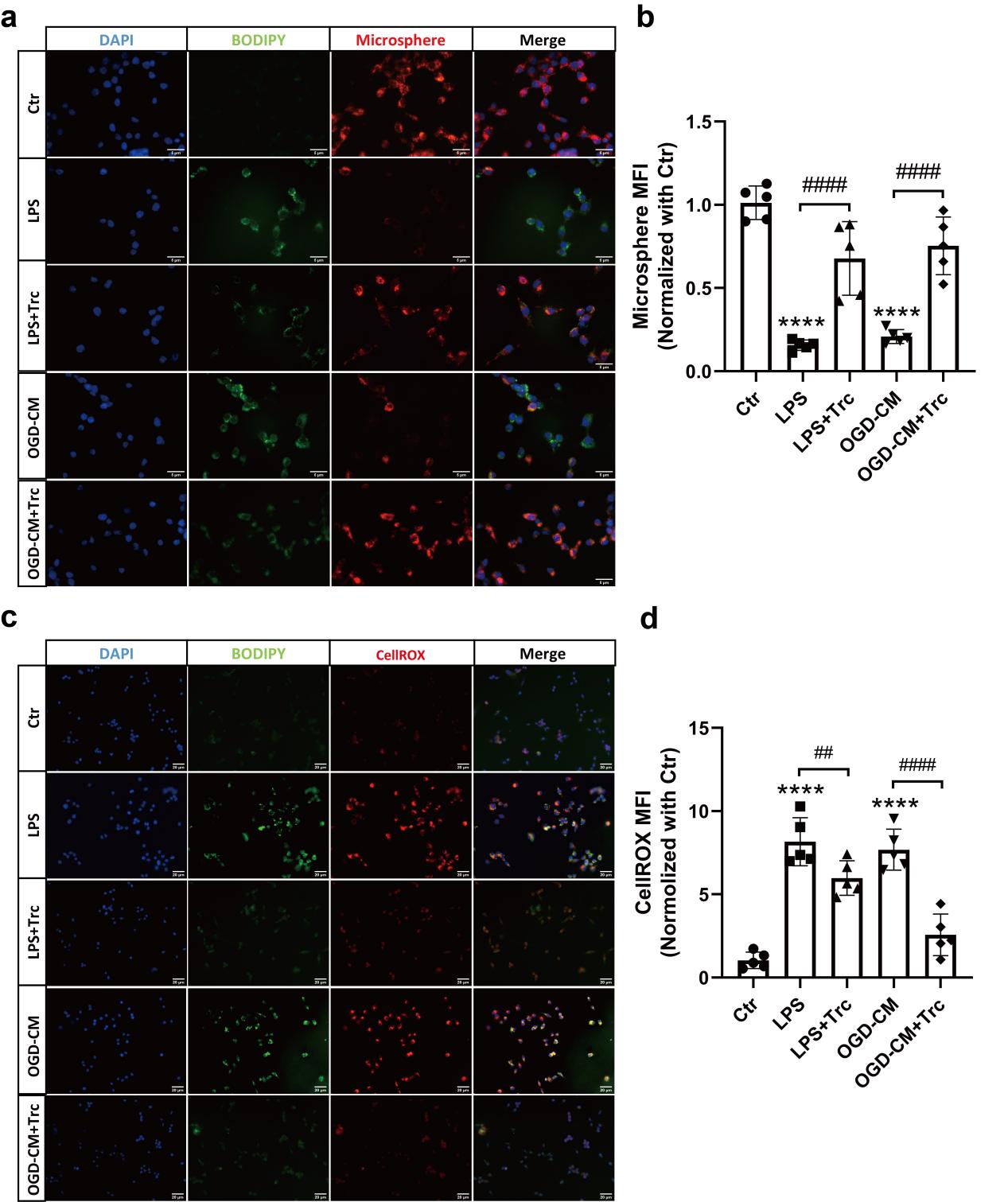
**

1. **Figure 10.** **LD-rich microglia with high cholesteryl ester levels display higher levels of ROS and impaired phagocytotic activity.**

**a** Immunofluorescence staining of BODIPY (green) and Microsphere (red) in microglia. **b** Quantification of Microsphere mean fluorescence intensity (MFI) in microglia. (n = 5). **c** Representative images of BODIPY (green) and CellROX (red) staining in microglia. **d** Quantification of CellROX MFI in microglia. Statistical tests: One-way ANOVA followed by Tukey’s post-hoc-tests were used (**b and d**). Data are expressed as mean ± SD, NS: no significance, *p < 0.05, **p < 0.01, ****p < 0.0001, ^##^p<0.01, and ^####^p<0.0001. Scale bars, 5 μm in (**a**) and 20 μm in (**c**). Abbreviation: LDs, lipid droplets; OGD, oxygen-glucose deprivation; OGD-CM, conditioned medium from primary neuron after OGD; Ctr, control group; DAPI, 4',6-Diamidin-2-phenylindol; LPS, lipopolysaccharide; MFI, mean fluorescence intensity; Trc, triacsin C.

**Supplementary Figure S11**

**
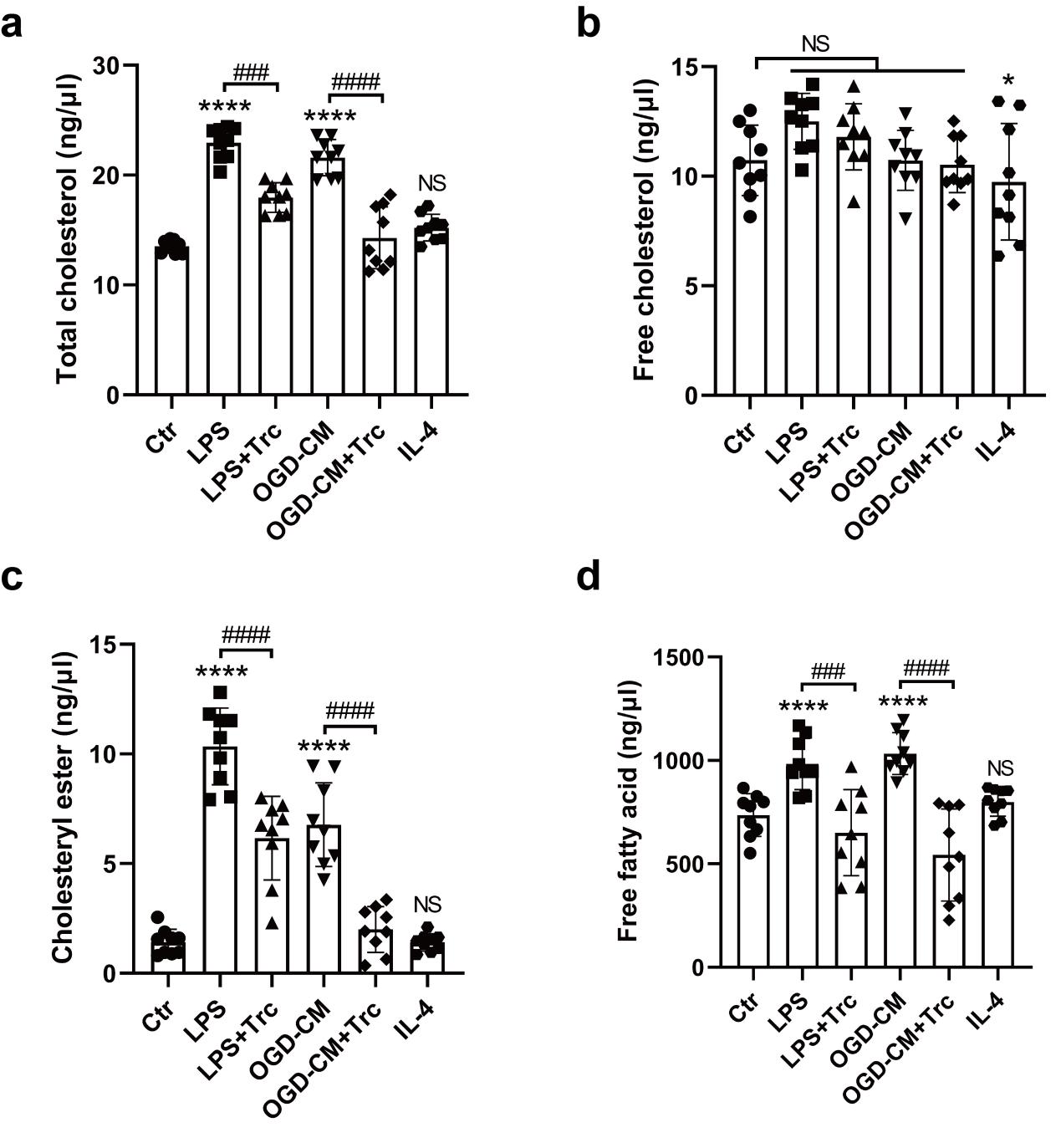
**

**S-Figure 11.** **LD-rich microglia with high cholesteryl ester levels display higher levels of ROS and impaired phagocytotic activity.**

**a** Quantification of the level of total cholesterol in different treated microglia (n = 9). **b** Quantification of the level of free cholesterol in different treated microglia (n = 9). **c** Quantification of the level of cholesteryl ester in different treated microglia (n = 9). **d** Quantification of the level of free fatty acid in different treated microglia (n = 9). Statistical tests: One-way ANOVA followed by Tukey’s post-hoc-tests were used (**a-d**). Data are expressed as mean ± SD, NS: no significance, *p < 0.05, **p < 0.01, ****p < 0.0001, ^###^p<0.001, and ^####^p<0.0001. Abbreviation: OGD, oxygen-glucose deprivation; OGD-CM, conditioned medium from primary neuron after OGD; Ctr, control group; LPS, lipopolysaccharide; IL-4, interleukin 4; Trc, triacsin C.

**Supplementary Figure S12**

**
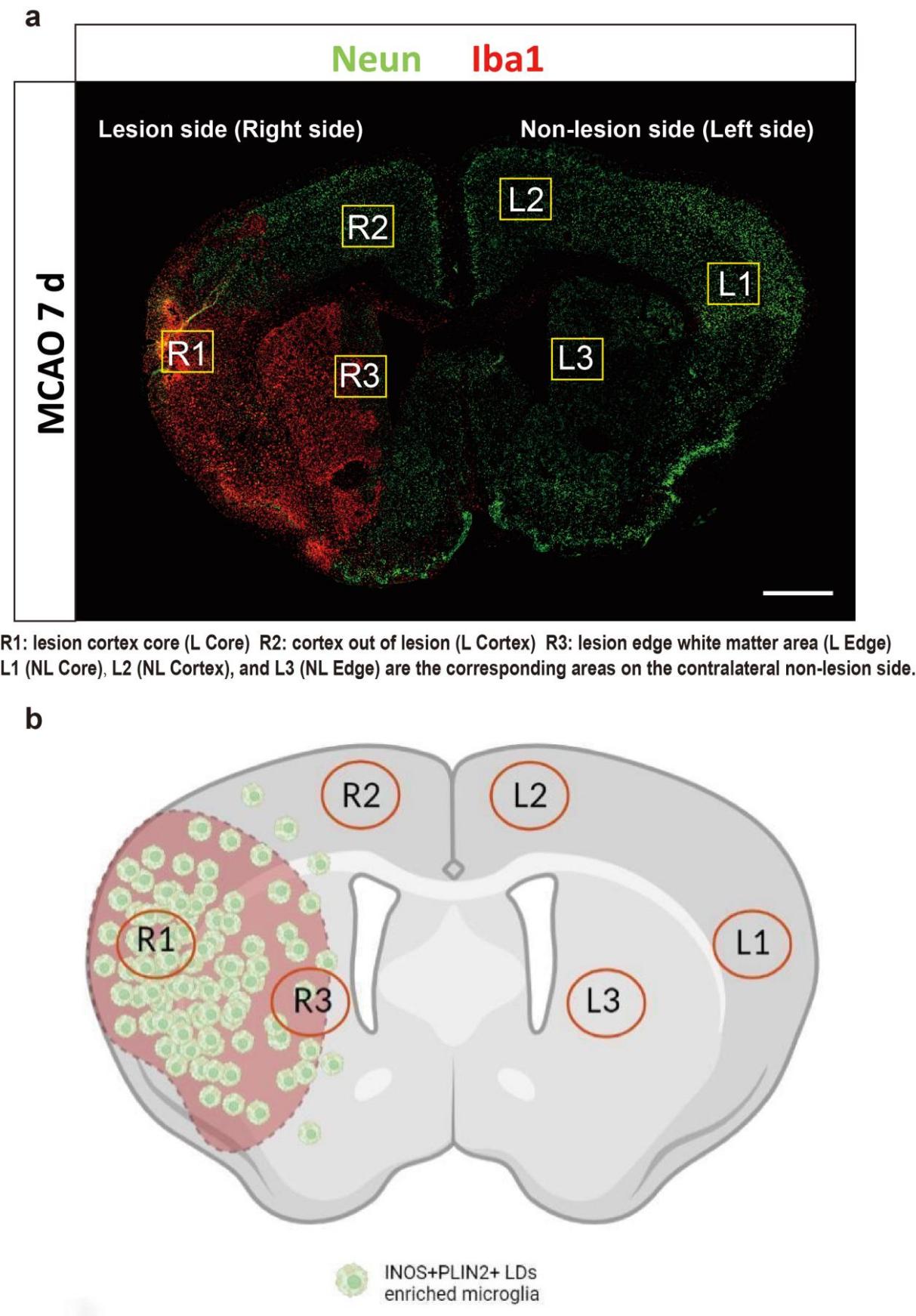
**

**S-Figure 12. Definition of brain regions after MCAO.**

**a** Post-ischemia day 7 whole brain immunofluorescence co-staining (Neuron, Neun, green and microglia, Iba1, red) were used to define the different regions of brain. Brain hemispheres were divided into different areas: R1 (lesion core of cortex, L Core), R2 (ipsilateral cortex out of lesion, L Cortex), R3 (white matter area of lesion edge, L Edge). L1 (NL Core), L2 (NL Cortex), and L3 (NL Edge) were the corresponding areas on the contralateral non-lesion side. **b** Schematic diagram of the divisions of the post-ischemic LDRM. The majority of iNOS+ PLIN2+ LDRM were found in 7 day L Core. Abbreviation: MCAO, middle cerebral artery occlusion; LDs, lipid droplets; PLIN2, perilipin 2; L Core, lesion core of cortex; L Cortex, ipsilateral cortex out of lesion; L Edge, white matter area of lesion edge; NL Core, contralateral non-lesion core of cortex; NL Cortex, contralateral non-lesion cortex out of lesion; NL Edge, white matter area of contralateral non-lesion edge.

**Supplementary Figure S13**


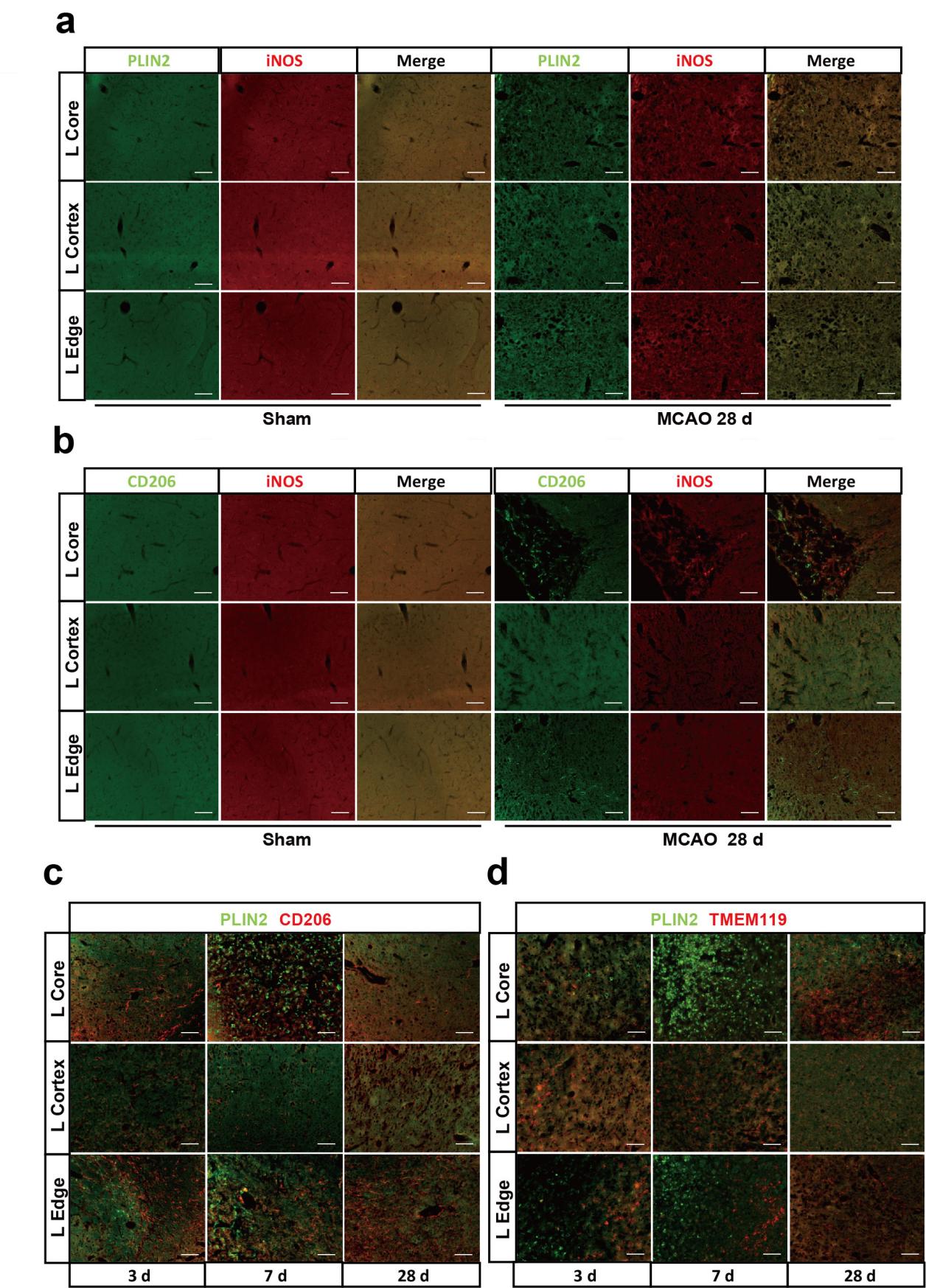


**S-Figure 13.** **Polarization patterns of microglia in different regions after MCAO.**

**a** Immunofluorescence staining of M1 microglia (iNOS, red) and lipid droplets surface protein marker (PLIN2, green) for sham and post-ischemia day 28 in the aforementioned six regions. **b** Immunofluorescence staining of polarization of M1 phenotype microglia (iNOS, red) and M2 phenotype microglia (CD206, green) for Sham and post-ischemia day 28 in the aforementioned six regions. **c** Immunofluorescence staining of PLIN2 (green) and CD206 (red) from post-ischemia day 3 to day 28. **d** Immunofluorescence staining of PLIN2 (green) and TMEM119 (red) from post-ischemia day 3 to day 28. Scale bars, 50 μm (**a-d**). Abbreviation: MCAO, middle cerebral artery occlusion; LDs, lipid droplets; MFI, mean fluorescence intensity; PLIN2, perilipin 2; L Core, lesion core of cortex; L Cortex, ipsilateral cortex out of lesion; L Edge, white matter area of lesion edge.

**Supplementary Figure S14**

**
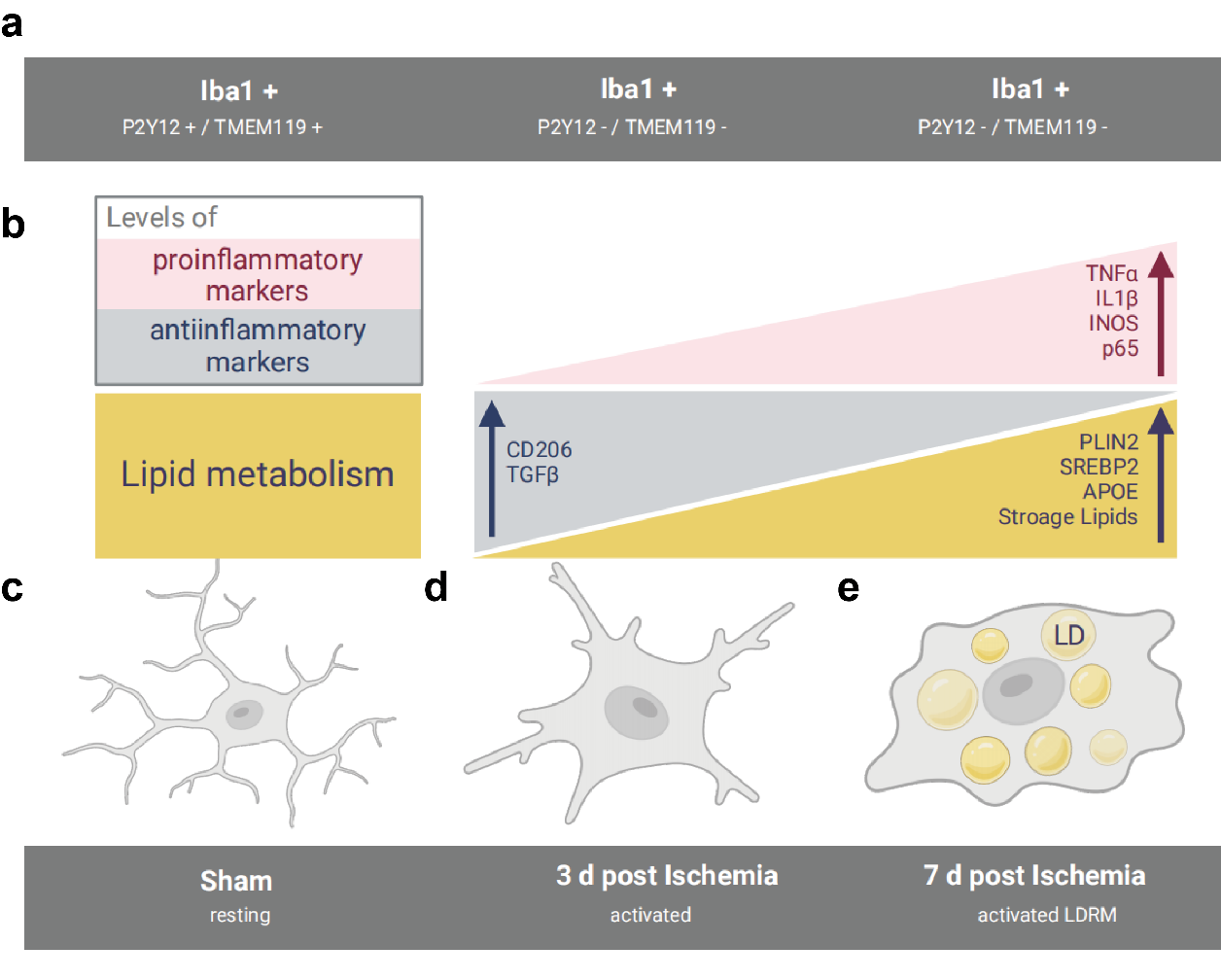
**

**S-Figure 14.** **Temporal dynamics of microglial activation states in correlation with lipid metabolism alterations within the core region of the ischemic lesion.**

**a** Resting microglial markers (P2Y12 and TMEM119) were positive in the lesion core of sham animals and turned negative 3 d post-ischemia. **b** Dynamic changes in levels of pro- and anti-inflammatory markers in correlation with alterations of the intralesional lipid metabolism over time. **c-e** Microglial activation states in sham, 3 d and 7 d post-ischemia. Abbreviation: PLIN2, perilipin 2; SREBP2, Sterol regulatory element-binding protein 2; APOE, apolipoprotein E; LD, lipid droplet; LDRM, lipid droplet-rich microglia.

**Supplementary Figure S15**

**
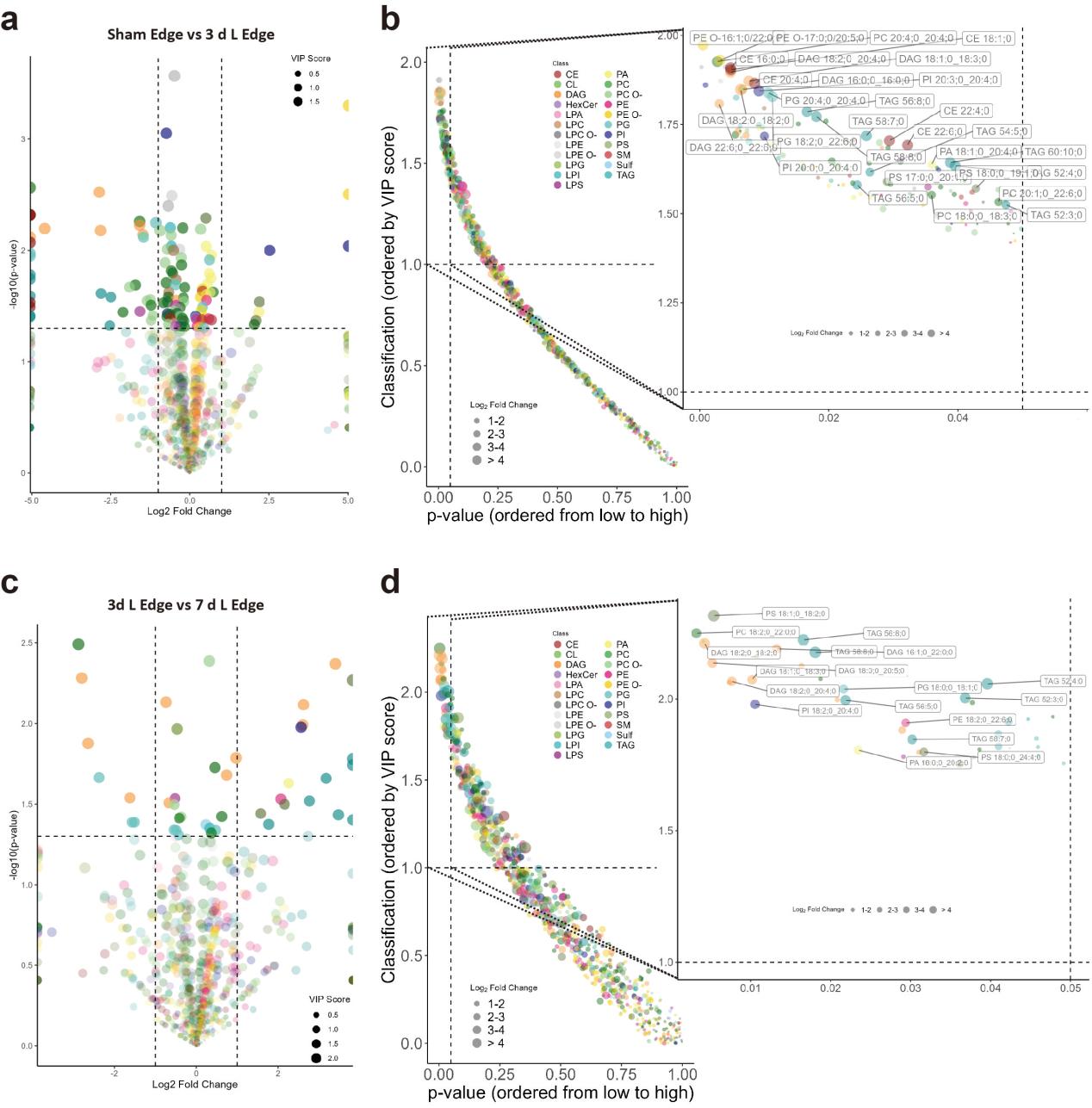
**

**S-Figure 15. Lipid species composition of mouse brain edge regions after stroke.**

**a** Volcano plot of lipid profiles in Sham Edge and 3 d L Edge. **b** VIP score from OPLS-DA combined with p-value from Volcano plot to identify lipid profiles that distinguish between Sham Edge and 3 d L Edge. **c** Volcano plot of lipid profiles in 3 d L Edge and 7 d L Edge. **d** VIP score from OPLS-DA combined with p-value from Volcano plot to identify lipid profiles that distinguish between 3 d L Core and 7 d L Core. Source data are provided as a Source Data file. Abbreviation: PCA, principal component analysis; OPLS-DA, orthogonal partial least squares discriminant analysis; L Edge, white matter area of lesion edge; CE, cholesteryl ester; Cer, ceramide; CL, cardiolipin; SM, sphingomyelin; DAG, diacylglycerol; TAG, triacylglycerol; LPA, lyso-phosphatidate; LPC, lyso-phosphatidyl-choline; LPC O-, ether-linked lyso-phosphatidyl-choline; LPE, lyso-phosphatidyl-ethanolamine; LPE O-, ether-linked lyso-phosphatidyl-ethanolamine; LPI, lyso-phosphatidyl-inositol; LPG, lyso-phosphatidyl-glycerol; LPS, lyso-phosphatidyl-serine; Sulf, sulfatide; HexCer, hexosylceramide; PA, phosphatidate; PC, phosphatidylcholine; PC O-, ether-linked phosphatidyl-choline; PE, phosphatidylethanolamine; PE O-, ether-linked phosphatidyl-ethanolamine; PS, phosphatidyl-serine; PI, phosphatidylinositol; PG, phosphatidylglycerol.

**Supplementary Figure S16**

**
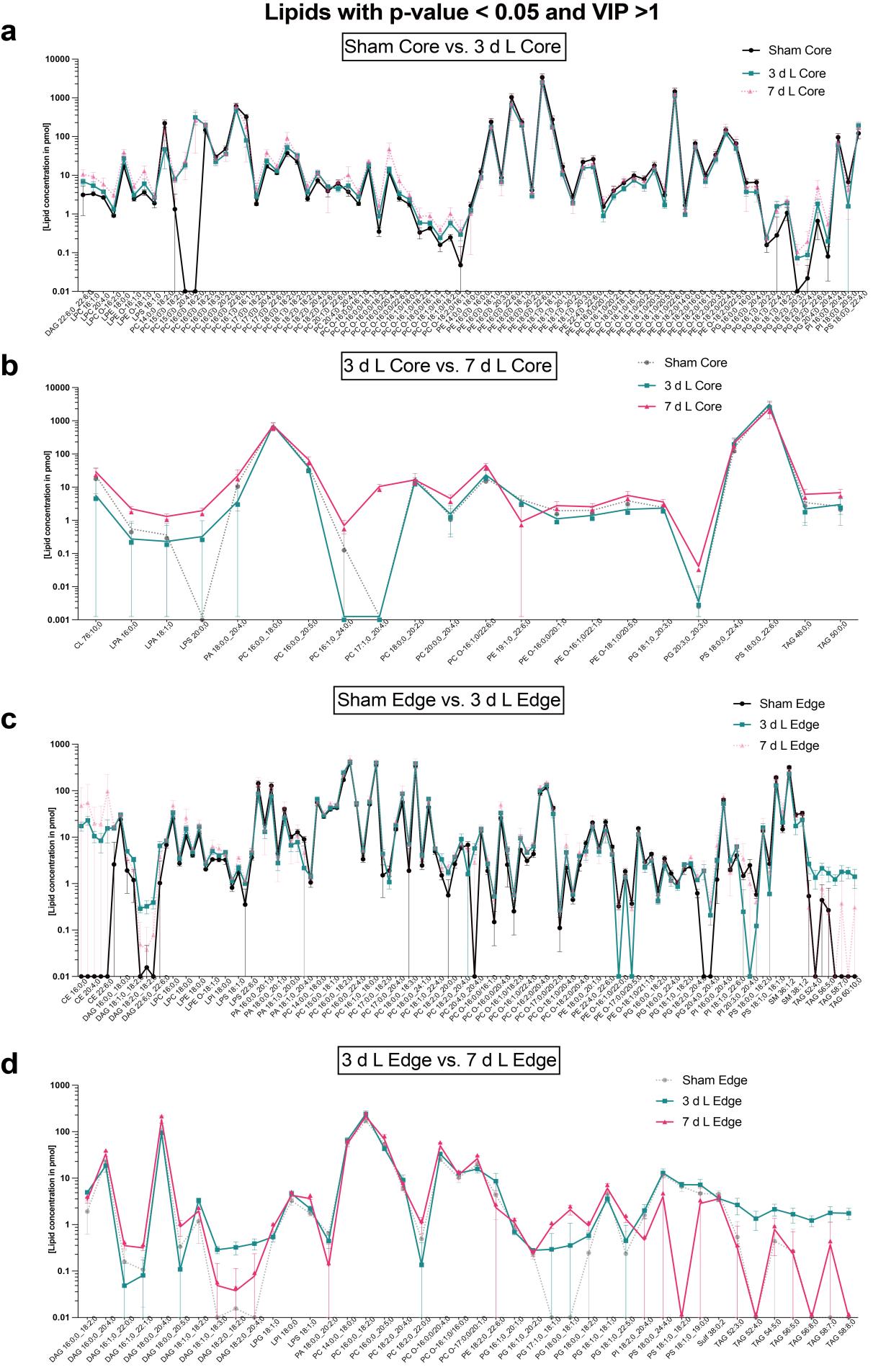
**

**S-Figure 16. Lipid profiles of edge area selected by OPLS-DA and Volcano plot.**

**a** Line plots of lipid species concentrations selected by OPLS-DA and Volcano plot analysis of Sham Core and 3 d L Core. Lipid subset features were shown. **b** Line plots of lipid species concentrations selected by OPLS-DA and Volcano plot analysis of 3 d L Core and 7 d L Core. **c** Line plots of lipid species concentrations selected by OPLS-DA and Volcano plot analysis of Sham Edge and 3 d L Edge. **d** Line plots of lipid species concentrations selected by OPLS-DA and Volcano plot analysis of 3 d L Edge and 7 d L Edge. Data are expressed as mean ± SD. In each bar plot, p-values < 0.05, VIP-score > 1. Source data are provided as a Source Data file. Abbreviation: PCA, Principal component analysis; OPLS-DA, orthogonal partial least squares discriminant analysis; L Core, lesion core of cortex; L Cortex, ipsilateral cortex out of lesion; L Edge, white matter area of lesion edge; CE, cholesteryl ester; Cer, ceramide; CL, cardiolipin; SM, sphingomyelin; DAG, diacylglycerol; TAG, triacylglycerol; LPA, lyso-phosphatidate; LPC, lyso-phosphatidyl-choline; LPC O-, ether-linked lyso-phosphatidyl-choline; LPE, lyso-phosphatidyl-ethanolamine; LPE O-, ether-linked lyso-phosphatidyl-ethanolamine; LPI, lyso-phosphatidyl-inositol; LPG, lyso-phosphatidyl-glycerol; LPS, lyso-phosphatidyl-serine; Sulf, sulfatide; HexCer, hexosylceramide; PA, phosphatidate; PC, phosphatidylcholine; PC O-, ether-linked phosphatidyl-choline; PE, phosphatidylethanolamine; PE O-, ether-linked phosphatidyl-ethanolamine; PS, phosphatidyl-serine; PI, phosphatidylinositol; PG, phosphatidylglycerol.

**Supplementary Figure S17**

**
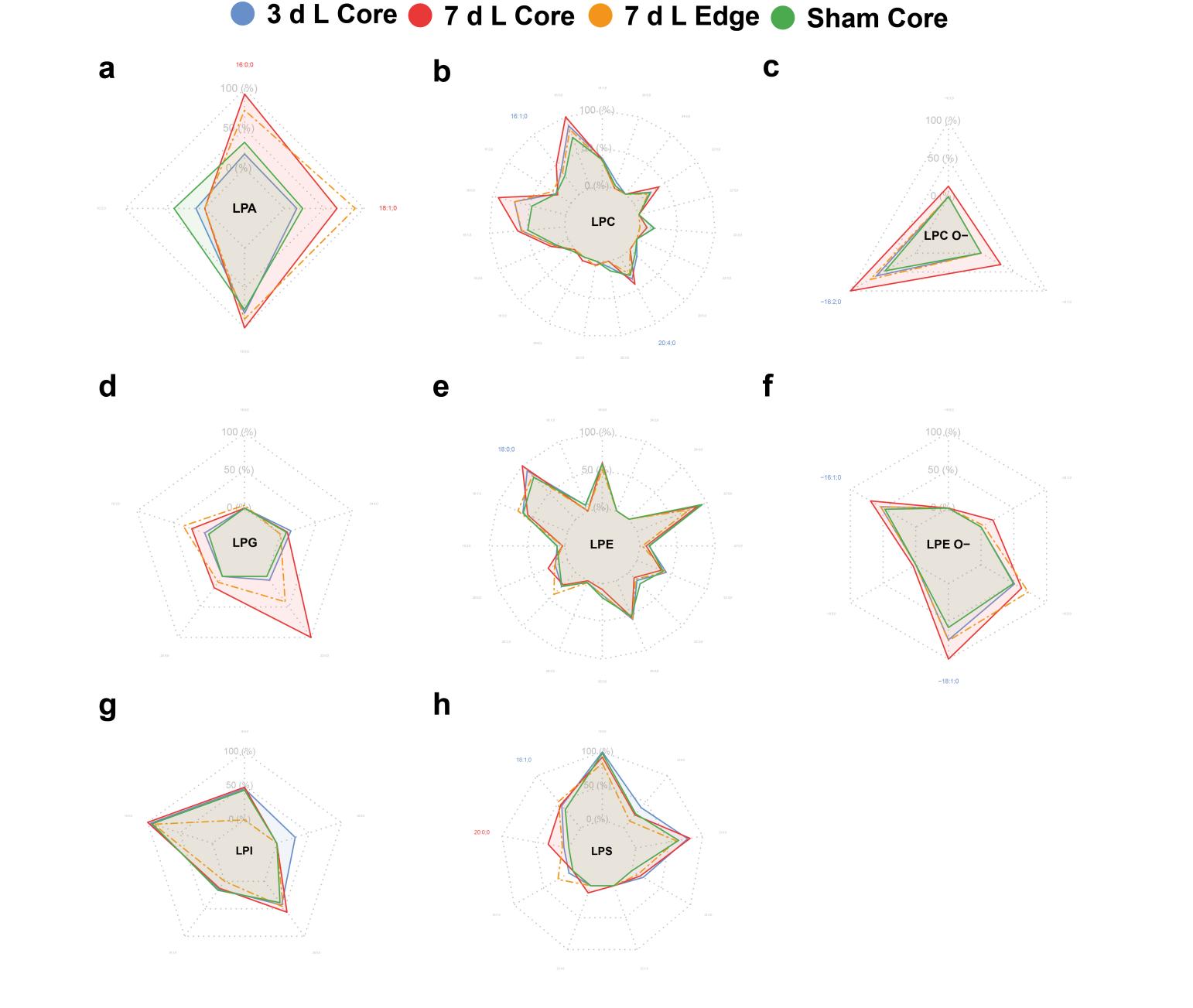
**

**S-Figure 17. Radar plots of the logarithmic mean concentrations of lyso-phospholipids sorted by classes.**

**a-h** The Radar plots display the logarithmic mean concentrations of lipids sorted by classes: LPA (**a**), LPC (**b**), LPC O- (**c**), LPG (**d**), LPE (**e**), LPE O- (**f**), LPI (**g**), and LPS (**h**). The color scheme applied to the lipid subspecies names indicates significance based on the modified t-test of the volcano plot (blue = p <0.05 for comparison of Sham core vs. 3 d L core; red = p <0.05 for comparison of 3 d L core vs. 7 d L core). Abbreviation: L Core, lesion core of cortex; L Cortex, ipsilateral cortex out of lesion; L Edge, white matter area of lesion edge; LPA, lyso-phosphatidate; LPC, lyso-phosphatidyl-choline; LPC O-, ether-linked lyso-phosphatidyl-choline; LPG, lyso-phosphatidyl-glycerol; LPE, lyso-phosphatidyl-ethanolamine; LPE O-, ether-linked lyso-phosphatidyl-ethanolamine; LPI, lyso-phosphatidyl-inositol; LPS, lyso-phosphatidyl-serine.

**Supplementary Figure S18**

**
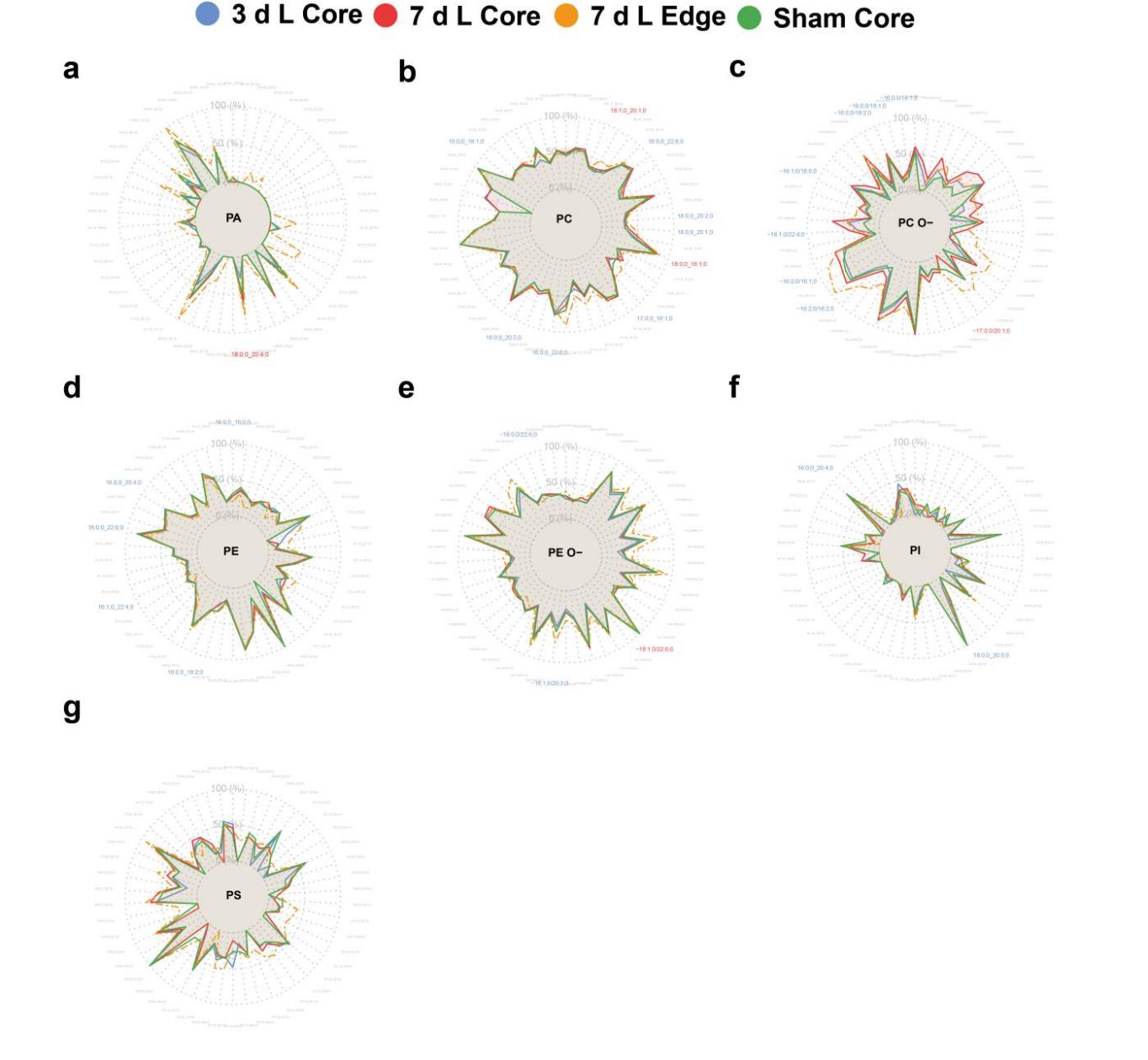
**

**S-Figure 18. Radar plots of the logarithmic mean concentrations of phospholipids sorted by classes.**

**a-g** The Radar plots display the logarithmic mean concentrations of lipids sorted by classes: PA (**a**), PC (**b**), PC O- (**c**), PE (**d**), PE O- (**e**), PI (**f**), and PS (**g**). The color scheme applied to the lipid subspecies names indicates significance based on the modified t-test of the volcano plot (blue = p <0.05 for comparison of Sham core vs. 3 d L core; red = p <0.05 for comparison of 3 d L core vs. 7 d L core). Abbreviation: L Core, lesion core of cortex; L Cortex, ipsilateral cortex out of lesion; L Edge, white matter area of lesion edge; PA, phosphatidate; PC, phosphatidylcholine; PC O-, ether-linked phosphatidyl-choline; PE, phosphatidylethanolamine; PE O-, ether-linked phosphatidyl-ethanolamine; PS, phosphatidyl-serine; PI, phosphatidylinositol.

**Supplementary Figure S19**

**
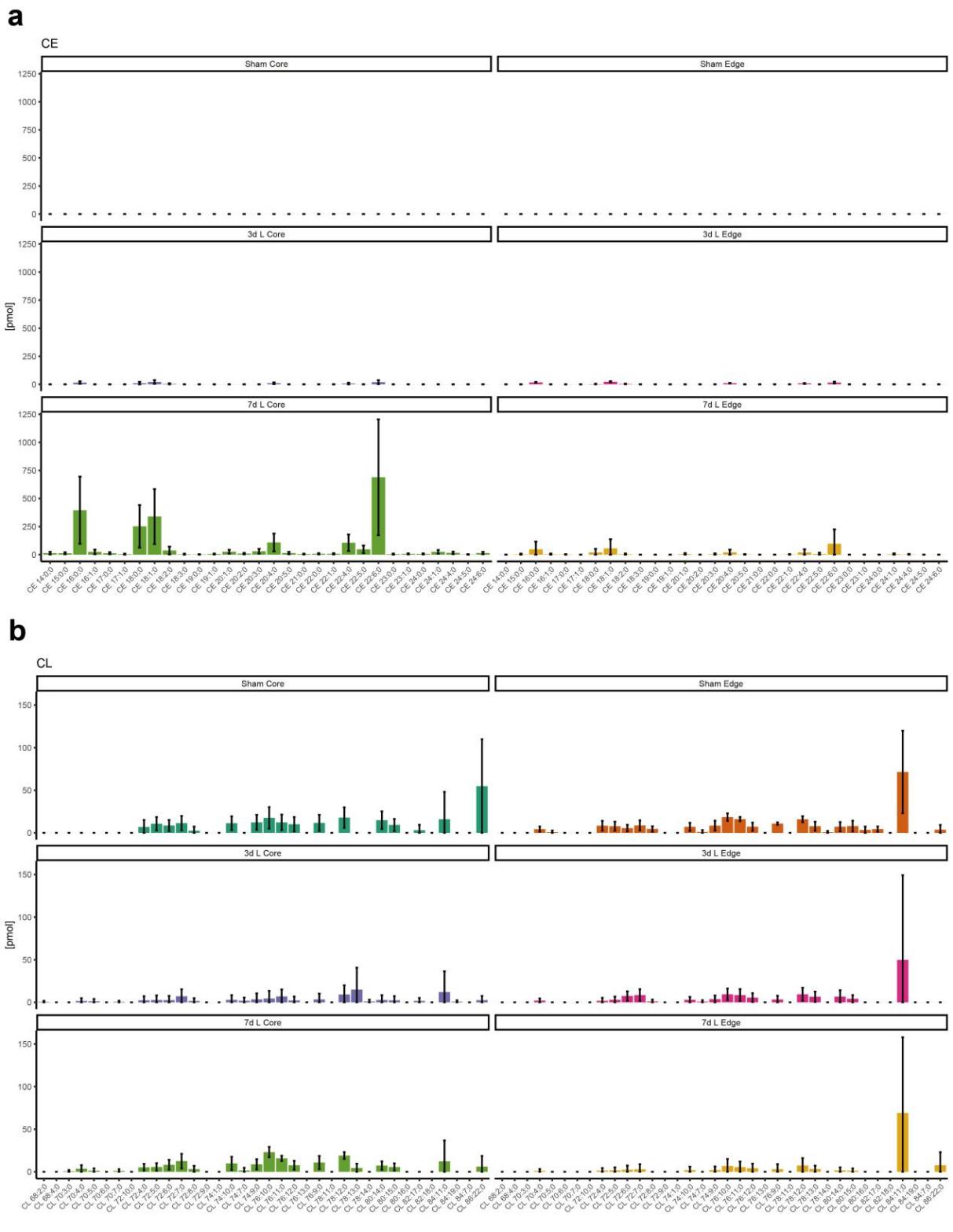
**

**S-Figure 19. Bar charts of unfiltered lipid species concentrations in pmol grouped by lipid class (Cholesteryl ester, CE; Cardiolipin, CL).**

**a** Bar plots of all lipid subsets concentrations selected from CE original data in pmol. **b** Bar plots of all lipid subsets concentrations selected from CL original data in pmol. Data are expressed as mean ± SD. Abbreviation: L Core, lesion core of cortex; L Cortex, ipsilateral cortex out of lesion; L Edge, white matter area of lesion edge; CE, cholesteryl ester; CL, cardiolipin.

**Supplementary Figure S20**

**
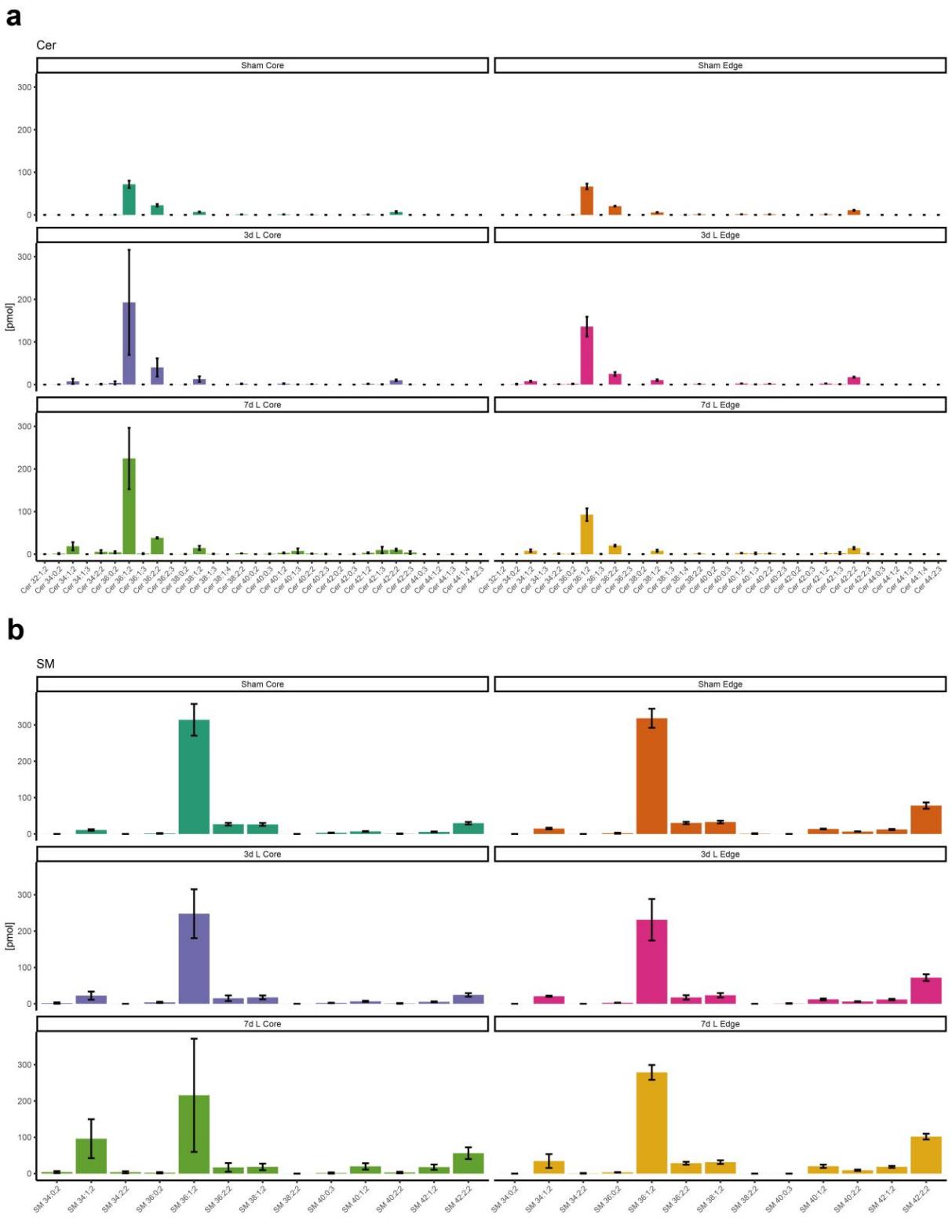
**

**S-Figure 20. Bar charts of unfiltered lipid species concentrations in pmol grouped by lipid class (Ceramide, Cer; Sphingomyelin, SM).**

**a** Bar plots of all lipid subsets concentrations selected from Cer original data in pmol. **b** Bar plots of all lipid subsets concentrations selected from SM original data in pmol. Data are expressed as mean ± SD. Abbreviation: L Core, lesion core of cortex; L Cortex, ipsilateral cortex out of lesion; L Edge, white matter area of lesion edge; Cer, ceramide; SM, sphingomyelin.

**Supplementary Figure S21**

**
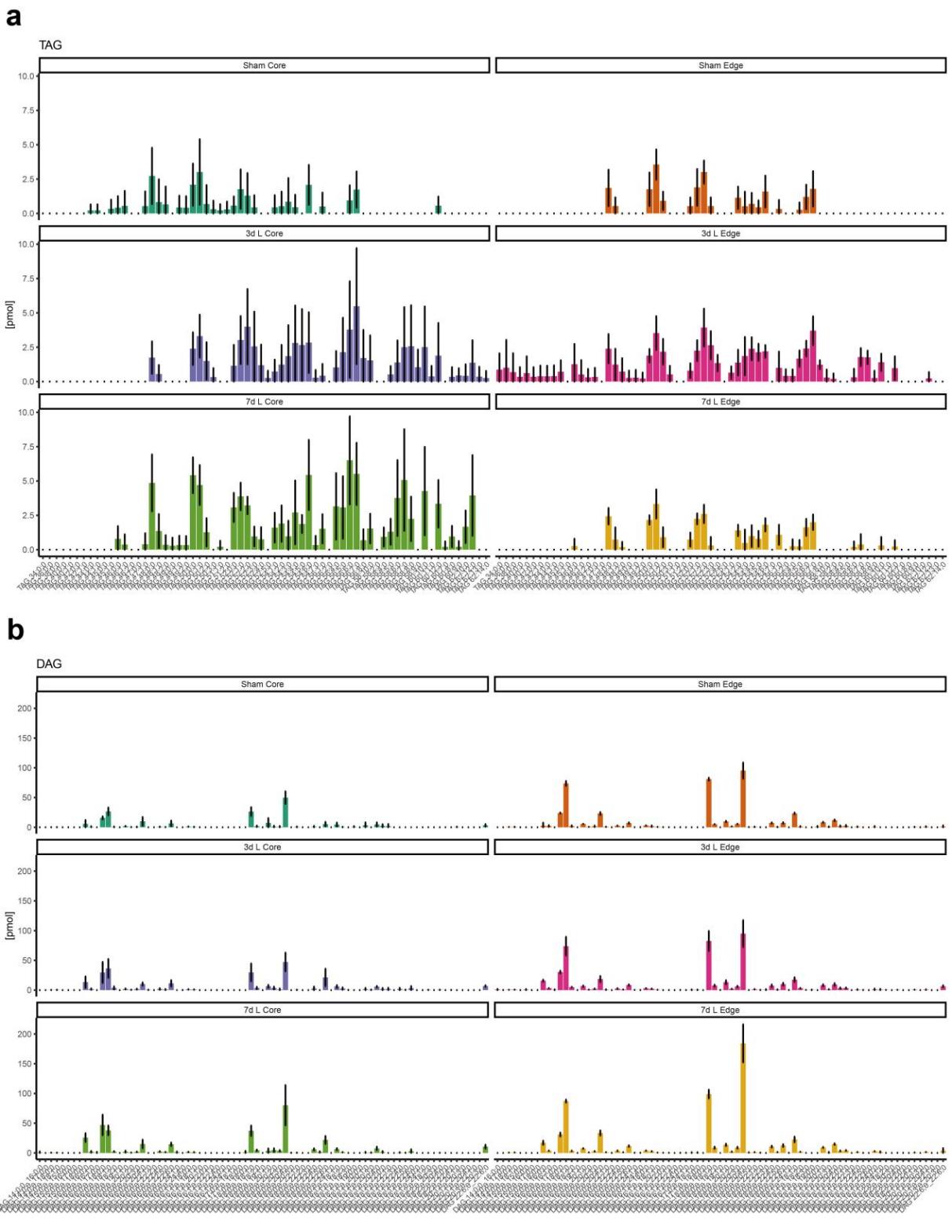
**

**S-Figure 21. Bar charts of unfiltered lipid species concentrations in pmol grouped by lipid class (Triacylglycerol, TAG; Diacylglycerol, DAG).**

**a** Bar plots of all lipid subsets concentrations selected from TAG original data in pmol. **b** Bar plots of all lipid subsets concentrations selected from DAG original data in pmol. Data are expressed as mean ± SD. Abbreviation: L Core, lesion core of cortex; L Cortex, ipsilateral cortex out of lesion; L Edge, white matter area of lesion edge; DAG, diacylglycerol; TAG, triacylglycerol.

**Supplementary Figure S22**

**
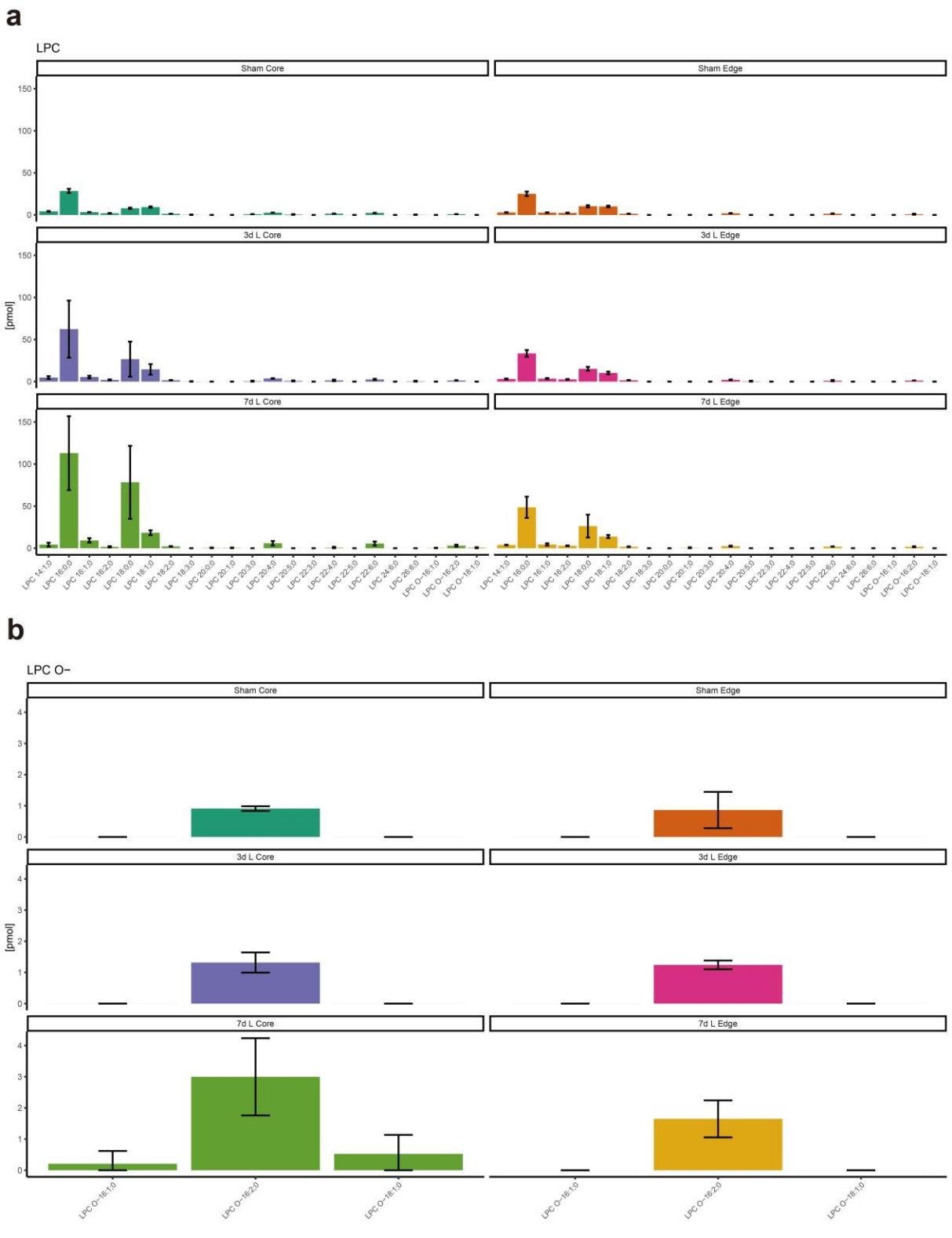
**

**S-Figure 22. Bar charts of unfiltered lipid species concentrations in pmol grouped by lipid class (Lyso-phosphatidyl-choline, LPC; Ether-linked lyso-phosphatidyl-choline, LPC O-).**

**a** Bar plots of all lipid subsets concentrations selected from LPC original data in pmol. **b** Bar plots of all lipid subsets concentrations selected from LPC O- original data in pmol. Data are expressed as mean ± SD. Abbreviation: L Core, lesion core of cortex; L Cortex, ipsilateral cortex out of lesion; L Edge, white matter area of lesion edge; LPC, lyso-phosphatidyl-choline; LPC O-, ether-linked lyso-phosphatidyl-choline.

**Supplementary Figure S23**

**
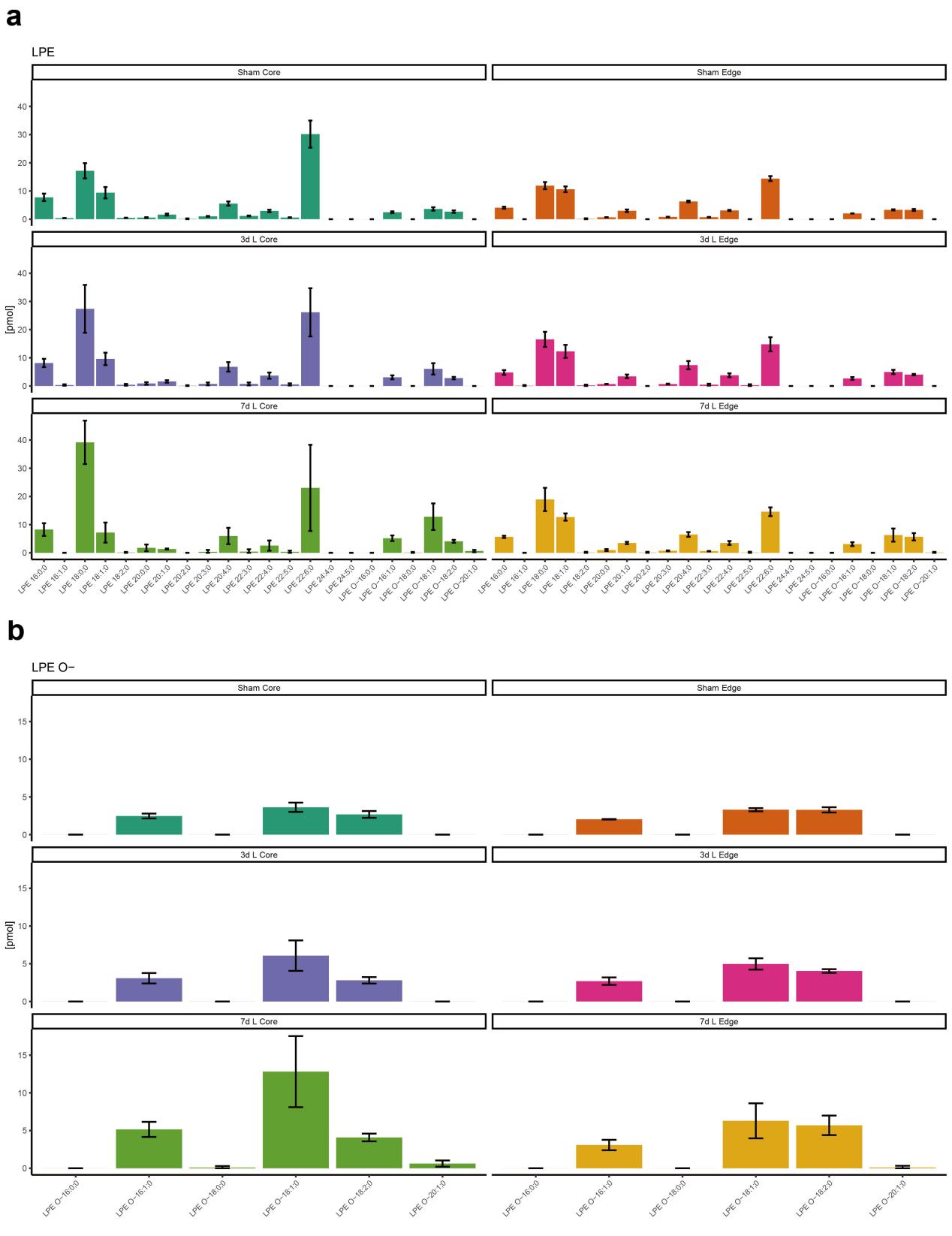
**

**S-Figure 23. Bar charts of unfiltered lipid species concentrations in pmol grouped by lipid class (Lyso-phosphatidyl-ethanolamine, LPE; Ether-linked lyso-phosphatidyl-ethanolamine, LPE O-).**

**a** Bar plots of all lipid subsets concentrations selected from LPE original data in pmol. **b** Bar plots of all lipid subsets concentrations selected from LPE O- original data in pmol. Data are expressed as mean ± SD. Abbreviation: L Core, lesion core of cortex; L Cortex, ipsilateral cortex out of lesion; L Edge, white matter area of lesion edge; LPE, lyso-phosphatidyl-ethanolamine; LPE O-, ether-linked lyso-phosphatidyl-ethanolamine.

**Supplementary Figure S24**

**
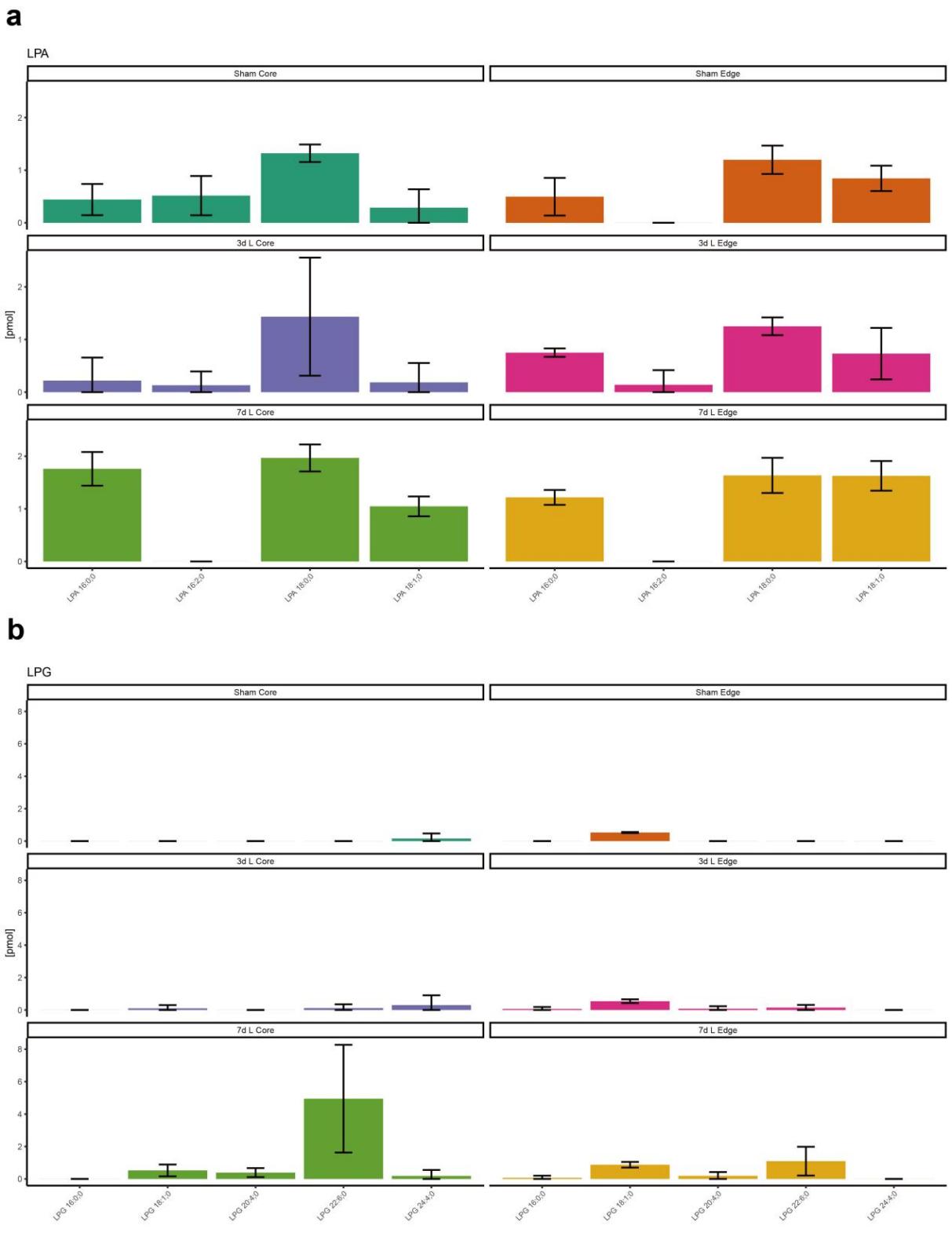
**

**S-Figure 24. Bar charts of unfiltered lipid species concentrations in pmol grouped by lipid class (Lyso-phosphatidate, LPA; Lyso-phosphatidyl-glycerol, LPG).**

**a** Bar plots of all lipid subsets concentrations selected from LPA original data in pmol. **b** Bar plots of all lipid subsets concentrations selected from LPG original data in pmol. Data are expressed as mean ± SD. Abbreviation: L Core, lesion core of cortex; L Cortex, ipsilateral cortex out of lesion; L Edge, white matter area of lesion edge; LPA, lyso-phosphatidate; LPG, lyso-phosphatidyl-glycerol.

**Supplementary Figure S25**

**
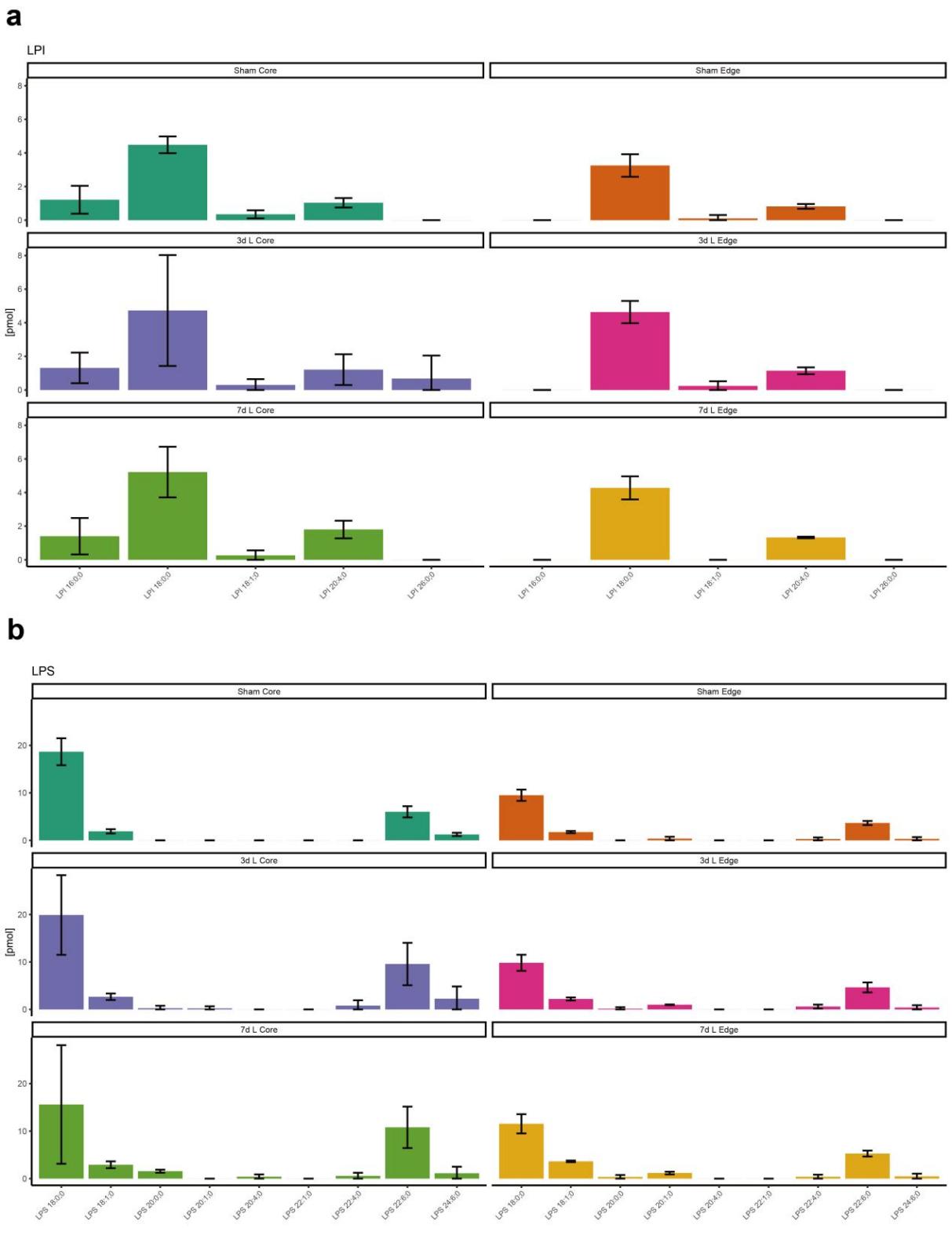
**

**S-Figure 25. Bar charts of unfiltered lipid species concentrations in pmol grouped by lipid class (Lyso-phosphatidyl-inositol, LPI; Lyso-phosphatidyl-serine, LPS).**

**a** Bar plots of all lipid subsets concentrations selected from LPI original data in pmol. **b** Bar plots of all lipid subsets concentrations selected from LPS original data in pmol. Data are expressed as mean ± SD. Abbreviation: L Core, lesion core of cortex; L Cortex, ipsilateral cortex out of lesion; L Edge, white matter area of lesion edge; LPI, lyso-phosphatidyl-inositol; LPS, lyso-phosphatidyl-serine.

**Supplementary Figure S26**

**
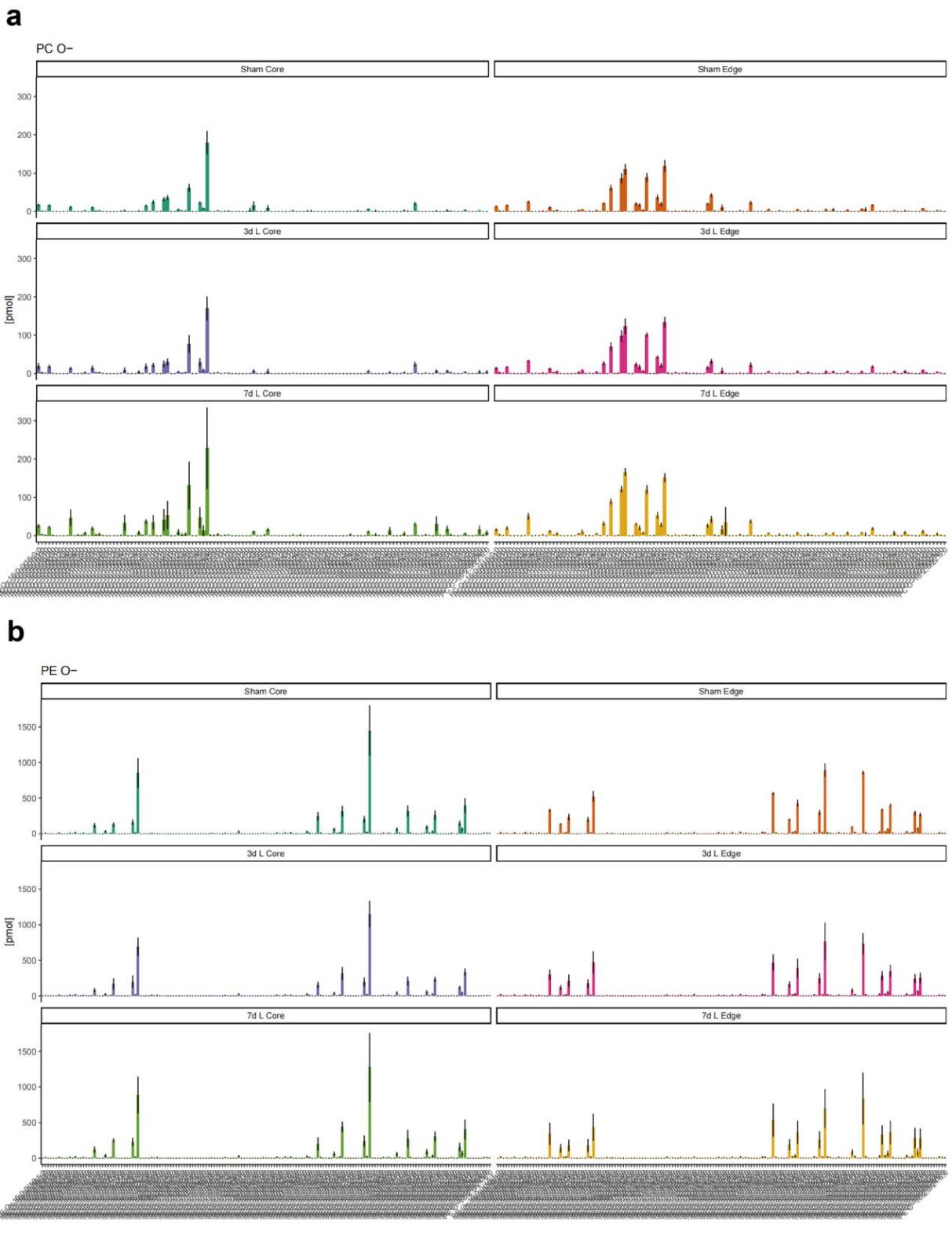
**

**S-Figure 26. Bar charts of unfiltered lipid species concentrations in pmol grouped by lipid class (Ether-linked phosphatidyl-choline, PC O-; Ether-linked phosphatidyl-ethanolamine, PE O-).**

**a** Bar plots of all lipid subsets concentrations selected from PC O- original data in pmol. **b** Bar plots of all lipid subsets concentrations selected from PE O- original data in pmol. Data are expressed as mean ± SD. Abbreviation: L Core, lesion core of cortex; L Cortex, ipsilateral cortex out of lesion; L Edge, white matter area of lesion edge; PC O-, ether-linked phosphatidyl-choline; PE O-, ether-linked phosphatidyl-ethanolamine.

**Supplementary Figure S27**

**
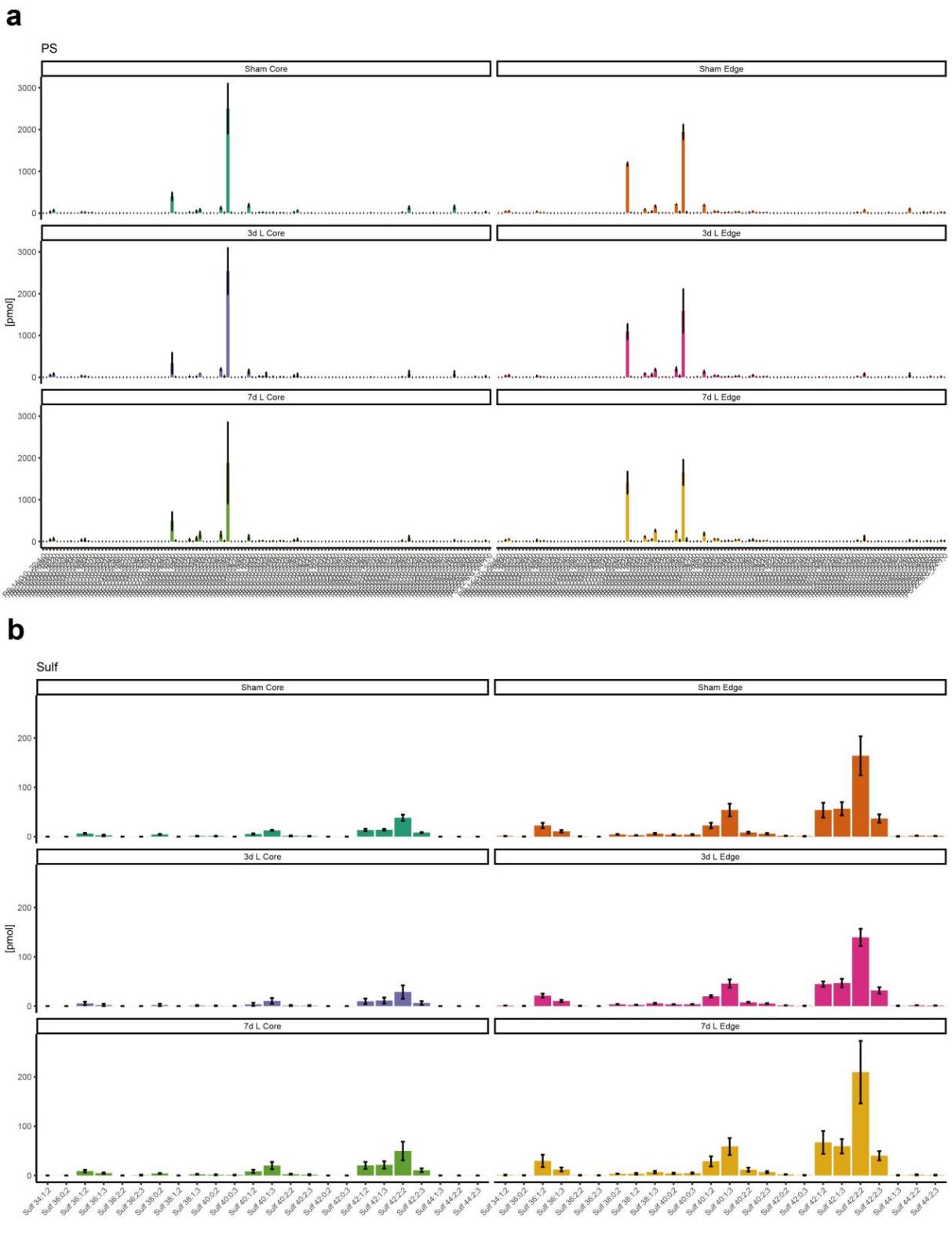
**

**S-Figure 27. Bar charts of unfiltered lipid species concentrations in pmol grouped by lipid class (Phosphatidyl-serine, PS;Sulfatide, Sulf).**

**a** Bar plots of all lipid subsets concentrations selected from PS original data in pmol. **b** Bar plots of all lipid subsets concentrations selected from Sulf original data in pmol. Data are expressed as mean ± SD. Abbreviation: L Core, lesion core of cortex; L Cortex, ipsilateral cortex out of lesion; L Edge, white matter area of lesion edge; PS, phosphatidyl-serine; Sulf, sulfatide.

**Supplementary Figure S28**

**
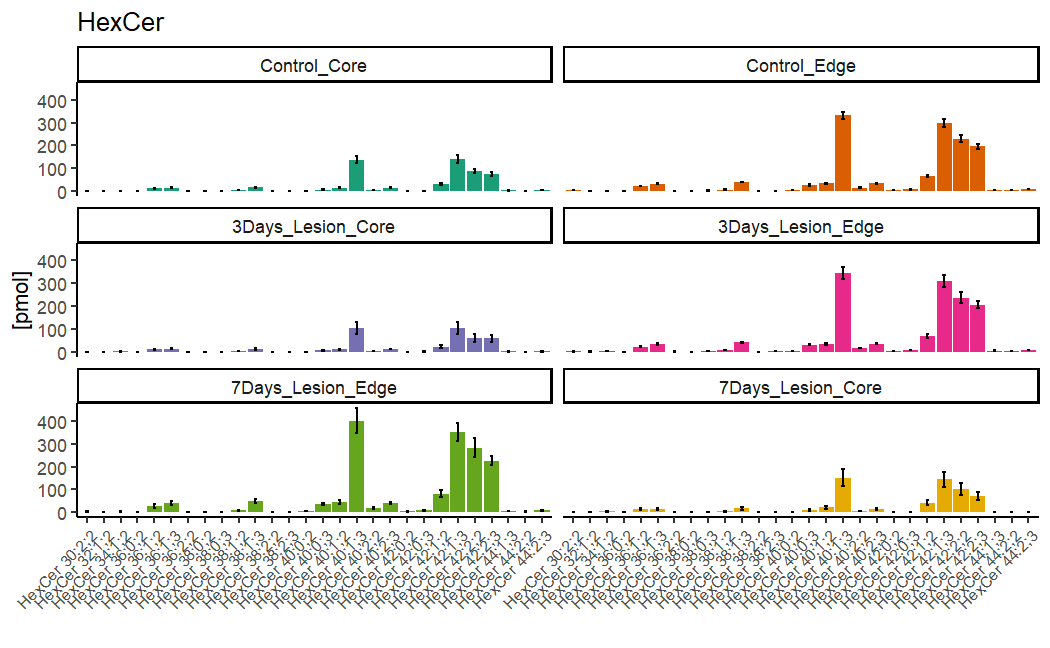
**

**S-Figure 28. Bar charts of unfiltered lipid species concentrations in pmol grouped by lipid class (hexosylceramide, HexCer).**

Bar plots of all lipid subsets concentrations selected from HexCer original data in pmol. Data are expressed as mean ± SD. Abbreviation: L Core, lesion core of cortex; L Cortex, ipsilateral cortex out of lesion; L Edge, white matter area of lesion edge; HexCer, hexosylceramide.

**Supplementary Table S1-Experimental groups and survival rates of mice.**

|  | **Lipid profile and immunofluorescence** | | | **Western blot and qRT-PCR analysis** | | | **FACS** | | |
| --- | --- | --- | --- | --- | --- | --- | --- | --- | --- |
| **Groups** | Number | Dead | Survival | Number | Dead | Survival | Number | Dead | Survival |
| **Sham** | 10 | 0 | 100.0 % | 7 | 0 | 100 % | 7 | 0 | 100 % |
| **MCAO** | 25(1) | 6 | 80.6 % | 16 (1) | 4 | 80 % | 8 | 1 | 83.3 % |

A total of 86 mice was used in the *in vivo* experiments including lipid profile analysis, immunofluorescence staining, Western blot analysis, qRT-PCR, and FACS analysis. Five mice in the MCAO group and two mice in Trc group died because of severe strokes. Three mice in the MCAO group were sacrificed because of severe pain on the second day after surgery. Two mouse in the MCAO group was excluded because of unsuccessful surgery. There was no animal dead in the remaining groups before reaching the end time points. Abbreviations: FACS, fluorescence-activated cell sorting; MCAO, middle cerebral artery occlusion.

**Supplementary Table S2-Major Resources**

| **REAGENT/RESOURCE** | **SOURCE** | **IDENTIFIER** | **APPLICATION** |
| --- | --- | --- | --- |
| **Ⅰ. Antibodies** | | | |
| **Primary antibodies** | | | |
| **Iba1 anti-goat** | WAKO | 011-27911 | 2 µg/mL (for IF) |
| **CD11b** | Abcam | ab75476 | 2 µg/mL (for IF) |
| **CX3CR1** | Thermo Fisher Scientific | PA5-19910 | 2 µg/mL (for IF) |
| **CD68** | BioRad | MCA341F | 2 µg/mL (for IF) |
| **GFAP** | Invitrogen | 13-0300 | 2 µg/mL (for IF) |
| **iNOS** | Abcam | ab15323 | 2 µg/mL (for IF) |
| **Iba1 anti-rabbit** | WAKO | 019-19741 | 2 µg/mL (for IF) |
| **P2Y12** | [Alomone Labs](https://www.alomone.com/p/anti-p2y12-receptor-atto-594/APR-012-AR) | APR-012 | 2 µg/mL (for IF) |
| **TMEM119** | Abcam | ab209064 | 2 µg/mL (for IF) |
| **PLIN3** | Progen | G37 | 2 µg/mL (for IF), 0.5 µg/mL (for WB) |
| **PLIN2** | Progen | G42 | 2 µg/mL (for IF), 0.5 µg/mL (for WB) |
| **CD206 (Mannose Receptor)** | Abcam | ab64693 | 2 µg/mL (for IF) |
| **NeuN** | Millipore | MAB377 | 4 µg/mL (for IF) |
| **Arginase I** | Santa Cruz | sc-20150 | 4 µg/mL (for IF) |
| **BODIPY 493/503** | Thermo Fisher | D3922 | 1 µg/mL (for IF), 0.5 µg/mL (for FACS) |
| **TGF-β1** | Abcam | ab92486 | 0.5 µg/mL (for WB) |
| **GAPDH** | GeneTex | GTX627408 | 0.1 µg/mL (for WB) |
| **NF-κB p65** | Abcam | ab16502 | 0.5 µg/mL (for WB) |
| **IL-1ß** | Abcam | ab9722 | 0.5 µg/mL (for WB) |
| **TGF-ß1** | Abcam | ab92486 | 0.5 µg/mL (for WB) |
| **IκBα** | Cell Signaling | #9242 | 0.5 µg/mL (for WB) |
| **SREBP2** | Abcam | PA5-88943 | 0.5 µg/mL (for WB) |
| **β-actin** | Abcam | ab6276 | 0.2 µg/mL (for WB) |
| **α-tubulin** | GeneTex | GTX628802 | 0.1 µg/mL (for WB) |
| **CD45** | BD Biosciences | 563891 | 20 µg/mL (for FACS) |
| **CD11b** | BD Biosciences | 552850 | 20 µg/mL (for FACS) |
| **Secondary antibodies** | | | |
| **Alexa Fluor 488 donkey anti-mouse** | Jackson Immuno | 715-547-003 | 0.5 µg/mL (for IF) |
| **Alexa Fluor 488 donkey anti-guinea pig** | Jackson Immuno | 706-545-148 | 0.5 µg/mL (for IF) |
| **Alexa Fluor 488 donkey anti-rat** | Jackson Immuno | 712-547-003 | 0.5 µg/mL (for IF) |
| **Alexa Fluor 488 donkey anti-rabbit** | Jackson Immuno | 711-547-003 | 0.5 µg/mL (for IF) |
| **Cy 3 donkey anti-rabbit** | Jackson Immuno | 711-165-152 | 0.5 µg/mL (for IF) |
| **Cy 3 donkey anti-rat** | Jackson Immuno | 712-165-153 | 0.5 µg/mL (for IF) |
| **Cy 3 donkey anti-goat** | Jackson Immuno | 705-165-003 | 0.5 µg/mL (for IF) |
| **Goat Anti-Mouse IgG H&L** | Abcam | ab97023 | 0.1 µg/mL (for WB) |
| **Goat Anti-Rabbit IgG H&L** | Abcam | ab97051 | 0.1 µg/mL (for WB) |
| **Goat Anti-Guinea pig IgG H&L** | Abcam | ab6908 | 0.1 µg/mL (for WB) |
| **Ⅱ. Chemicals and Recombinant Proteins** | | | |
| **L-glutamine** | Thermo Fisher Scientific | 25030024 | 100 μL L-glutamine in 10 mL medium |
| **Triacsin C** | Cayman Chemical | 10007448 | 2 µM in PBS |
| **Interleukin 4** | Sigma-Aldrich | SRP3211 | 10 ng/mL in 50 mL PBS with 0.1% BSA |
| **Antibiotic/antimycotic (100x)** | Thermo Fisher Scientific | 15240062 | 100 μL in 10 mL medium |
| **Thiazolyl Blue Tetrazolium Bromide** | Sigma-Aldrich | M5655 | 5 mg/mL in PBS |
| **Trypan Blue Solution 0.4%** | Thermo Fisher Scientific | 15250-061 | undiluted |
| **Trypsin-EDTA 0.25%** | Thermo Fisher Scientific | 25200-056 | undiluted |
| **Poly-D-lysine** | Sigma-Aldrich | P6407 | 5 mg PDL in 50 mL H_2_O (0.1 mg/mL) |
| **Poly-L-ornithine** | Sigma-Aldrich | P3655 | 5mg PLO in 50 mL H_2_O (0.1 mg/mL) |
| **Laminin** | Sigma-Aldrich | L2020 | 2 μg/mL in PBS |
| **Penicillin/streptomycin** | Thermo Fisher Scientific | 15140122 | 500 μL pen/strep in 50 mL medium |
| **B27** | Thermo Fisher Scientific | 17504001 | 1 mL B27 in 50 mL medium |
| **Dulbecco’s Phosphate Buffered Saline** | Sigma-Aldrich | D8537-500ML | undiluted |
| **DNase I** | Sigma-Aldrich | 11284932001 | undiluted |
| **DAPI** | AppliChem | A1001 | 1 µg/mL in PBS/TBS |
| **Dimethyl Sulfoxide** | Sigma-Aldrich | D9170 | undiluted |
| **HEPES 1M** | Sigma-Aldrich | H-4034 | 10 mM in medium |
| **HyClone Characterized Fetal Bovine Serum** | Cytiva | SH30073.02 | 10% in medium |
| **Neurobasalä Medium (1X)** | Thermo Fisher Scientific | 21103-049 | undiluted |
| **Recombinant Murine M-CSF** | Peprotech | 315-02-10UG | 5 µM in medium |
| **Lipopolysaccharides** | Sigma-Aldrich | [L3024](https://www.sigmaaldrich.com/DE/en/product/sigma/l3024) | 1 µg/mL in medium |
| **DMEM/F12 (1:1) Medium (1X)** | PAN-Biotech | P04-41150 | undiluted |
| **RIPA Lysis and Extraction Buffer** | Thermo Fisher Scientific | 89900 | undiluted |
| **Hoechst 33342** | Thermo Fisher Scientific | 62249 | 4 µM in PBS/TBS |

**WB: Western blot**

**IF: Immunofluorescence staining**

**FACS: Fluorescence-activated Cell Sorting**

**Supplementary Table S3-mRNA candidates and primer sequence**

| **mRNA** | **Sequence (5′-3′)** |
| --- | --- |
| **CD206** | Forward Sequence: CTCTGTTCAGCTATTGGACGC |
|  | Reverse Sequence: CGGAATTTCTGGGATTCAGCTTC |
| **iNOS** | Forward Sequence: AGGAACCTACCAGCTCACTCTG |
|  | Reverse Sequence: TTTCCTGTGCTGTGCTACAGTT |
| **IL-1β** | Forward Sequence: GCAACTGTTCCTGAACTCAACT |
|  | Reverse Sequence: ATCTTTTGGGGTCCGTCCAACT |
| **IL-10** | Forward Sequence: AGAAAAGAGAGCTCCATCATGC |
|  | Reverse Sequence: TTATTGTCTTCCCGGCTGTACT |
| **TNF-α** | Forward Sequence: AAGCCTGTAGCCCACGTCGTA |
|  | Reverse Sequence: GGCACCACTAGTTGGTTGTCTTTG |
| **TGF-β1** | Forward Sequence: CCTGTCCAAACTAAGGC |
|  | Reverse Sequence: GGTTTTCTCATAGATGGCG |
| **SREBP2** | Forward Sequence: AGCCAAGGAGAGCCTGTACTG |
|  | Reverse Sequence: GAGAGCGCACAGCTGCATCG |
| **PLIN2** | Forward Sequence: ACACCCTCCTGTCCAACATC |
|  | Reverse Sequence: AAGGGACCTACCAGCCAGTT |
| **ABCA1** | Forward Sequence: GCTTGTTGGCCTCAGTTAAGG  Reverse Sequence: GTAGCTCAGGCGTACAGAGAT |
| **ApoE** | Forward Sequence: CTGACAGGATGCCTAGCCG  Reverse Sequence: CGCAGGTAATCCCAGAAGC |
| **Lipa** | Forward Sequence: AGCGACGACTTGGTGTTCC  Reverse Sequence: CGCAGGTAATCCCAGAAGC |
| **Npc2** | Forward Sequence: AGGACTGCGGCTCTAAGGT  Reverse Sequence: AGGCTCAGGAATAGGGAAGGG |
| **Soat1** | Forward Sequence: GAAGGCTCACTCATTTGTCAGA  Reverse Sequence: GTCTCGGTAAATAAGTGTAGGCG |
| **Nceh1** | Forward Sequence: TTGAATACAGGCTAGTCCCACA  Reverse Sequence: CAACGTAGGTAAACTGTTGTCCC |
| **β-actin** | Forward Sequence: CGTGCGTGACATCAAAGAGA  Reverse Sequence: CCCAAGAAGGAAGGCTGGA |
| **GAPDH** | Forward Sequence: TGGATTTGGACGCATTGGTC  Reverse Sequence: TTTGCACTGGTACGTGTTGAT |
| **PPIA** | Forward Sequence: GAGCTGTTTGCAGACAAAGTTC  Reverse Sequence: CCCTGGCACATGAATCCTGG |

**Supplementary Table S4-Data of OPLS-DA analysis**

| **Groups** | **R2X(cum)** | **R2Y(cum)** | **Q2(cum)** | **RMSEE** |
| --- | --- | --- | --- | --- |
| **Sham Core vs. 3d L Core** | 0.62 | 1 | 0.667 | 0.00983 |
| **3d L Core vs. 7d L Core** | 0.632 | 0.996 | 0.57 | 0.0444 |
| **Sham Edge vs. 3d L Edge** | 0.614 | 1 | 0.762 | 0.0154 |
| **3d L Edge vs. 7d L Edge** | 0.417 | 0.99 | 0.551 | 0.0647 |

**Supplementary Table S5-Microglial markers and distribution in the post-ischemic brain**


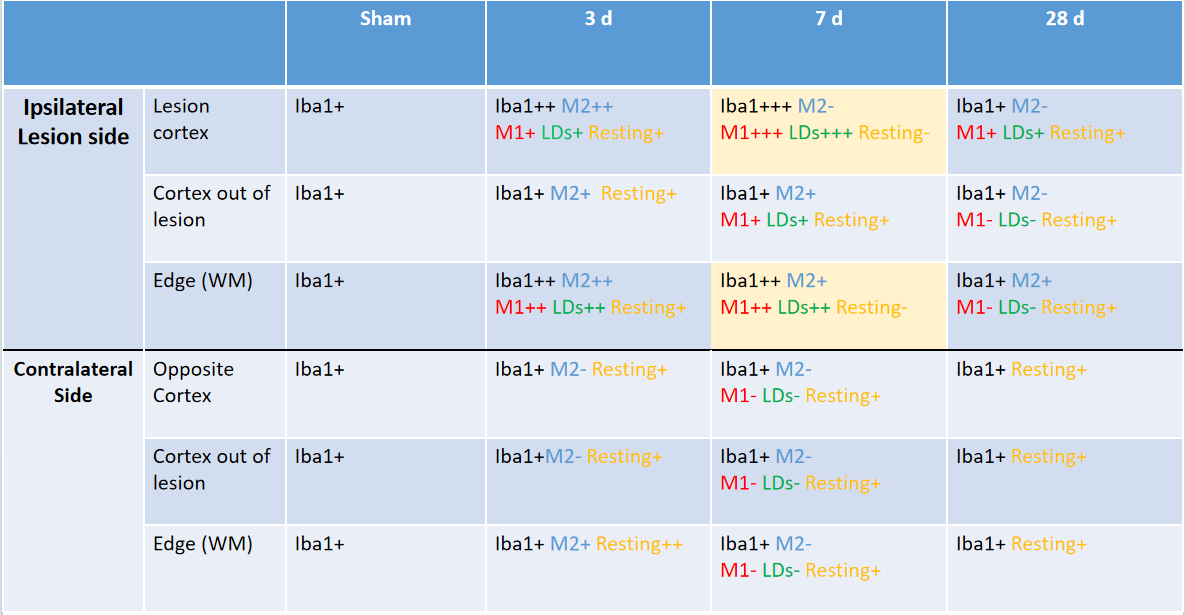


**Microglial markers:**

**Total microglia: Iba1, CD11b**

**Resting microglia: TMEM119, P2Y12**

**Activated M1-like: (LDs activated: iNOS, CD68, and IL-1β level)**

**Activated M2-like: (Arg1, CD206, and TGF-β1 level)**

**LDs: PLIN2, BODIPY**

**Supplementary full scans of Western blots**


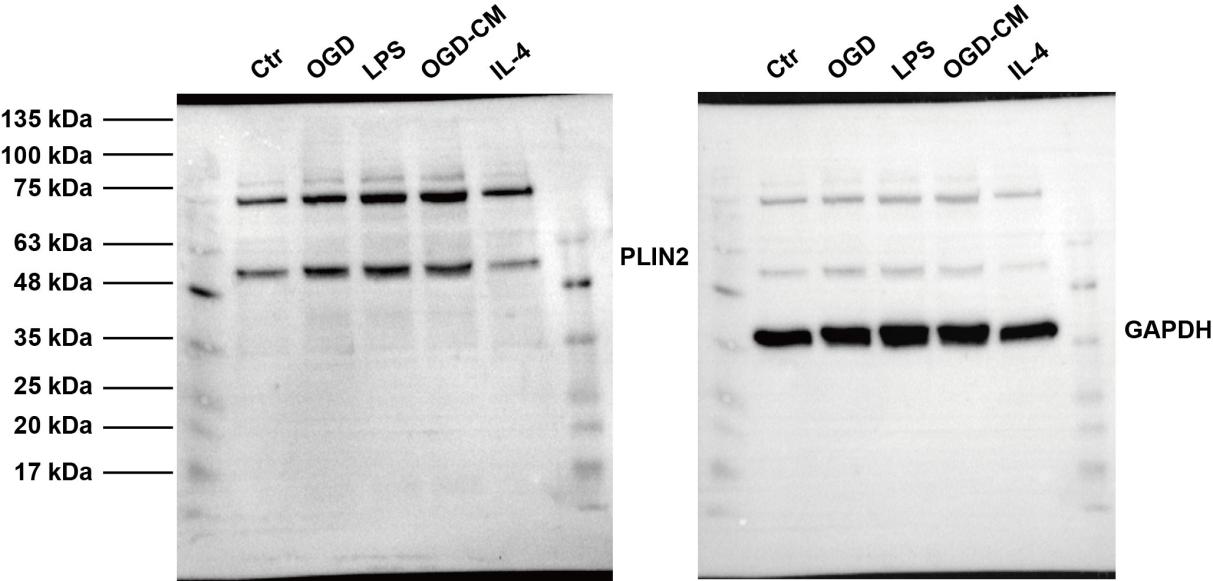


**Full scans of Western blots shown in Fig. 1j.** PLIN2 and GAPDH.


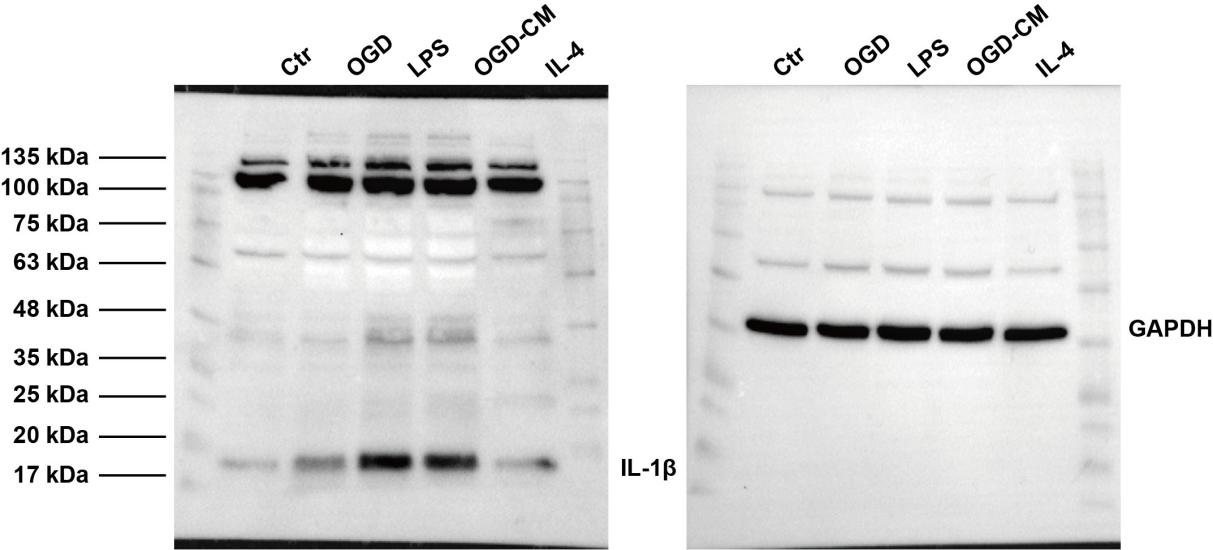


**Full scans of Western blots shown in Fig. 1k.** IL-1β and GAPDH.


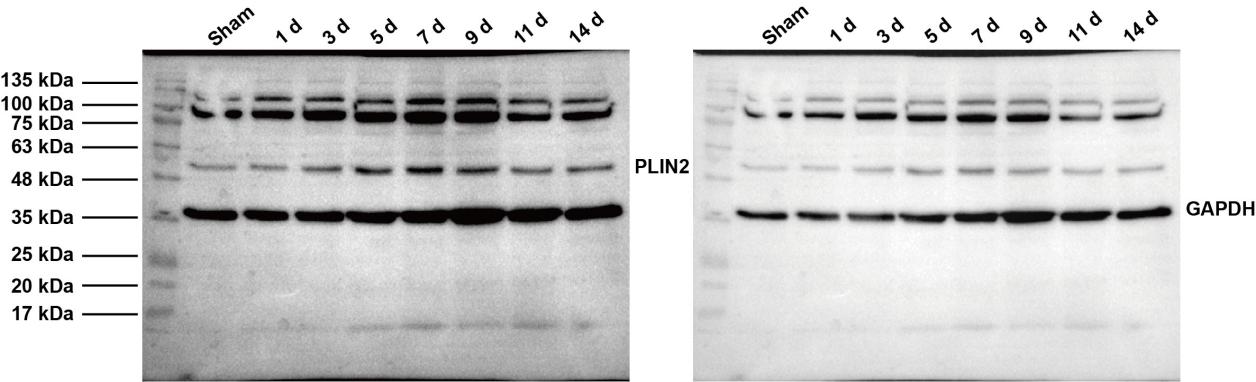


**Full scans of Western blots shown in Fig. 2j.** PLIN2 and GAPDH.


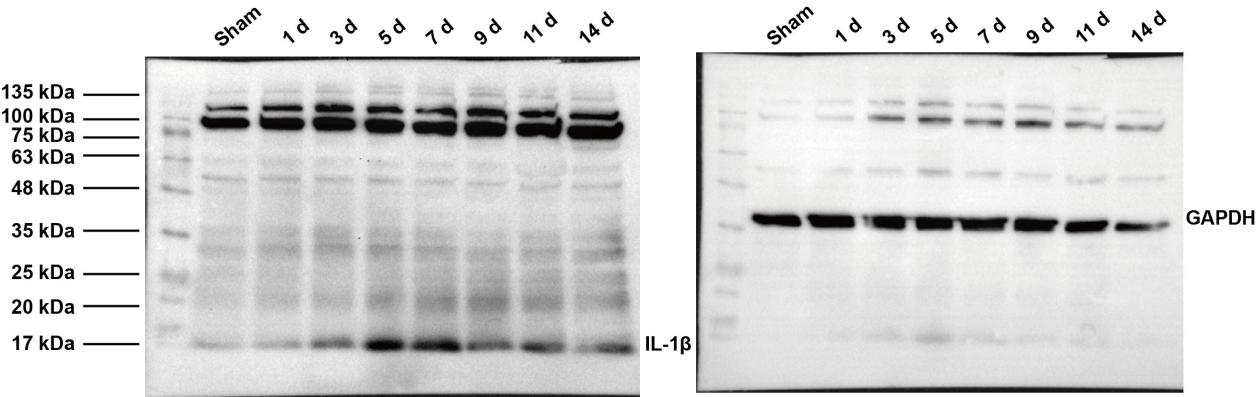


**Full scans of Western blots shown in Fig. 2k.** IL-1β and GAPDH.

**
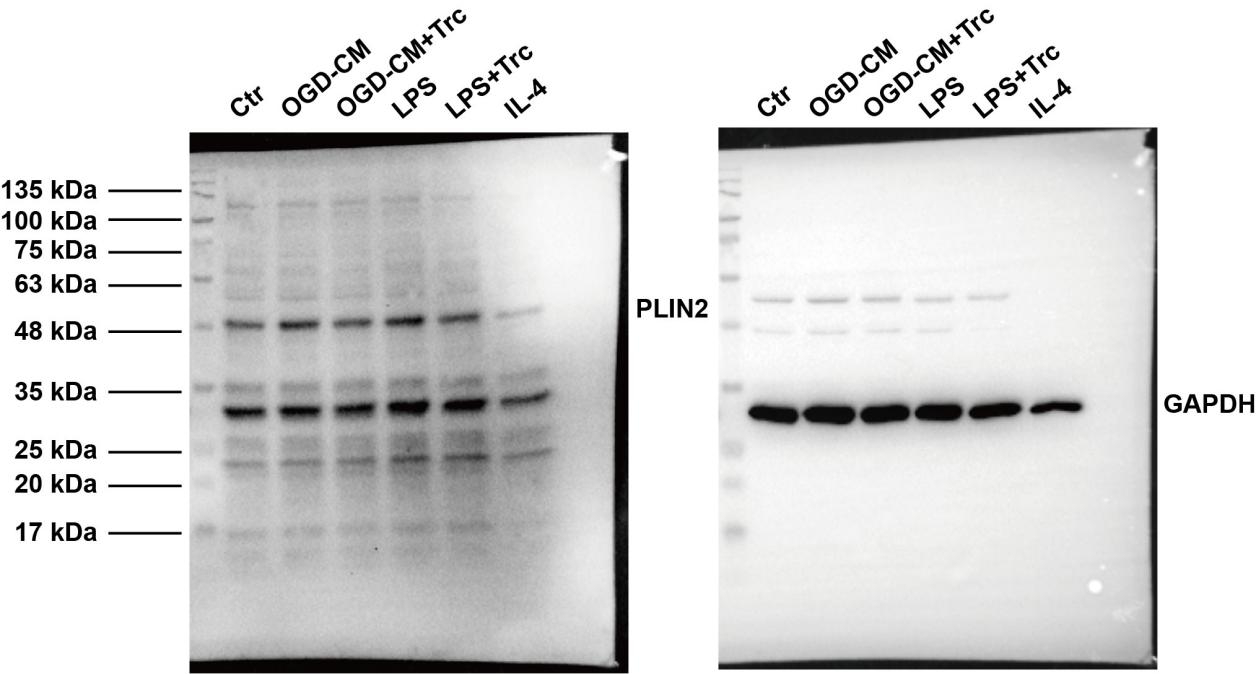
**

**Full scans of Western blots shown in Fig. 4j.** PLIN2 and GAPDH.

**
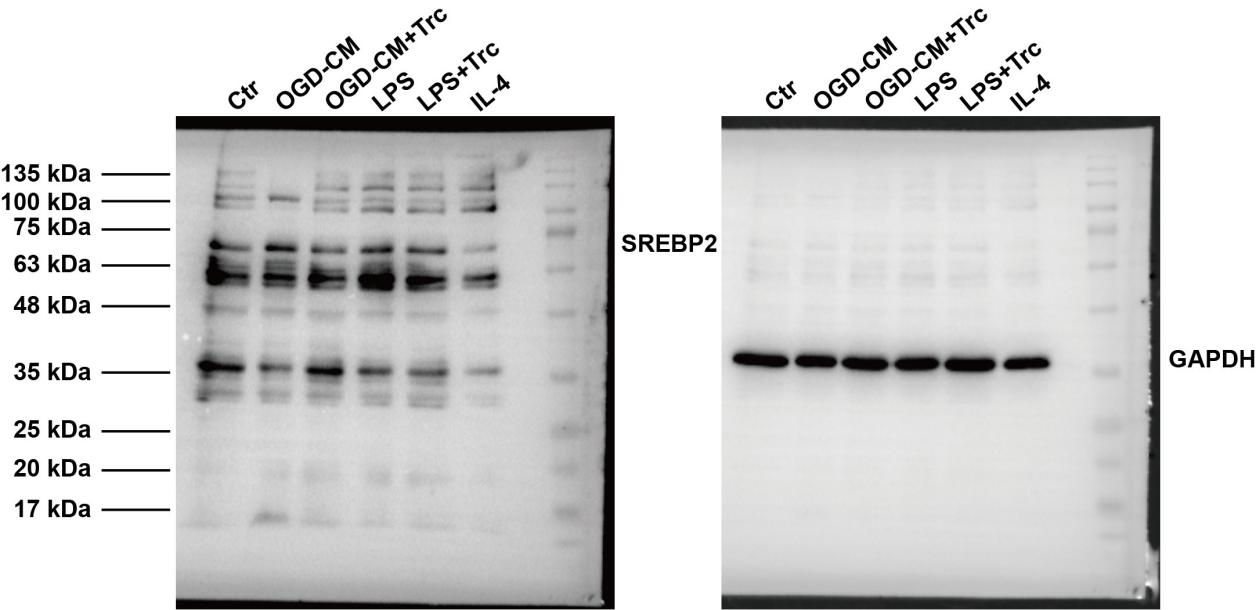
**

**Full scans of Western blots shown in Fig. 4k.** SREBP2 and GAPDH.


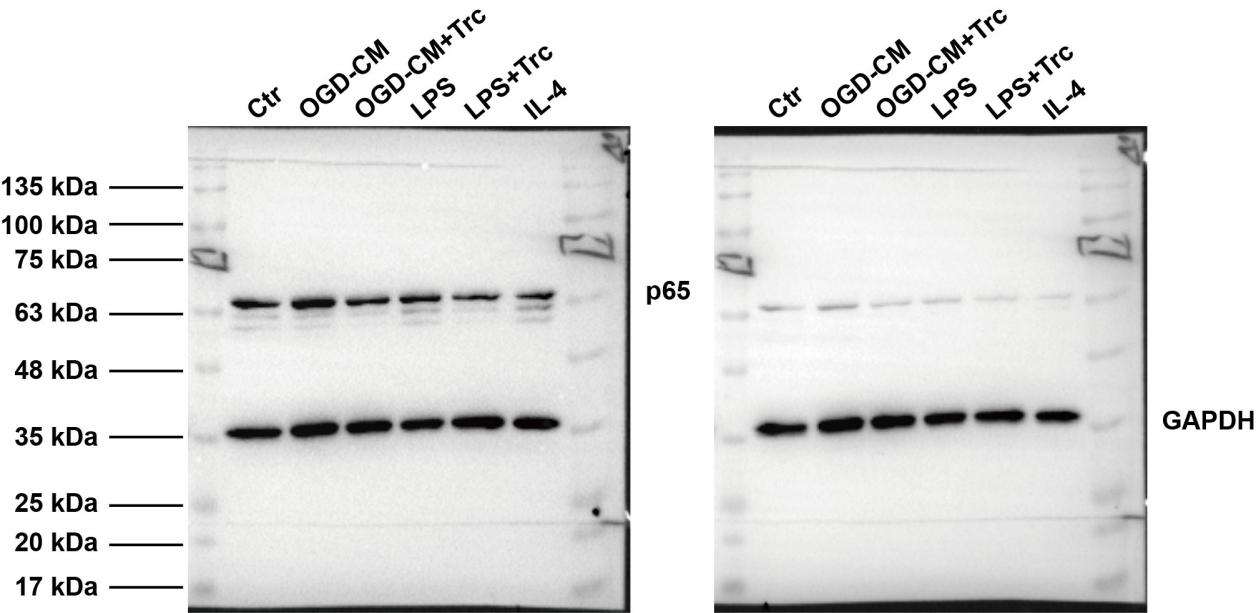


**Full scans of Western blots shown in Fig. 4l.** p65 and GAPDH.


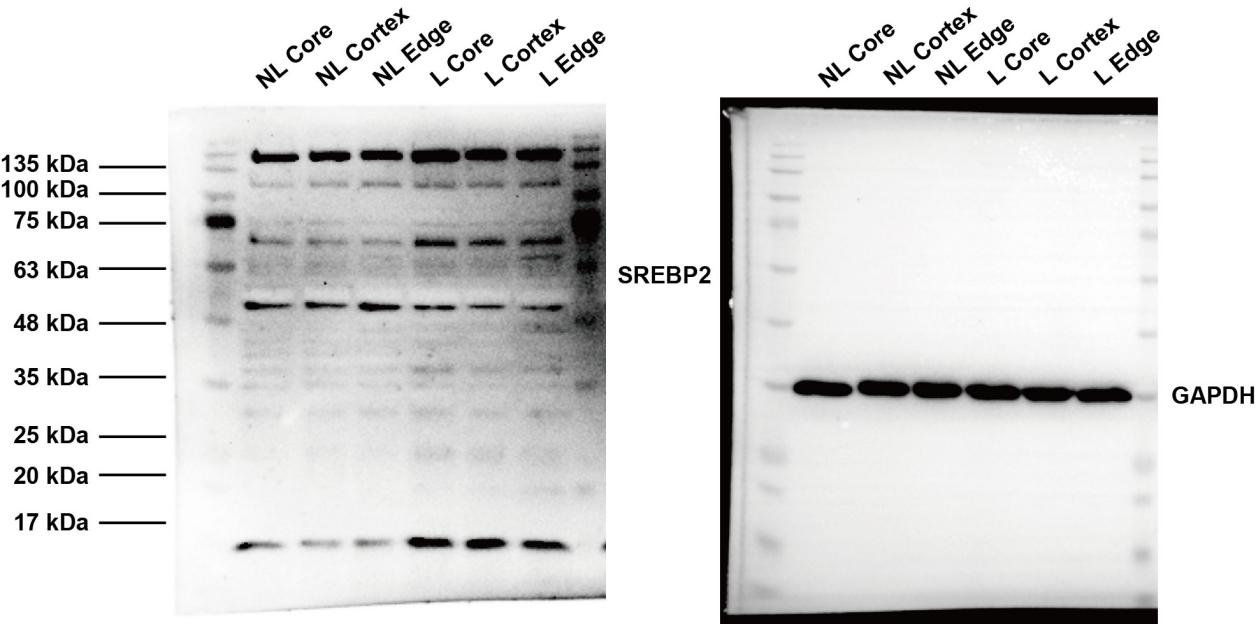


**Full scans of Western blots shown in Fig. 5e.** SREBP2 and GAPDH.


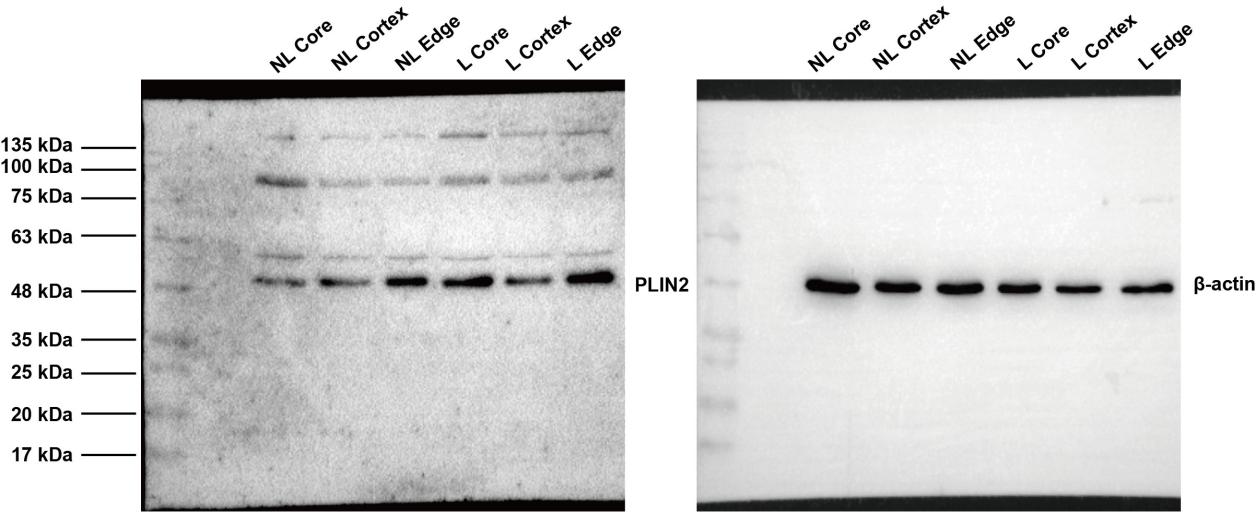


**Full scans of Western blots shown in Fig. 5e.** PLIN2 and β-actin.


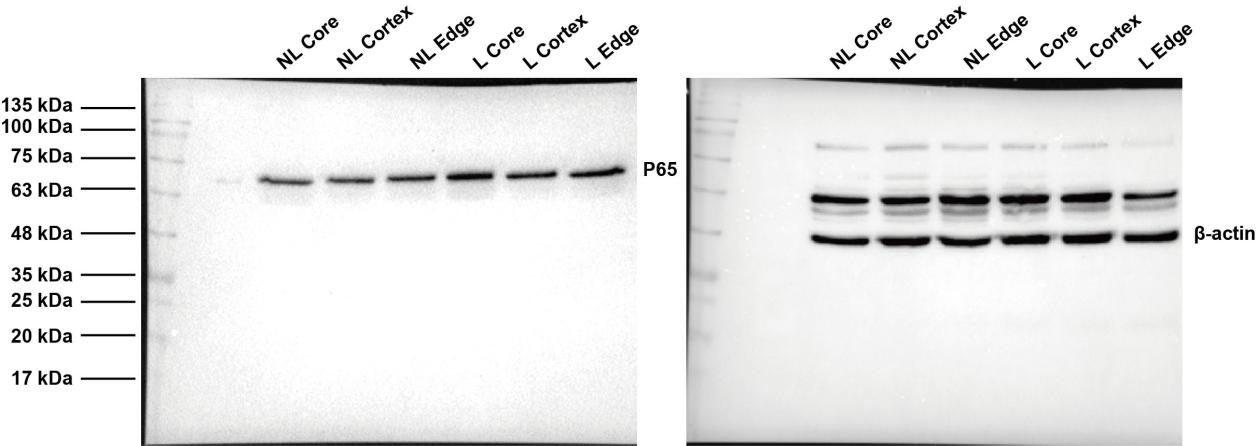


**Full scans of Western blots shown in Fig. 5e.** p65 and β-actin.

**
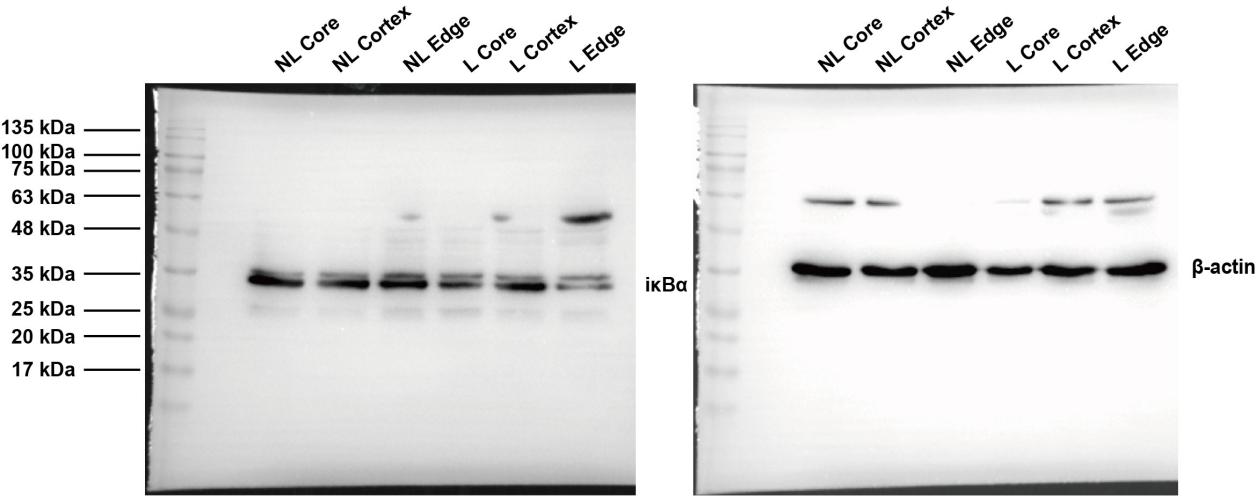
**

**Full scans of Western blots shown in Fig. 5e.** iκBα and β-actin.

**
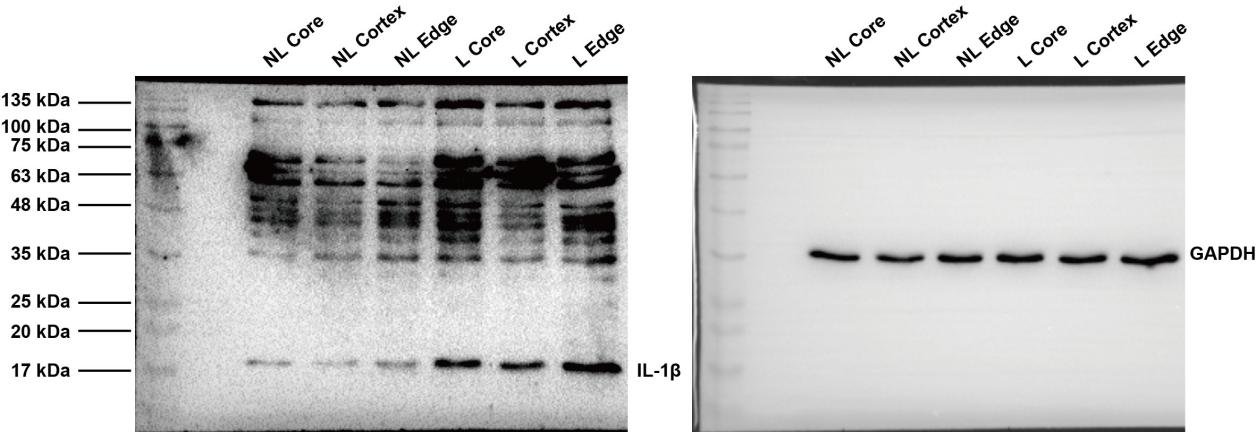
**

**Full scans of Western blots shown in Fig. 5e.** IL-1β and GAPDH.


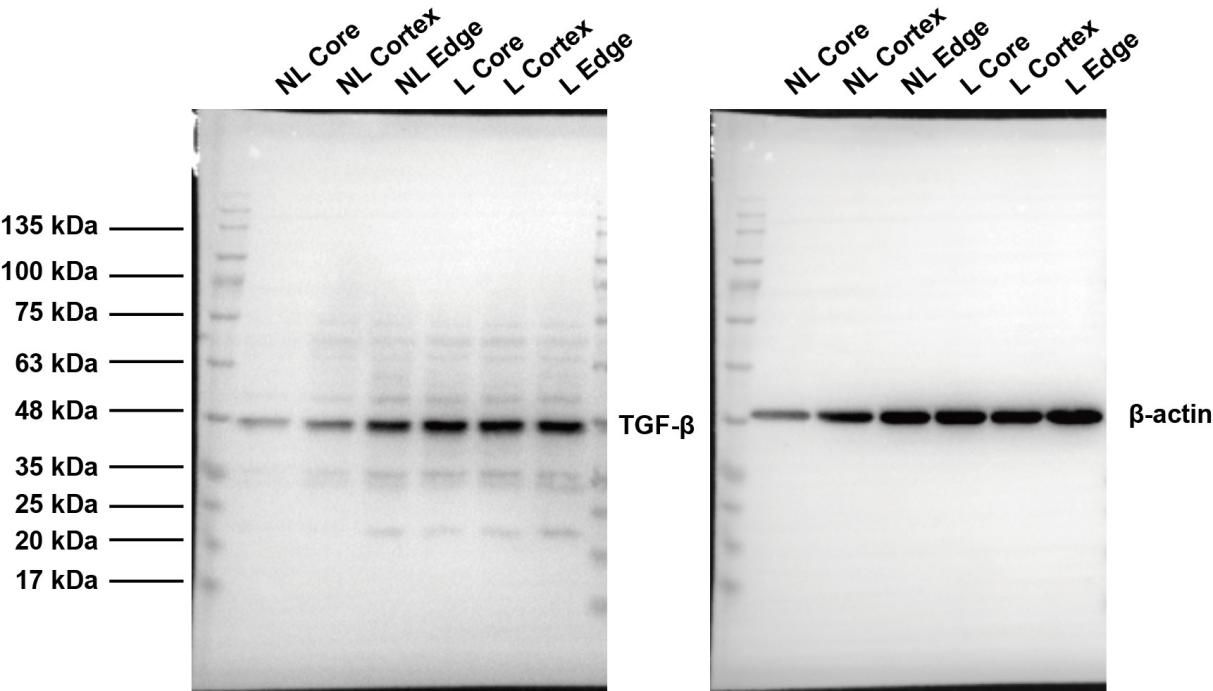


**Full scans of Western blots shown in Fig. 5e.** TGF-β1 and β-actin.


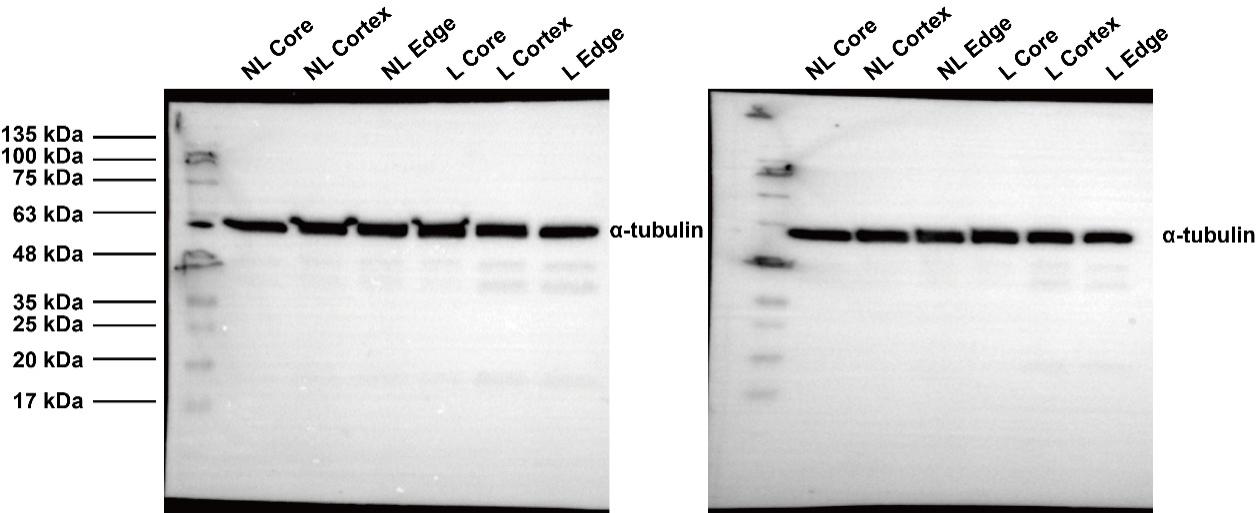


**Full scans of Western blots shown in Fig. 5e.** α-tubulin.

**
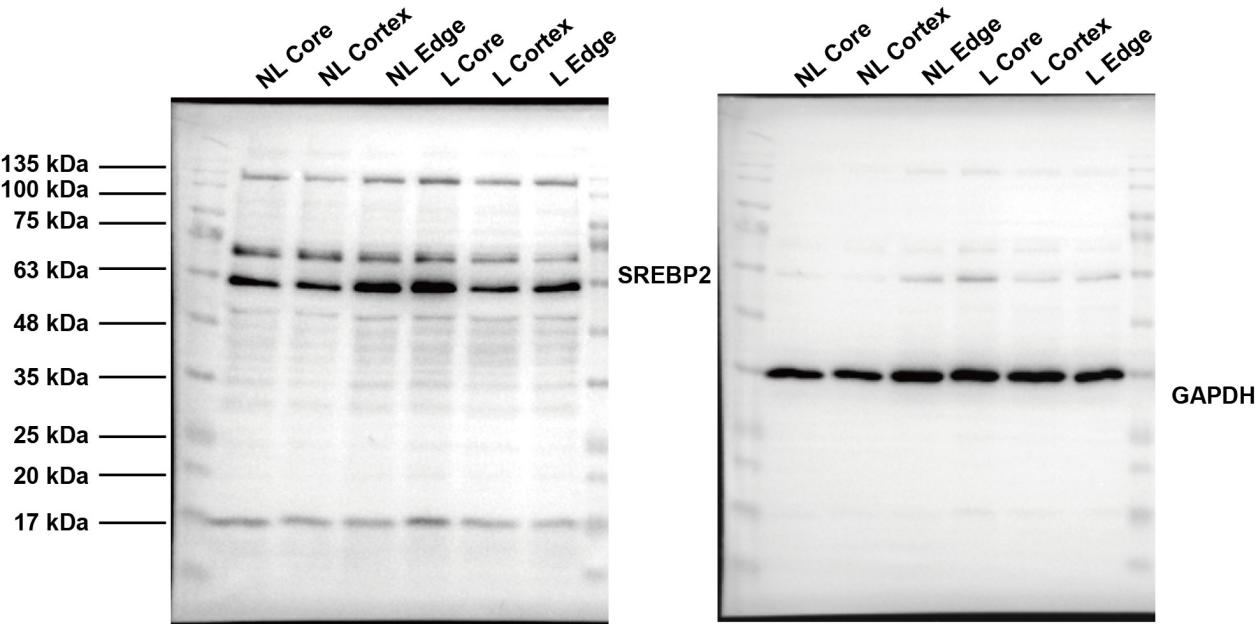
**

**Full scans of Western blots shown in Fig. 5f.** SREBP2 and GAPDH.


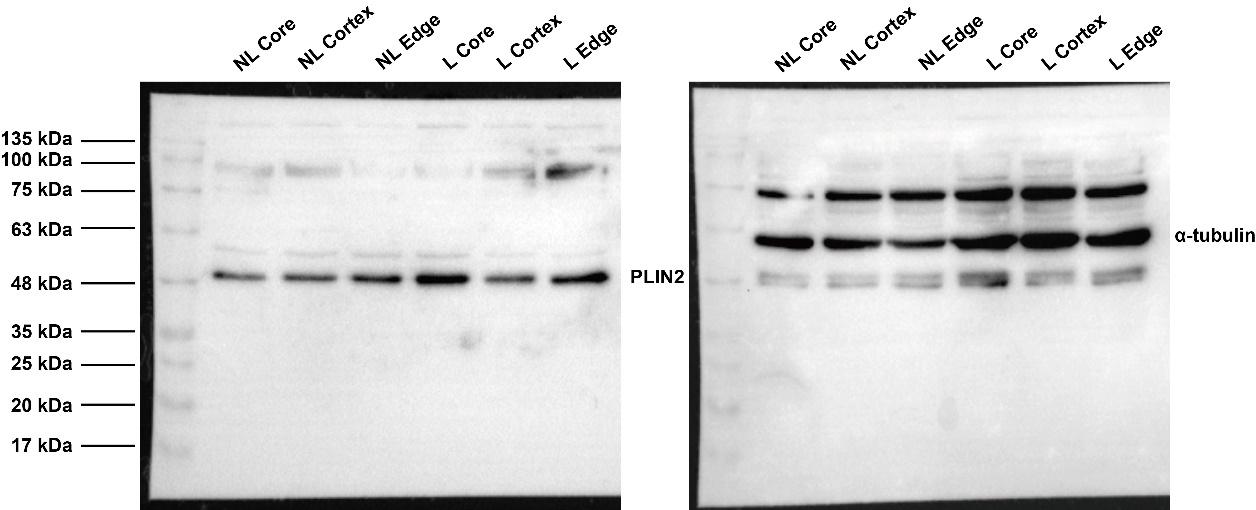


**Full scans of Western blots shown in Fig. 5f.** PLIN2 and α-tubulin.


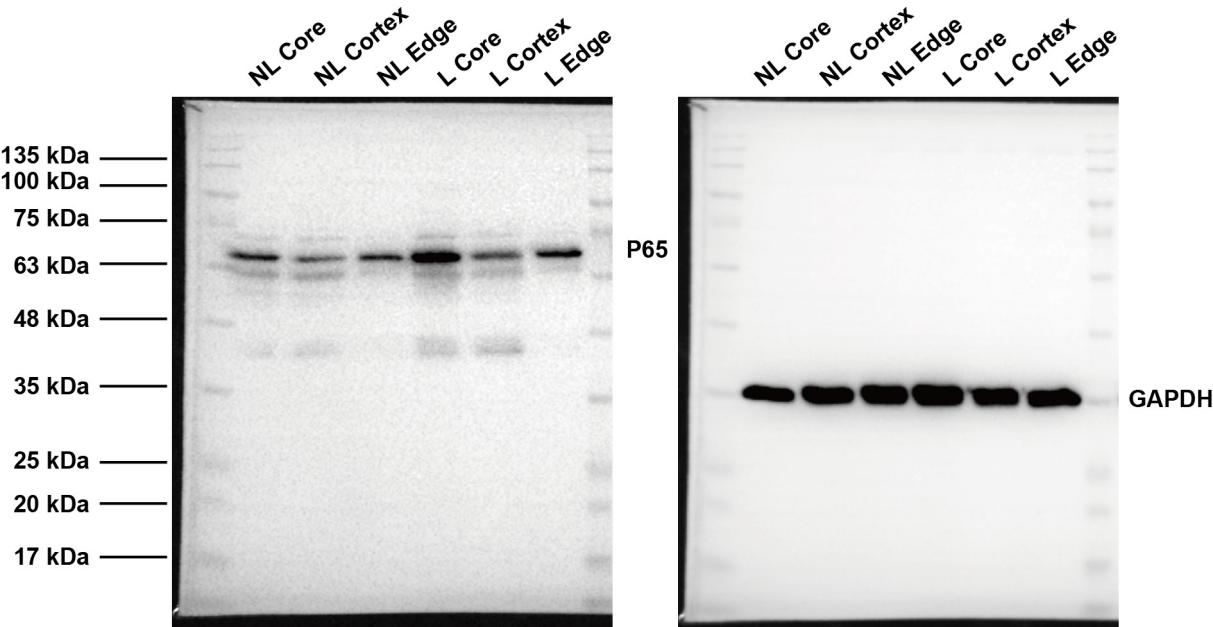


**Full scans of Western blots shown in Fig. 5f.** p65 and GAPDH.

**
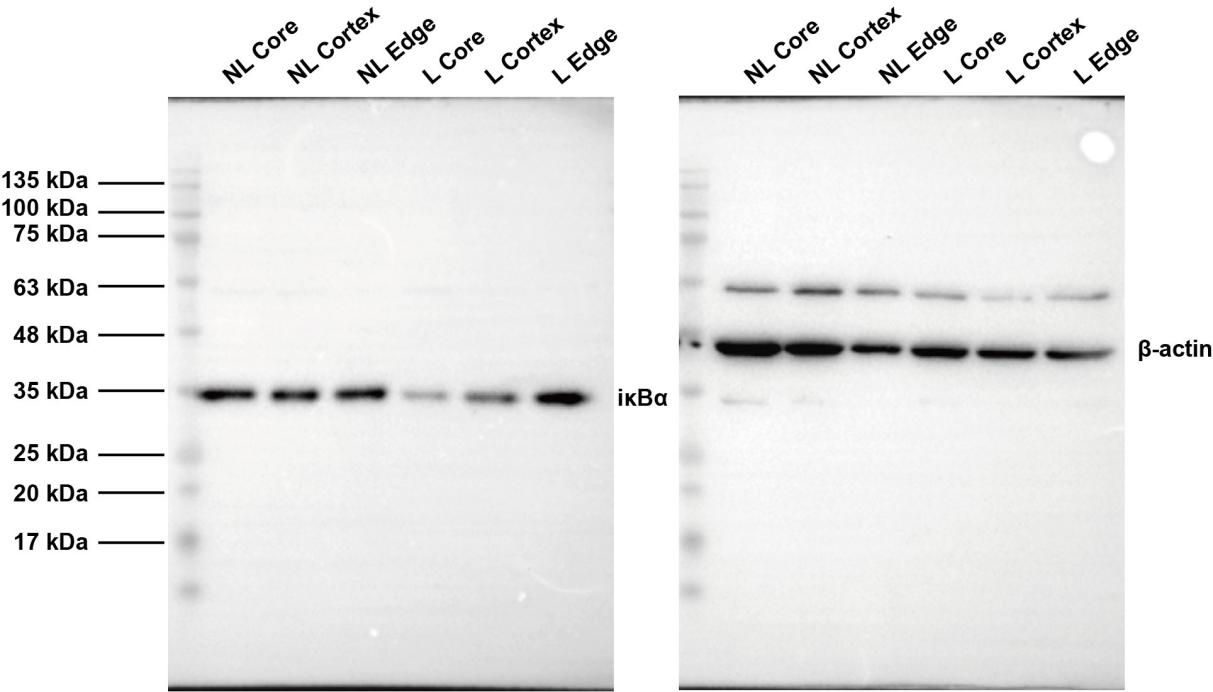
**

**Full scans of Western blots shown in Fig. 5f.** iκBα and β-actin.

**
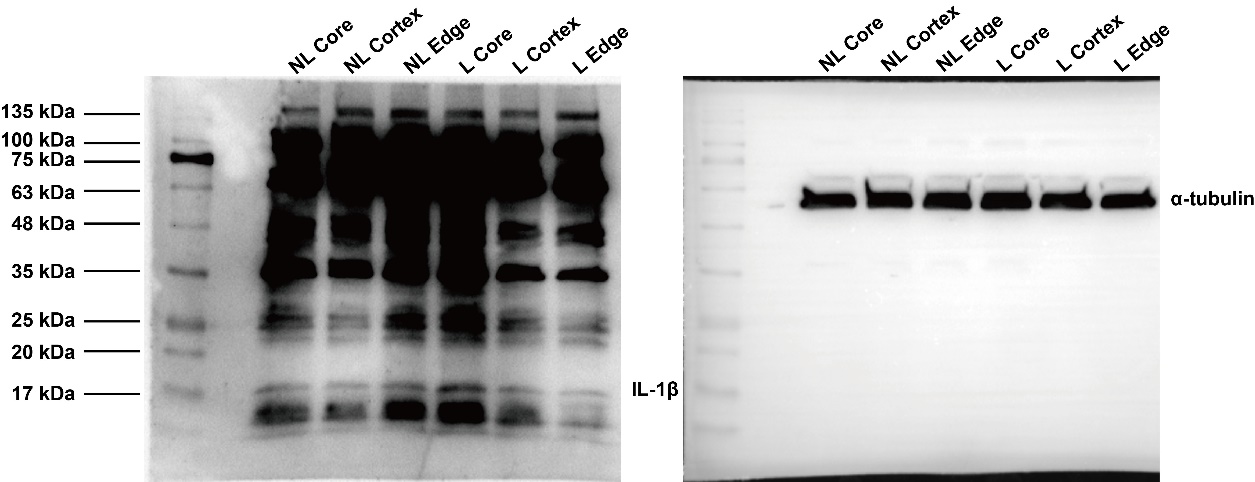
**

**Full scans of Western blots shown in Fig. 5f.** IL-1β and α-tubulin.


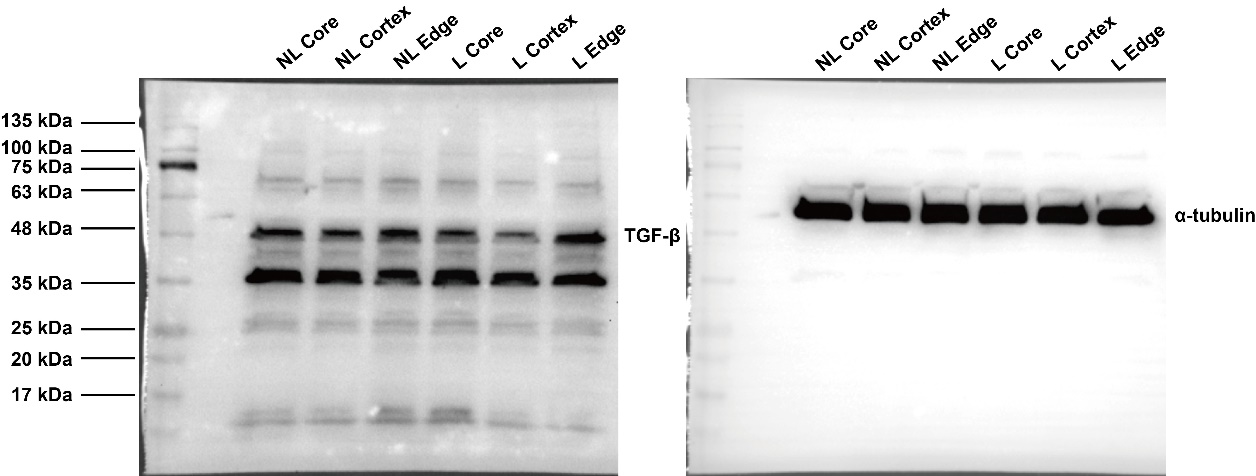


**Full scans of Western blots shown in Fig. 5f.** TGF-β1 and α-tubulin.

**Supplementary density plots of FACS**

**FACS sham group**


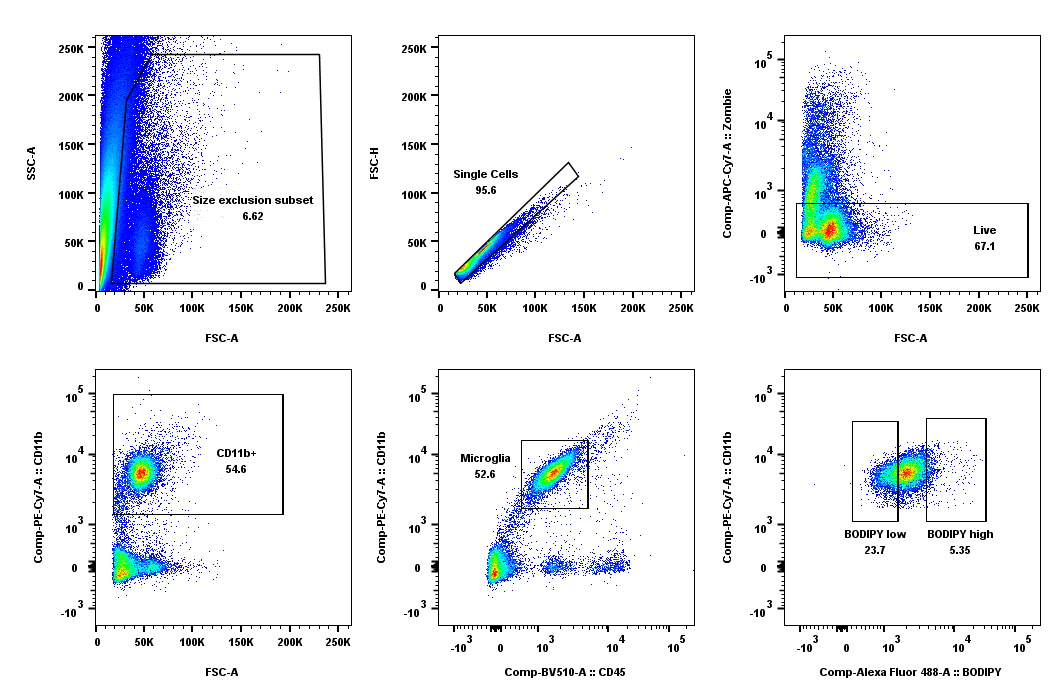


According to the gating strategy for flow cytometry analysis in **S-Figure 3**, we analyzed the ratio of lipid droplet-rich microglia (CD11b+ CD45int BODIPY+) in non-ischemic brain by the software FlowJo v. 10.8.1 (BD FACSDiva™).


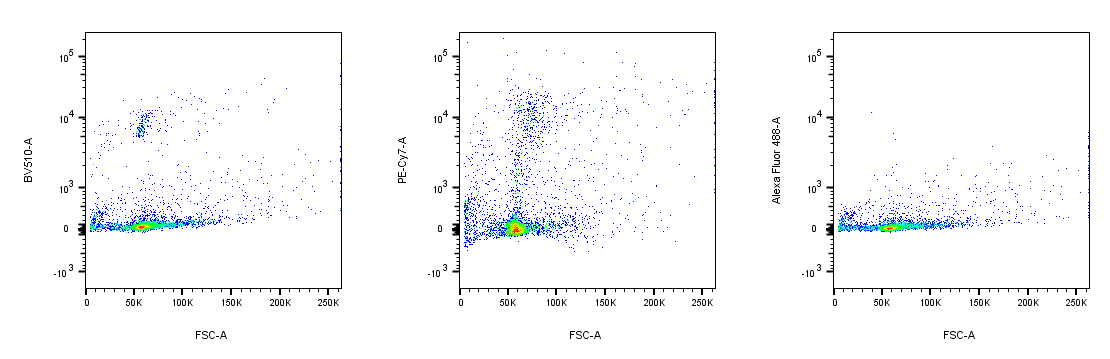


To exclude background autofluorescence, we performed the above unstained negative controls for CD45, CD11b and BODIPY. Abbreviations: LDRM, lipid droplet-rich microglia; MCAO, middle cerebral artery occlusion.

**FACS gating for CD68 and CD206**

**
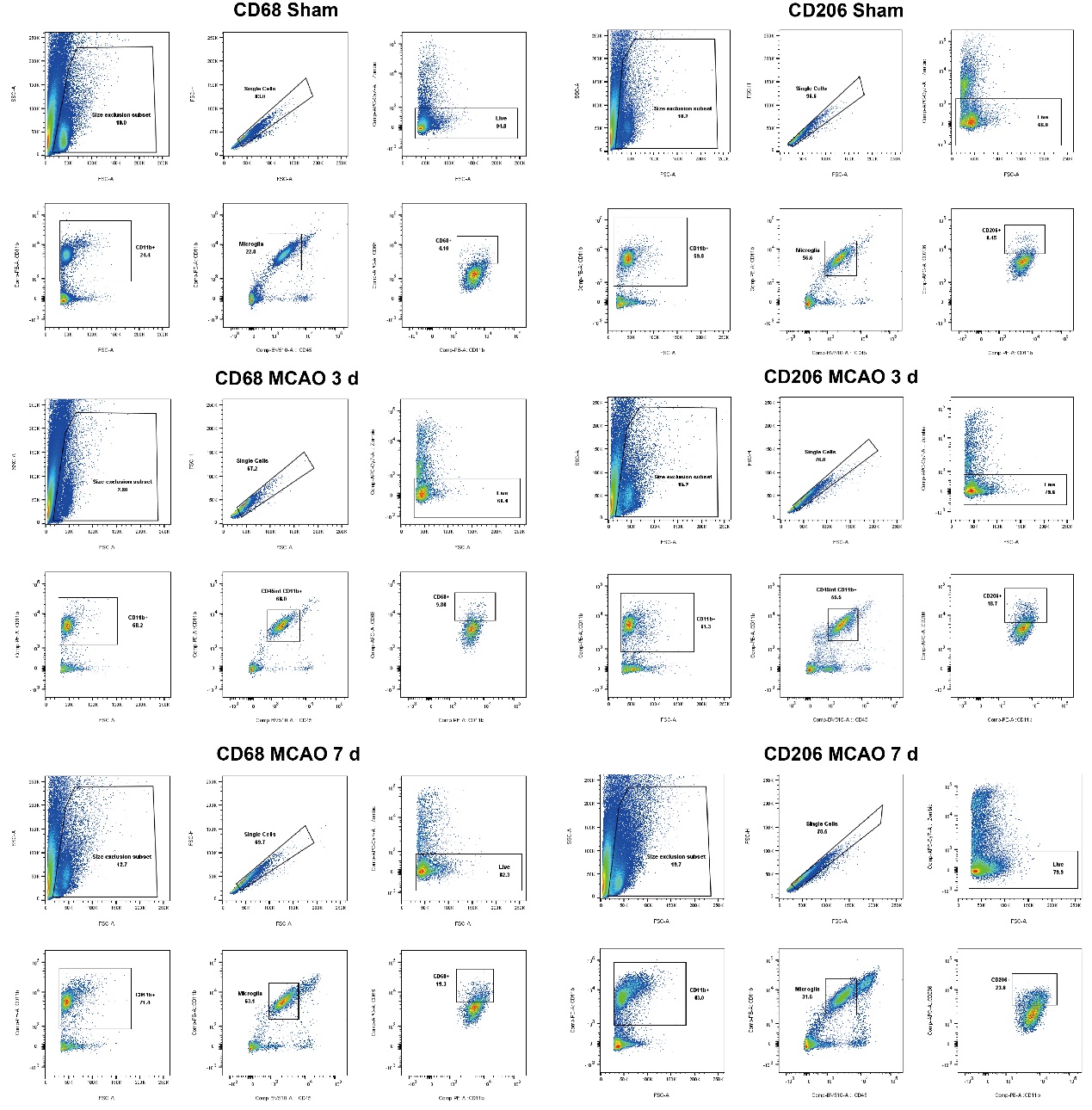
**

According to the gating strategy for flow cytometry analysis in **S-Figure 4**, we analyzed the ratio of lipid droplet-rich microglia (CD11b+ CD45int BODIPY+) in non-ischemic brain by the software FlowJo v. 10.8.1 (BD FACSDiva™).

**Supplementary negative and unstained controls of staining**

**Negative control of cell staining**


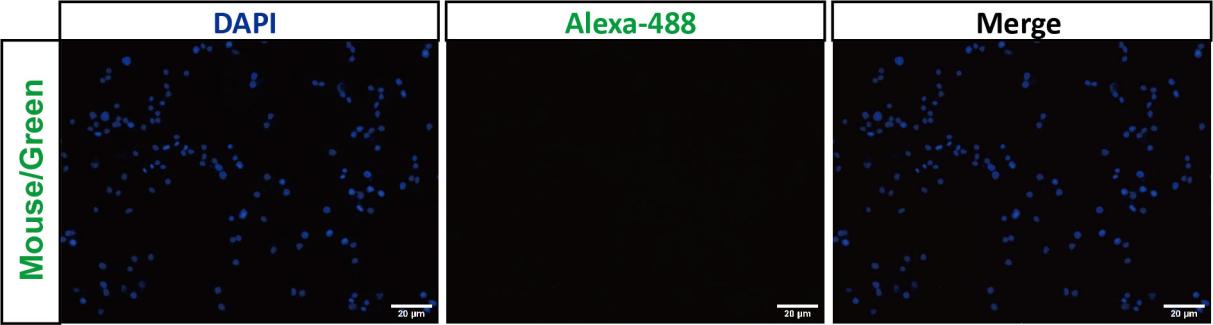


As a **negative control**, the secondary antibody AlexaFlour488 donkey anti-mouse IgG (1:250) was incubated with microglia, and there is no unspecific staining on the slides.


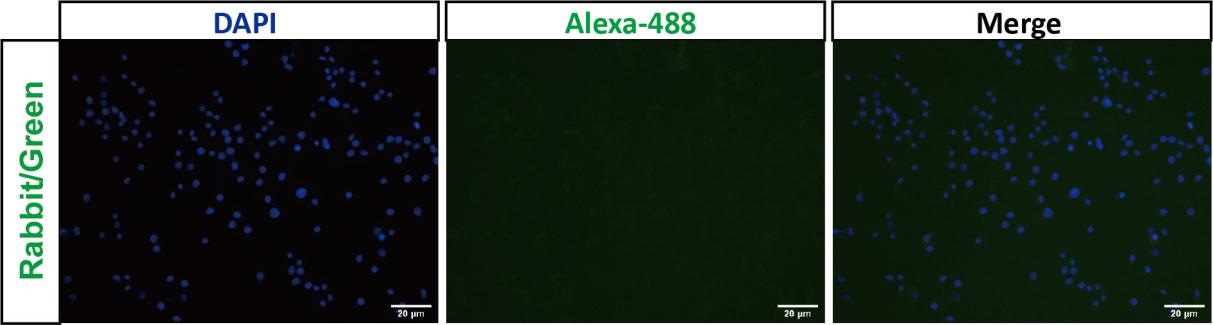


As a **negative control**, the secondary antibody AlexaFlour488 donkey anti-rabbit IgG (1:250) was incubated with microglia, and there is no unspecific staining on the slides.


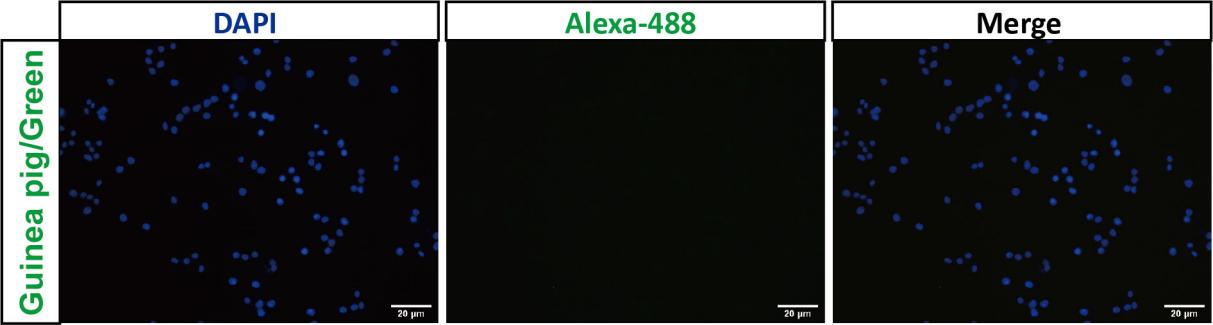


As a **negative control**, the secondary antibody AlexaFlour488 donkey anti-guinea pig IgG (1:250) was incubated with microglia, and there is no unspecific staining on the slides.


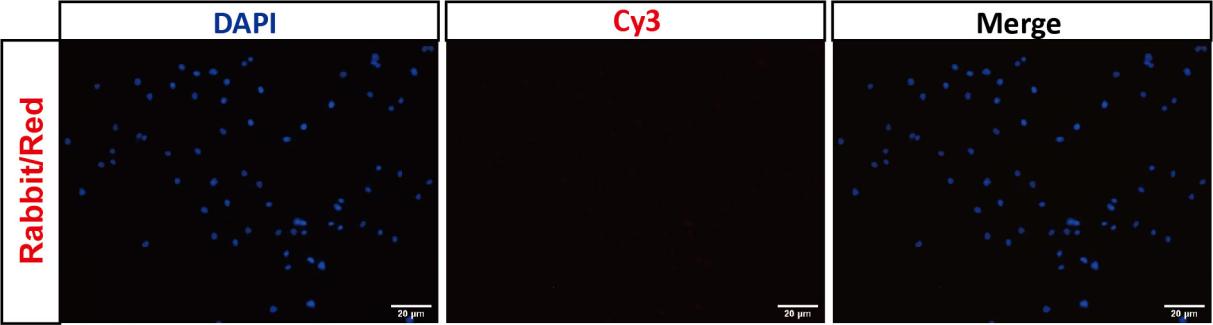


As a **negative control**, the secondary antibody Cy3 donkey anti-rabbit IgG (1:250) was incubated with microglia, and there is no unspecific staining on the slides.


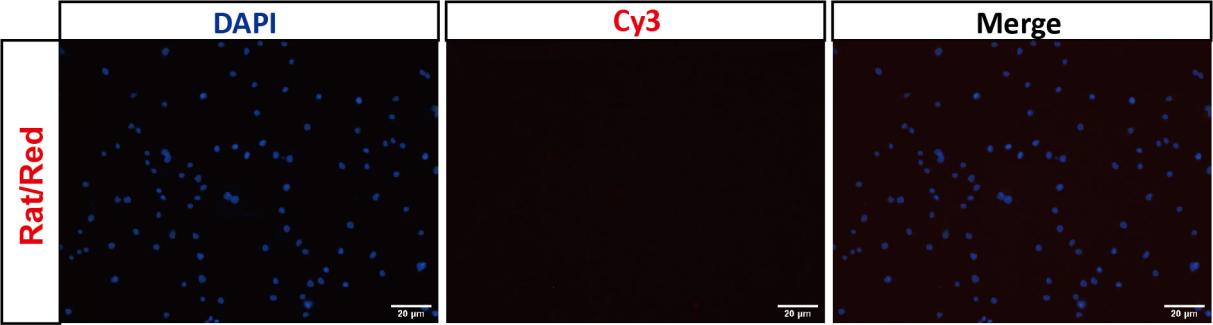


As a **negative control**, the secondary antibody Cy3 donkey anti-rat IgG (1:250) was incubated with microglia, and there is no unspecific staining on the slides.


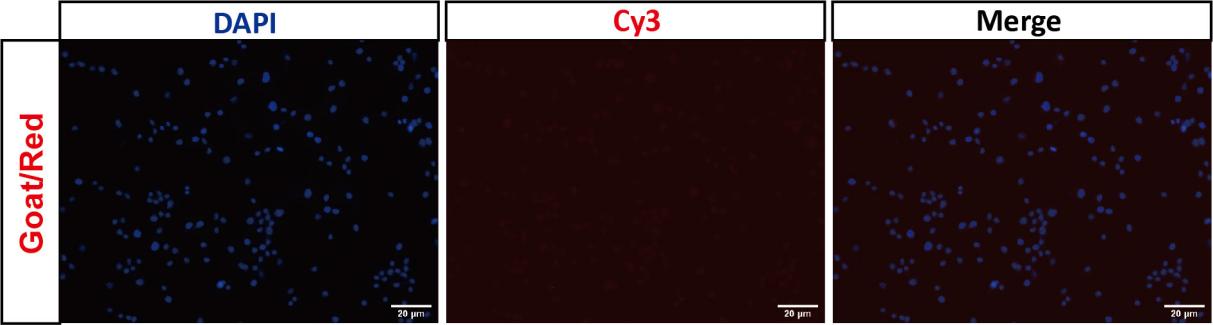


As a **negative control**, the secondary antibody Cy3 donkey anti-goat IgG (1:250) was incubated with microglia, and there is no unspecific staining on the slides.

**
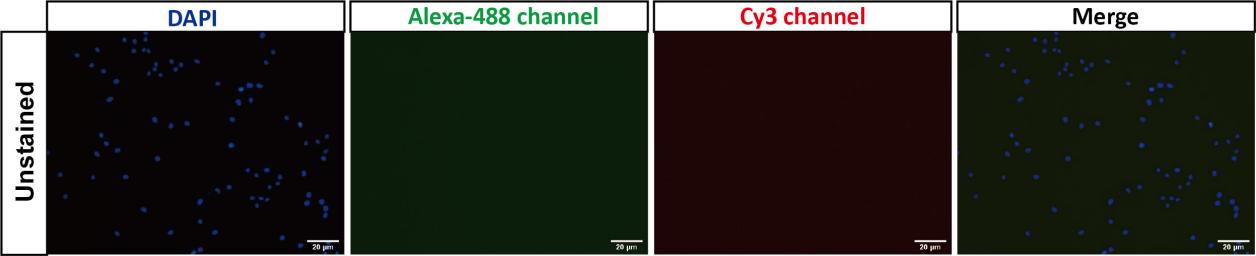
**

As an **unstained control**, only DAPI was detected with the microglia, in which does not yield any unspecific signal.

**Negative control of tissue slides**


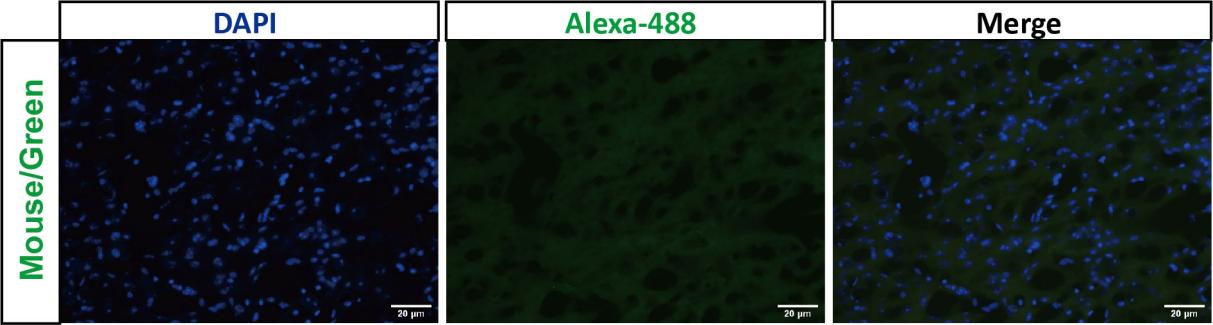


As a **negative control of staining**, the secondary antibody AlexaFlour488 donkey anti-mouse IgG (1:250) was incubated with tissue slides, which does not yield significant unspecific staining in these sections.


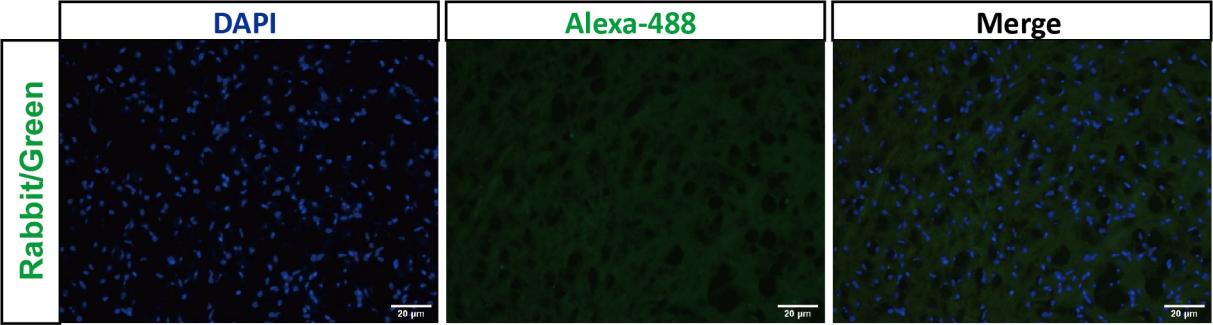


As a **negative control of staining**, the secondary antibody AlexaFlour488 donkey anti-rabbit IgG (1:250) was incubated with tissue slides, which does not yield significant unspecific staining in these sections.


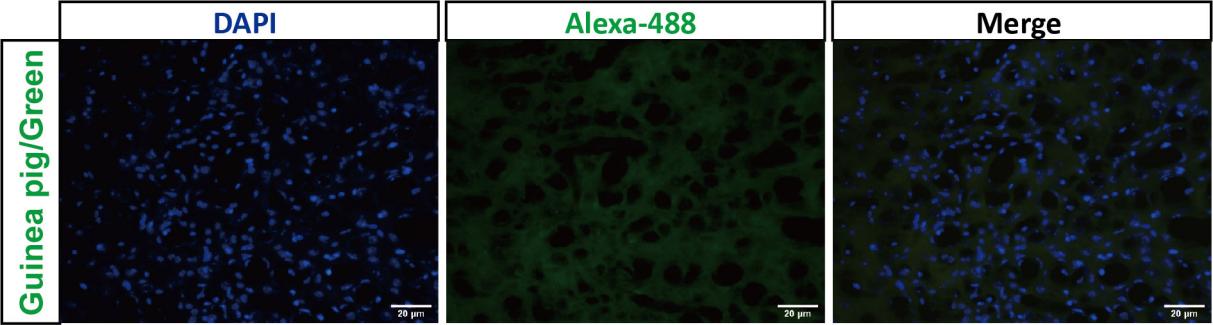


As a **negative control of staining**, the secondary antibody AlexaFlour488 donkey anti-guinea pig IgG (1:250) was incubated with tissue slides, which does not yield significant unspecific staining in these sections.


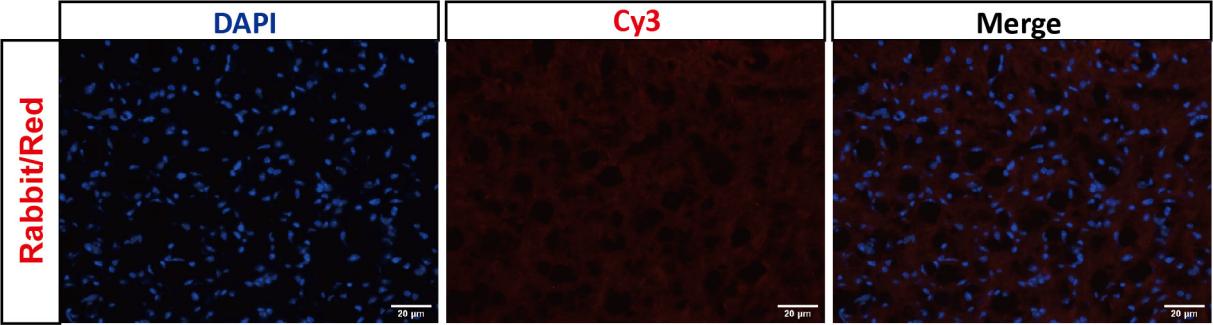


As a **negative control of staining**, the secondary antibody Cy3 donkey anti-rabbit IgG (1:250) was incubated with tissue slides, which does not yield significant unspecific staining in these sections.


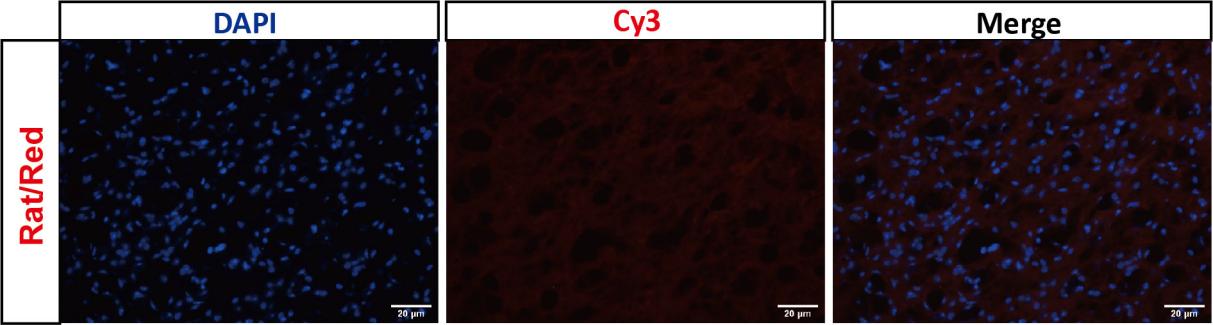


As a **negative control of staining**, the secondary antibody Cy3 donkey anti-rat IgG (1:250) was incubated with tissue slides, which does not yield significant unspecific staining in these sections.


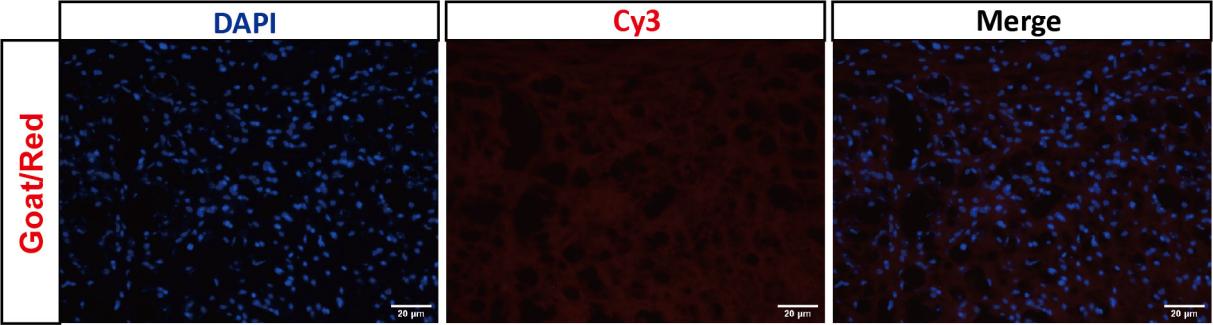


As a **negative control of staining**, the secondary antibody Cy3 donkey anti-goat IgG (1:250) was incubated with tissue slides, which does not yield significant unspecific staining in these sections.


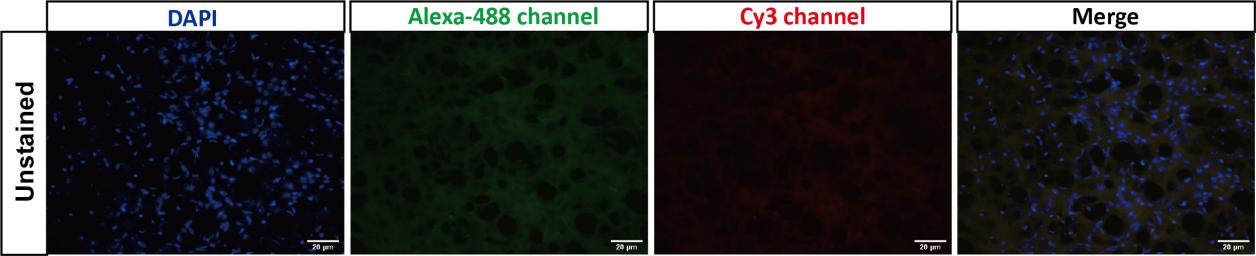


As an **unstained control**, only DAPI was detected with the tissue slides, which does not yield significant unspecific staining in these sections.
